# NanoCAGE analysis of the mouse olfactory epithelium identifies the expression of vomeronasal receptors and of proximal LINE elements

**DOI:** 10.3389/fncel.2014.00041

**Published:** 2014-02-18

**Authors:** Giovanni Pascarella, Dejan Lazarevic, Charles Plessy, Nicolas Bertin, Altuna Akalin, Christina Vlachouli, Roberto Simone, Geoffrey J. Faulkner, Silvia Zucchelli, Jun Kawai, Carsten O. Daub, Yoshihide Hayashizaki, Boris Lenhard, Piero Carninci, Stefano Gustincich

**Affiliations:** ^1^Area of Neuroscience, International School for Advanced Studies (SISSA)Trieste, Italy; ^2^RIKEN, Center for Life Science Technologies, Division of Genomic TechnologiesTsurumi-ku, Yokohama, Japan; ^3^Cluster in Biomedicine (CBM), AREA Science ParkTrieste, Italy; ^4^Bergen Center for Computational Science - Computational Biology Unit and Sars Centre for Marine Molecular Biology, University of BergenBergen, Norway; ^5^Cancer Biology Program, Mater Medical Research InstituteSouth Brisbane, QLD, Australia; ^6^School of Biomedical Sciences, University of QueenslandBrisbane, QLD, Australia; ^7^Department of Health Sciences, University of Eastern Piedmont “A. Avogadro,”Novara, Italy; ^8^RIKEN Preventive Medicine and Diagnosis Innovation ProgramWako, Saitama, Japan

**Keywords:** vomeronasal receptors, main olfactory epithelium, vomeronasal organ, VNO, MOE, V1Rs, V2Rs

## Abstract

By coupling laser capture microdissection to nanoCAGE technology and next-generation sequencing we have identified the genome-wide collection of active promoters in the mouse Main Olfactory Epithelium (MOE). Transcription start sites (TSSs) for the large majority of Olfactory Receptors (ORs) have been previously mapped increasing our understanding of their promoter architecture. Here we show that in our nanoCAGE libraries of the mouse MOE we detect a large number of tags mapped in loci hosting Type-1 and Type-2 Vomeronasal Receptors genes (V1Rs and V2Rs). These loci also show a massive expression of Long Interspersed Nuclear Elements (LINEs). We have validated the expression of selected receptors detected by nanoCAGE with *in situ* hybridization, RT-PCR and qRT-PCR. This work extends the repertory of receptors capable of sensing chemical signals in the MOE, suggesting intriguing interplays between MOE and VNO for pheromone processing and positioning transcribed LINEs as candidate regulatory RNAs for VRs expression.

## Introduction

Next-generation sequencing technologies have reshaped our understanding of the molecular constituents of cells and their regulatory elements. The majority of the mammalian genome is transcribed generating a vast repertoire of transcripts that includes protein-coding RNAs and a surprisingly similar number of non-coding RNAs (ncRNAs), the latter category harboring transcripts that can greatly differ in size and biogenesis and whose biological activities remain largely unexplored (Carninci and Hayashizaki, 2007; Forrest and Carninci, 2009; Mercer et al., 2009; Washietl et al., 2012). Furthermore, the combination of technologies to isolate discrete cell types or tissues with the information gathered with modern sequencing platforms has critically improved the resolution of genome-wide transcriptional profiling thus revealing new scenarios in which biological paradigms had often to be adapted and reformulated. An increasing number of these observations leads to a serious challenge to the concept of functional “ectopic” expression suggesting that proteins with defined biochemical activities may exert their biological function or acquire some new ones in previously unnoticed cells and tissues.

Cap Analysis of Gene Expression (CAGE) technology was previously developed for the systematic analysis of Transcription Start Sites (TSSs) in eukaryotic cells and tissues (Shiraki et al., 2003). It is based on sequencing cDNA copies of the 5′ends of mRNAs, of which the integrity is inferred by the presence of their cap. These sequences (“*tags*”) are sufficiently long to be aligned in most cases at a single location in the genome. The first position of this alignment identifies a base pair where transcription is initiated defining a TSS. The number of times a given tag is represented in a library gives an estimate of the expression level of the corresponding transcript. To expand this analysis to tiny amounts of *ex vivo* tissue and to the polyA^−^ fraction of RNAs we have developed nanoCAGE, a technology that miniaturizes the requirement of CAGE for RNA material to the nanogram range and which can be used on fixed tissues (Plessy et al., 2010). Using nanoCAGE we have previously shown that the well known oxygen carrier hemoglobin, previously believed to be specifically expressed in erythrocytes, is also selectively expressed in subtypes of dopaminergic neurons of the mesencephalon as well as in glial cells throughout the brain (Biagioli et al., 2009). Recently, we have used nanoCAGE to investigate the transcriptional landscape of the mouse Main Olfactory Epithelium (MOE) (Plessy et al., 2012).

The rodent olfactory system is composed by two major functional units, the MOE and the Vomeronasal Organ (VNO), and sensing of odor mixtures and pheromones are segregated into these two independent systems. Odorant detection in the MOE is mainly performed by Olfactory Sensory Neurons (OSNs) expressing Olfactory Receptors (ORs) while pheromones in VNO are revealed by two classes of Vomeronasal Sensory Neurons (VSNs) distinguished by the expression of a large repertory of Vomeronasal type-1 (V1Rs) and Vomeronasal type-2 Receptors (V2Rs) (Mombaerts, 2004; Zufall and Leinders-Zufall, 2007).

The extraordinary chemical diversity of olfactory ligands is matched in the mouse genome by more than 1100 intact OR genes (Buck and Axel, 1991; Zhang et al., 2007). With nanoCAGE we have confidently associated TSSs to 955 of them thereby defining a comprehensive picture of their promoter map at a single-base resolution (Plessy et al., 2012).

Here we show that further exploration of MOE nanoCAGE libraries reveals multiple evidences of transcription upstream of the annotated coding sequences for V1Rs and V2Rs. The expression of selected V1Rs and V2Rs has been validated by RT-PCR, RT-qPCR and *in situ* hybridization. Previous studies have highlighted the peculiar density of Repeat Elements (REs) and in particular Long Interspersed Nuclear Elements (LINE)s in V1Rs, V2Rs, and ORs loci (Kambere and Lane, 2009). Here we report that LINEs proximal to V1Rs and V2Rs are massively transcribed. These results significantly expand the potential repertory of chemoreceptors of the MOE and position transcribed LINE1 as candidate regulatory RNAs for VRs expression.

## Materials and methods

### NanoCAGE technology and protocol

For a detailed description of nanoCAGE please refer to Plessy et al. (2010).

### Animals, tissue preparation, laser capture microdissection and RNA quality control for NanoCAGE

This study has been approved by the Ethics Committee of the International School for Advanced Studies. All animal procedures have been applied in compliance with the “Directive 86/609/EEC on the protection of Animals used for Experimental and other scientific purposes” (European Commission, 2010).

For the first MOE collection, two C57BL/6J mice (a p20 male and a p21 female) were sacrificed by inhalation of carbon dioxide. After decapitation, the skin and the jaw were removed from the heads and the samples were left overnight in ZincFix fixative (BD Biosciences, CA, USA) diluted in DEPC-treated water. After a 4 h cryoprotection step in a 30% sucrose/1x ZincFix solution the heads were included in Frozen section medium Neg-50 (Richard Allan scientific, MI, USA) and left on liquid nitrogen-iced isopentane for 2 min. The frozen blocks were brought into a cryostat (Microm International, Walldorf, Germany) and left at −21°C for 30–120 min. Serial coronal sections of mouse heads (16 mm) were cut with a clean blade, transferred on PEN-coated P.A.L.M. MembraneSlides (P.A.L.M. Microlaser Tehnologies, Germany) and immediately stored at −80°C. For the second MOE collection, three C57BL/6J mice (two p12 males and a p13 female) were processed as described above. The total number of slices obtained in the two collections was 100, with 3/4 sections on each glass slide. The MOE was collected from mouse head sections by laser capture microdissection. Before processing, slides were left at RT and air dried for 2 min. The MOE was morphologically identified, marked, microdissected and catapulted with a Zeiss P.A.L.M. laser microdissection and pressure catapulting (LM-PC) microscope (Carl Zeiss Inc., Germany) in P.A.L.M. tubes with adhesive caps (PALM Microlaser Technologies GmbH, Germany). After the harvest, 10 μl of lysis buffer (Stratagene, CA, USA) were added in each cap; the samples were left capsized at RT for 10 min, centrifuged at 6000 × g for 10 min and stored at −80°C. RNA was then extracted, DNase-treated and purified with Absolutely RNA Microprep kit (Stratagene, CA, USA) following manufacturer's instruction. After the elution step in nuclease-free water (Ambion, TX, USA) the concentration of the samples was measured with ND-1000 spectrophotometer; 500 pg of each sample were run on a 2100 Bioanalyzer (Agilent, CA, USA); samples with high RNA quality were pooled (26 out of 30 samples).

### Conservation-based mapping of NanoCAGE TCs on V1Rs and V2Rs genomic regions

Most of the TSSs around V1Rs and V2Rs genes were mapping to repeats, mostly overlapping with LINE1, which is in accordance with the observation made by Kambere and Lane (Kambere and Lane, 2009).

For the mapping purposes, we have considered TCs that were not overlapping repeats. Many of the non-repeat TCs overlapped with the opossum and platypus conserved blocks and alongside the rat conserved blocks (Table 3). This was in agreement with the observation that the number and complexity of V1Rs and V2Rs in rodents, platypus and opossum are very similar to one another (Grus et al., 2005, 2007). We have clustered the conserved blocks on the upstream of V1Rs and V2Rs and looked for TCs overlapping with them. The mapping method is similar to the TC-to-OR genes mapping method used in (Plessy et al., 2012). However, since there are many TCs with opossum and platypus conservation, we added these species to the conserved block clusters. Conserved blocks from mouse (mm9) against the other species (rat, human, horse, dog, opossum and platypus) were clustered together if they were at least 4000 bp away. These clusters were mapped to upstream regions of vomeronasal genes. If upstream regions were overlapping with another refseq gene on the same strand, the upstream region clipped accordingly. Furthermore, if the conserved upstream region is longer than 15000 bp we clipped the region to 15000 bp from the gene start. The TCs (non-repeat overlapping) mapping to these conserved upstream regions were ranked by their expression and the TC with the highest expression mapped to the gene. All of the V1Rs and V2Rs genes were overlapping with a conserved upstream region. The median length of conserved upstream regions was 4478 bp. 30.2% of V2Rs and 53.1% of V1Rs mapped to a non-repeat TC overlapping a conserved block cluster

### Animals and preparation of tissues for RT-PCR and qRT-PCR NanoCAGE data validation

For RT-PCR, five adult C57B6/J mice (Charles River Laboratories International, Japan) were killed by carbon dioxide inhalation and decapitated; the MOE and the VNO were dissected from the heads, added with TRIzol reagent (Invitrogen, USA), immediately snap-frozen in liquid nitrogen and stored at −80°C.

For qRT-PCR, the MOE and VNO were dissected from P21 (2 males, 2 females) and P50 (2 males, 2 females) C57BL/6J mice (Charles River Laboriatories International, Japan) added with TRIzol reagent, immediately snap-frozen in liquid nitrogen and stored at −80°C. The quality of RNA samples was assessed with 2100 Bioanalyzer (Agilent Technologies, USA)

### RT-PCR

First strand cDNA synthesis was performed as described elsewhere (Pifferi et al., 2006). PCR was carried out by adding 1 μ l of first strand reaction to a mix containing 5u of Takara Taq DNA polymerase, 10× buffer, dNTPs mix 2.5mM each (all reagents from Takara, Japan), 50 pmol forward and reverse primers and nuclease-free water to a final volume of 50 μ l. Forward exon-spanning primers were used to avoid unwanted amplification of residual genomic DNA.

### qRT-PCR

cDNAs were synthesized as previously described (Pifferi et al., 2006). Quantitative real-time PCR experiments were performed with SYBR Premix Ex Taq II (Takara, Japan) in a volume of 10 μ l on 384-wells plates using up to 30 ng of cDNA per reaction. Amplification and scanning were performed in a 7900HT Fast Real-Time PCR system (Applied Biosystems, USA). Standard curves with 3-points serial dilutions for each target were included on each plate to allow for inter-plate comparisons. RT- and PCR mix with no cDNA were included as negative controls.

After normalization with Gapdh *Ct* values, the inverse power of the normalized *Ct* values for each target were used to calculate the mean and the standard deviations plotted in the shown figures.

### Animals and preparation of tissues for *in situ* hybridization

18–25 days old C57BL/6J mice were anesthetized with a 0.75 g/kg urethane solution injection and perfused intracardially with a 4% parafomaldehyde/PBS1x solution pH7.4 prepared in DEPC-treated water (PFA). After the perfusion mice were decapitated, the skin and the lower jaw were removed and the sample was put in the same PFA solution O/N at 4°C. Samples were decalcified for 12 h in a 0.5 M EDTA pH8.0/PBS1x solution prepared in DEPC-treated water. Cryoprotection was carried out in 10% sucrose/PBS1x for 2 h, 20% sucrose/PBS1x for 2 h and 30% sucrose/PBS1x 3 h to O/N at 4°C. Heads were included in Frozen section medium Neg-50 (Richard Allan scientific, MI, USA) and left on liquid nitrogen-iced isopentane for 2 min. The frozen blocks were left into a cryostat (Microm International, Walldorf, Germany) at −21°C for 30–120 min. Serial coronal sections of mouse heads (16 μm) were cut with a clean blade, and transferred on Superfrost Plus glass slides (Menzel-Glaser, Menzel GmbH and co KG, Germany) with a maximum number of three slices per slide. Sections were air-dried for 120 min and immediately used for hybridization or stored at −80°C.

### Biotin-labeled riboprobes preparation

PCR products were cloned in pGEM T-easy vector (Promega, WI, USA). 10 μ g of each plasmid containing the specific PCR product were linearized with SacII (NEB) or with SalI (Promega) restriction enzymes for transcription with SP6 and T7 promoter, respectively. After an O/N incubation at 37°C, samples were cleaned with the PCR purification kit (Qiagen). For riboprobes transcription, 1 μ g of each digested plasmid was added to a mix containing 2 μ l of RNA BIO-labeling mix (Roche Applied Science, Germany), 20 units of SP6 or T7 RNA polymerases, 5× transcription buffer (both from Promega, WI, USA), 0.1 M DTT, 20 units of SuperaseIn RNase inhibitor (Ambion) and nuclease-free water (Ambion) in a volume of 20 μ l. After 2 h of incubation at 37°C, the transcription reaction was stopped by adding 2 μ l of 0.2 M pH 8.0 EDTA. RNA probes were precipitated by adding 1.25 μ l of 4 M LiCl and 37.5 μ l of absolute ethanol cooled at −20°C and stored for 2 h at −80°C. Samples were centrifuged at 20.000 × g for 30 min at 4°C and RNA pellets were washed once with 70% ethanol, briefly air-dried and resuspended in 50 μ l of nuclease-free water with the addition of 20 units of Superase RNase inhibitor (Ambion).

### *In situ* hybridization protocol

*In situ* hybridizations were carried out as previously described by Ishii et al. (2004). Please refer to Supplementary Data for details.

### Detection of biotin-labeled RNA probes

For the detection of BIO-labeled RNA probes Cy3-tyramide or Cy5-tyramide reactions (Perkin Elmer Life Sciences, Boston, MA) were used. Stainings were analyzed and acquired with a Leica TCS LSI confocal microscope or with a Leica DM6000B light microscope (Leica Microsystems GmbH, Germany). Images were resized with Photoshop CS3 software (Adobe Systems Incorporated, CA, USA). Please refer to Supplementary Data for details.

## Results

### NanoCAGE detects active transcription at V1Rs and V2Rs loci in the mouse MOE

The MOE was harvested with LCM from adjacent histological sections of C57BL/6J mice at 12 or 20–22 post-natal days. Samples were processed with zinc-fix, an optimal fixative for both tissue morphology and RNA integrity preservation. Two nanoCAGE libraries were synthesized from independent harvests and deeply sequenced using Illumina technology, yielding a total of 53,158,862 tags with a length of 25 bp and corresponding to the very 5′-end of MOE capped transcripts. 31,031,749 tags were confidently mapped to the mouse genome (Faulkner et al., 2008; Hashimoto et al., 2009). The mapped nanoCAGE tags were clustered and aggregated into Tag Clusters (TCs) (Carninci et al., 2006), and the data were unified in publicly accessible tracks that can be uploaded in UCSC Genome Browser (see Supplementary Data). This dataset displayed evidence for the expression of 87.5% of OR genes (955/1092), defining a comprehensive description of their TSSs and core promoters (Plessy et al., 2012).

When screened for the expression of additional candidate receptors for chemical sensing, the nanoCAGE libraries displayed multiple evidences of TCs mapping upstream of the annotated coding sequences for V1Rs genes and in close proximity to the 5′-end of V2Rs genes. We found TCs mapping upstream of 112/191 V1Rs and 96/123 V2Rs; the overall number of TCs mapping in V1Rs and V2Rs genes loci was 577 and 812, respectively, with an average TPM score of 0.53 and 0.12 (Supplementary Table S1). Furthermore, TCs were also associated to the 5′ TSS of core components of the vomeronasal transduction pathway such as *Gαo* (544 reads), *Gαi2* (64 reads), *Trpc2 isoform 1* (NM_011644, 261 reads) and *Trpc2 isoform 2* (NM_001109897, 243 reads); these data are summarized in Supplementary Table S2.

### Analysis of TSSs associated to V1Rs and V2Rs expression in the mouse MOE

The majority of TCs in proximity to V1Rs and V2Rs mapped on REs (Table 1) and more specifically on LINE1s (Table 2 and Supplementary Table S3). Both young and ancestral LINE1 families were expressed.

**Table 1:** **Percentage of repeat and non-repeat overlapping TCs around V1Rs and V2Rs**.

	**% of CAGE TCs mapped in proximity of V1R and V2R genes**	
**VRs family**	**Repeat overlapping**	**Non-repeat overlapping**
V1Rs	56.3% (325/577)	43.6% (252/577)
V2Rs	57.1% (464/812)	42.8% (348/812)

**Table 2:** **Classes of Repeat Elements overlapping with TCs mapping around V1R and V2R receptors**.

	**% repeats overlapping with TCs around Vomeronasal genes**				
**VRs family**	**LINEs (%)**	**LTRs (%)**	**SINEs (%)**	**Simple_repeat (%)**	**Others (%)**
V1Rs	66.4	21.8	5.8	2.1	4.3
V2Rs	61.8	26.5	3.8	3.4	5.5

**Figure 1:**
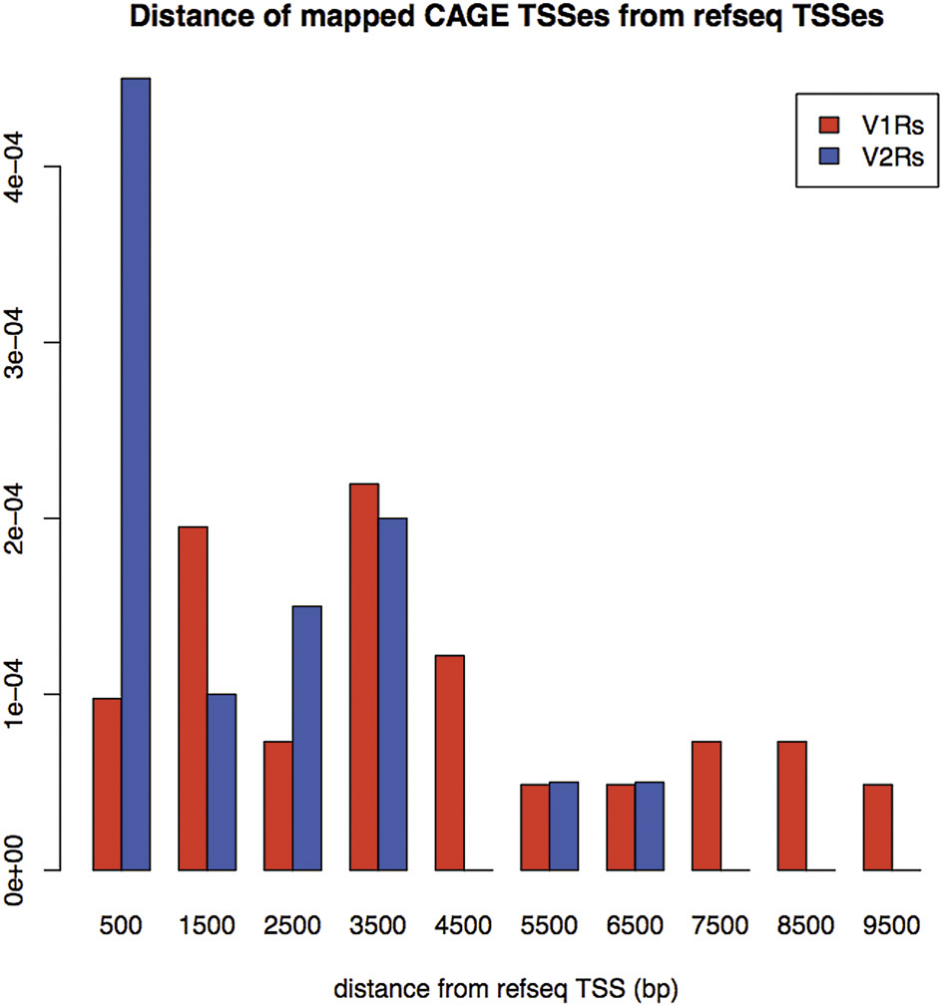
**Density of distances between mapped TCs identified by nanoCAGE and associated with VRs, and annotated TSS for RefSeq V1Rs and V2Rs**. On the X-axis: Distance from RefSeq TSSs in basepairs; on the Y-axis: Frequency of mappings. The mean distance for V1Rs is higher than for V2Rs, in agreement with the observation that most of V1Rs genes are only annotated as single-exon ORFs whereas V2Rs genes have annotated exon-intron structures.

A large portion of TCs not mapping on repeats (30.2% of V2Rs and 53.1% of V1Rs) overlapped with genomic regions conserved in opossum, platypus and rat genomes (Table 3). This is not surprising since number and genomic complexity of V1Rs and V2Rs in rodents, platypus and opossum are similar (Grus et al., 2007). We then sought to associate with confidence a particular TC to its downstream VR transcript, thus defining its TSS; for this analysis, only TCs not mapping to repeats were taken in consideration. We clustered the conserved regions of rat, platypus and opossum directly upstream of each VR gene. The resulting median length of these conserved sequences was 4478 bp; for V1Rs the median distance between the TCs selected by this association procedure and the annotated gene start was 3592 bp while for the V2Rs it was 842 bp (Figure 1). This was expected since most of the V1Rs are only annotated as single-exon open reading frames (ORFs) as in the case of ORs, whereas most V2Rs have annotated exon-intron structures that are defective of the untranslated regions (UTRs) at both the 5′ and 3′-ends. A detailed description of all the TCs mapping in V1Rs and V2Rs genes loci is presented in Supplementary Table S1.

**Table 3:** **Percentages of non-repeat TCs overlapping with species-specific conserved blocks; a given TC can overlap with different species-specific blocks at the same time**.

	**% of conserved non-repeat TCs around VR genes**						
**VRs family (%)**	**Rat (%)**	**Human (%)**	**Horse (%)**	**Dog (%)**	**Opossum (%)**	**Platypus (%)**	**Not conserved in these species (%)**
V1Rs	34.6	9.2	6.1	3.8	27.9	18.2	23.0
V2Rs	32.1	14.3	11.2	13.1	17.6	11.4	30.7

### Validation of NanoCAGE data and single TSSs by RT-PCR

**Figure 2:**
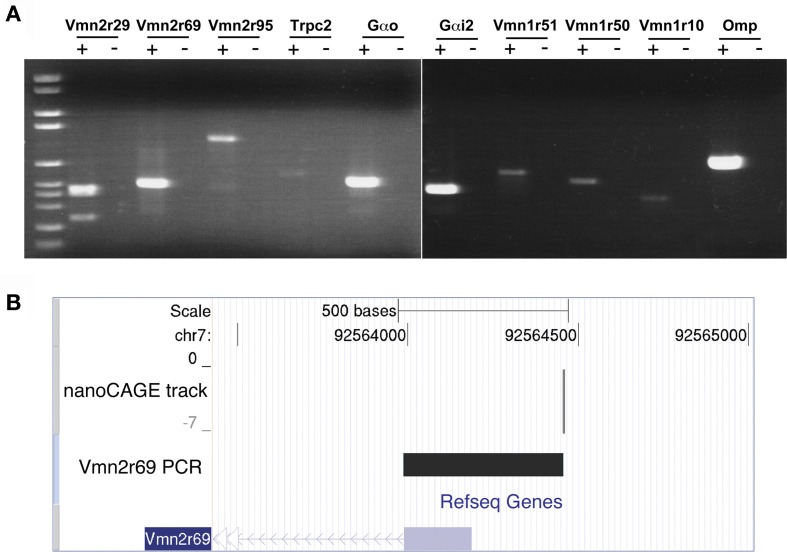
**Validation of nanoCAGE data by RT-PCR confirms the expression in the MOE of V1Rs, V2Rs, and key components of the pheromone transduction pathway**. **(A)** RT-PCR validation was carried out starting from the same total RNA sample of the MOE used for the nanoCAGE workflow. V1Rs and V2Rs to be validated were chosen by interest or on the basis of their TPM score from the list of all expressed VRs detected by nanoCAGE. DNA molecular weight Marker VI is used as DNA ladder (Roche Applied Science). **(B)** The TSS of *Vmn2r69* identified by nanoCAGE was validated by RT-PCR with a forward primer designed in proximity of the TSSs and a reverse primer designed on the first exon. The sequence of the RT-PCR product is shown uploaded in the UCSC Genome Browser along with the nanoCAGE data.

We validated the nanoCAGE data of selected genes by performing a standard RT-PCR starting from the same total RNA sample used for the synthesis of the nanoCAGE libraries. We amplified transcripts for the V2Rs family (*Vmn2r29*, *Vmn2r69*, and *Vmn2r95*), the V1Rs family (*Vmn1r51*, *Vmn1r50*, *Vmn1r10*) and the components of the vomeronasal transduction pathway (*Gαo*, *Gαi2*, and *Trpc2*); *Omp* was chosen as a positive control for its high expression in the MOE (Figure 2A). Because of the high level of intra-cluster homology, RT-PCRs for *Vmn2r29* and *Vmn2r95* amplified additional V2Rs including *Vmn2r30*, *Vmn2r31*, and *Vmn2r42* on chromosome 7 as well as *Vmn2r104* and *Vmn2r107* on chromosome 17. We also validated the expression of *Vmn2r26*, the only V2Rs that has been experimentally proven to bind a MHC class I peptide in the VNO (Leinders-Zufall et al., 2009) and for which nanoCAGE identified a sharp TSS mapped 105 bases upstream of the annotated Refseq gene. Cloning and sequencing confirmed the identity of all validated transcripts.

The transcription starting sites identified by nanoCAGE for receptors *Vmn1r228, Vmn2r3*, *Vmn2r69*, and *Vmn2r76* were confirmed by RT-PCRs with forward primers designed immediately downstream of the TSSs and reverse primers within the respective Refseq sequences. The sequence of the PCR products that confirms the TSS for *Vmn2r69* is shown uploaded in the UCSC Genome Browser as a representative example of validation in Figure 2B; the sequences of all the obtained amplicons are available in Supplementary Data.

### *In situ* hybridization confirms the expression of VRs in a consistent number of cells throughout the mouse MOE

To explore the identity of the cells expressing VRs transcripts we performed fluorescent *in situ* hybridization with specific riboprobes synthesized from a selection of RT-PCR products. Sense and antisense probes were assayed on serial MOE cryosections of mice matching in age and sex those used for the construction of nanoCAGE libraries.

**Figure 3:**
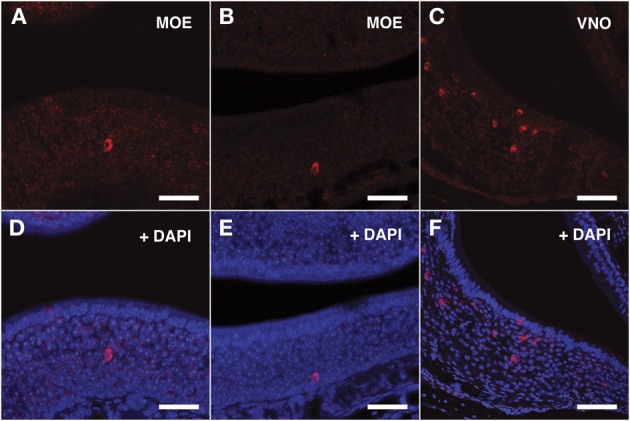
***In situ* hybridization with *Vmn1r201* riboprobes on serial sections of the MOE**. **(A,B)** Several cells throughout the MOE are revealed by the antisense *Vmn1r201* riboprobe. These cells are mainly found in the middle and basal layers of the MOE. The control sense probe for *Vmn1r201* did not display any detectable staining in the MOE or VNO. **(C)** The *Vmn1r201* antisense riboprobe hybridizes as expected with a high number of cells throughout the VNO. **(D–F)** Panels **(A–C)** merged with stained nuclei (DAPI). Scale bars: 60 μm.

For the V1Rs family we investigated the expression of *Vmn1r201* on the basis of its sharp TSS identified by nanoCAGE. Due to high sequence homology, the antisense riboprobe for *Vmn1r201* was theoretically able to hybridize also to *Vmn1r215* and *Vmn1r218* mRNA, although for *Vmn1r218* no significant TSSs were detected in the nanoCAGE libraries. The *Vmn1r201* riboprobe decorated a discrete number of cells residing in the basal and middle layer of the epithelium with no zonal preference (Figures 3A,B,D,E).

**Figure 4:**
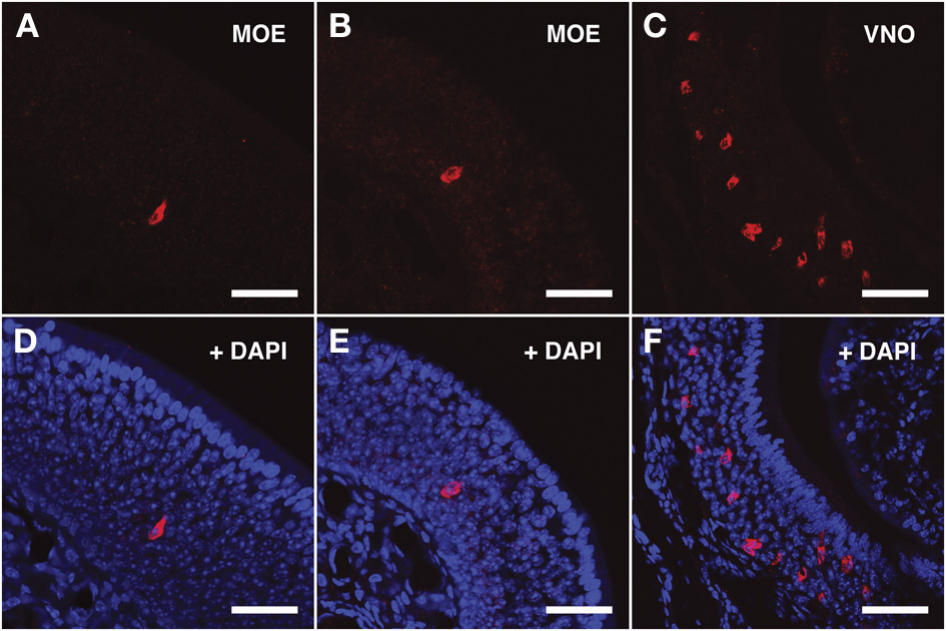
***In situ* hybridization with *Vmn2r26* riboprobes on serial sections of the MOE**. **(A,B)** The *Vmn2r26* antisense riboprobe hybridizes with a high number of cells throughout the MOE, mostly located in the basal and medial layers. **(C)** The *Vmn2r26* antisense riboprobe specifically stains a high number of cells throughout the VNO. **(D–F)** Panels **(A–C)** merged with stained nuclei (DAPI). Scale bars: **(A,B,D,E)** 50 μm; **(C,F)** 60 μm.

For the V2Rs family we synthesized riboprobes for *Vmn2r26* and *Vmn2r69*. Due to the high level of homology among V2Rs genes, the antisense probe for *Vmn2r26* was able to hybridize to *Vmn2r19*, *Vmn2r23*, and *Vmn2r24*, sharing a homology rate ≥80%. All these V2Rs presented non-repeats mapping TCs in close proximity to the annotated RefSeq 5′ end. The riboprobe for *Vmn2r69* was specific for this receptor. *Vmn2r26* antisense riboprobe distinguished numerous cells in the middle layer as well as a group of cells located in the basal layer of the MOE; all of them displayed morphological features remarkably similar to those of the OSNs. *Vmn2r26*^+^ cells were mainly localized in the central/dorsal turbinates of the MOE (Figures 4A,B,D,E).

**Figure 5:**
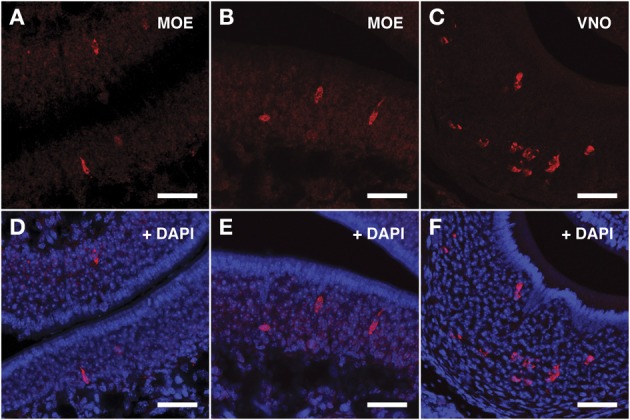
***In situ* hybridization with *Vmn2r69* riboprobes on serial sections of the MOE**. **(A,B)** Cells detected with the *Vmn2r69* riboprobe reside in the middle layer of the MOE, and are mostly found in dorsal turbinates. **(C)** The *Vmn2r69* antisense riboprobe hybridizes with a high number of cells in the VNO. **(D–F)** Panels **(A–C)** merged with stained nuclei (DAPI). Scale bars: 60 μm.

The ISH results obtained with *Vmn2r69* riboprobe were comparable to those obtained for *Vmn2r26* in terms of cell morphology, while *Vmn2r69*^+^ cells were only observed in dorsal turbinates (Figures 5A,B,D,E). Supplementary Figure S1 shows *in situ* hybridization results for *Vmn2r26* and *Vnm2r69* on dorsal/central turbinates of the MOE where the highest density of positive cells was observed. A complete count of *Vmn2r26*^+^ and *Vmn2r69*^+^ cells was performed in the whole MOE of two sexually mature 45-days old males C57BL/6J, resulting in a total of 543 and 332 positive cells, respectively.

The specificity of the *Vmn1r201, Vmn2r26*, and *Vmn2r69* antisense riboprobes was confirmed by the expected staining of numerous cells in the basal and upper layer of the VNO (Figures 3C–F, 4C–F, 5C–F respectively); the control sense probes for each target gene were extensively tested on serial sections of the MOE and the VNO, with no detectable signal in both tissues for all probes; a representative set of images for the negative controls is presented in Supplementary Figure S2.

### Validation of expression levels of *Vmn2r26* and *Vmn2R69* in the MOE of P21 and P50 mice by qRT-PCR and comparison with expression levels of selected ORs

**Figure 6:**
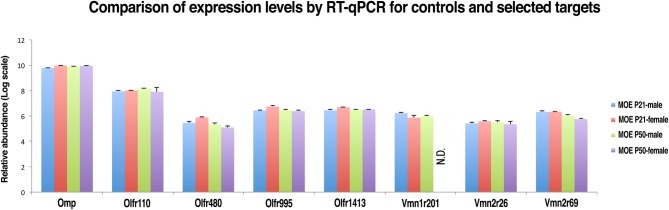
**Validation of nanoCAGE data by qRT-PCR confirms that the expression levels of selected VRs in the MOE of young and adult mice are comparable to the expression levels of ORs genes with similar tag counts in nanoCAGE libraries**. The qRT-PCR validation was performed in triplicates on RNA purified from the dissected MOE and VNO of P21 (males *n* = 5, females *n* = 5) and P50 (males *n* = 5, females *n* = 5) C57BL/6J mice. All primers used were designed in an exon-spanning fashion; the *Ct* values of each target were normalized on Gapdh *Ct* values. The expression levels in the VNO and the copy number calculation are shown in Supplementary Figure S3.

In order to confirm the reliability of nanoCAGE in detecting the expression of selected VRs genes we performed a real-time quantitative RT-PCR (qRT-PCR) on *OMP* (TPM = 157), an OR gene with an high expression level (*Olfr 110*, TPM = 22.7), three vomeronasal receptor genes (*Vmn1r201*, TPM = 0.18; *Vmn2r26*, TPM = 0.15; *Vmn2r69*, TPM = 0.22) and three OR genes with a low tag count comparable to the selected vomeronasal receptor targets (*Olfr480*, TPM = 0.25; *Olfr995*, TPM = 0.22; *Olfr1413*, TPM = 0.16) (Figure 6). qRT-PCR reactions were performed in triplicates on total RNA purified from dissections of the MOE and VNO of P21 (males *n* = 5 and females *n* = 5) and P50 (males *n* = 5 and females *n* = 5) C57BL/6J animals. As a negative control we performed qRT-PCR on the same targets using as a template total RNA purified from mouse liver. After copy numbers count and normalization with Gapdh *Ct* values, we were able to quantify mRNAs for *Olfr995*, *Olfr480*, *Olfr1413*, *Vmn2r26*, and *Vmn2r69* and to confirm that qRT-PCR data are consistent with the expression levels detected by nanoCAGE (Supplementary Figure S3). We were not able to detect the expression of *Vmn1r201* in P50 female mice. As expected, we were not able to amplify any of the targets from liver RNA apart from Gapdh. We detected expression of *Olfr110*, *Olfr995*, and *Olfr1413* genes also in the VNO samples.

## Discussion

The traditional model of chemoreception in rodents considers MOE and VNO as two independent functional units, where sensing of odor mixtures and pheromones are segregated in independent detection systems (Mombaerts, 2004). In recent years, experimental evidences have suggested that these functional boundaries are uncertain since both structures can sense volatile plus non-volatile compounds and pheromonal plus non-pheromonal cues.

The activity of the VNO is not required for some olfactory-mediated instinctual behaviors (Dorries et al., 1997; Stowers and Logan, 2010). In turn, upon MOE selective ablation, male mice display a critical loss of interest toward female inspection and mounting, two behaviors classically attributed to the influence of pheromones (Yoon et al., 2005). Mouse models knocked out for the canonical mediators of OR signaling show impaired ability to fight and mate (Wang et al., 2006), unusual sexual behavior and lack of male-to-male aggressiveness (Wang and Storm, 2011). Neurons residing in MOE respond physiologically to compounds that have pheromonal characteristics leading to stereotyped behaviors including suckling, mate identification and male aggressiveness (Zufall and Leinders-Zufall, 2007; Stowers and Logan, 2010).

The existence of shared detection capabilities between VNO and MOE has been first suggested by the presence of 44 ORs in the VSNs; these ORs are canonically co-expressed with *Gαi2* and project their axons to the accessory olfactory bulb (Levai et al., 2006).

However, the molecular basis of pheromone sensing in MOE remains poorly understood although several evidences suggest that its receptors repertoire extends beyond ORs. A significant example is provided by the expression of several members of trace amine-associated receptors (*TAARs*) and guanylyl cyclase-D receptor (*GC-D*) in subsets of OSNs (Liberles and Buck, 2006; Zufall and Munger, 2010).

Intriguingly, two members of the murine V1Rd family, *V1rd17* and *V1rd20*, have been found expressed in MOE at embryonic and postnatal stages (Karunadasa et al., 2006). A custom microarray-based gene expression analysis has previously detected mRNAs for three V1Rs and two V1Rs, however their identity has not been disclosed (Zhang et al., 2010). A putative human pheromone receptor, *V1RL1*, has been identified by Southern blot analysis of RT-PCR products in human olfactory mucosa as well as in other human tissues (Rodriguez et al., 2000).

Both V1Rs and V2Rs are G protein-coupled receptors (GPCRs) but share little homology. The mouse genome contains 191 intact V1Rs genes with predicted short extracellular domain and no introns in the coding sequence (Zhang et al., 2010); conversely, the 123 intact V2Rs genes are structurally characterized by a predicted long and highly variable N-terminal and are encoded by multiple exons (Young and Trask, 2007; Zhang et al., 2010). The division between these two receptor families is also functional, since they are coupled to different α-subunits of the trimeric G protein (Gαi2 for VIRs and *Gαo* for V2Rs) and bind different sets of molecules (Berghard and Buck, 1996).

No evidence for the presence of additional V1Rs or of any V2Rs in MOE has been provided so far.

Here we have taken advantage of nanoCAGE (Plessy et al., 2010), a next-generation sequencing technology for unbiased 5′-end transcriptome profiling, to detect in the MOE consistent evidences of transcription that can be associated to a large number of V1Rs and V2Rs genes. NanoCAGE enables the quantitative measurement of transcripts expression level along with the precise definition of their TSSs from nanograms of total RNA obtained from fixed tissues. Being a single-nucleotide resolution technology, it greatly differs in terms of quantitative and qualitative output from microarray platforms or from PCR screenings based on degenerated oligonucleotides, as used so far to assess VRs expression in MOE. Furthermore, nanoCAGE was applied to RNA purified from tissue harvested by LCM (Plessy et al., 2012) to increase sensitivity for gene expression detection.

By multiple lines of evidence obtained from different experimental approaches we have proved that *Vmn1r201*, *Vmn2r26*, and *Vmn2r69* genes are expressed in the MOE by cells that reside in the basal and medial layers of the tissue and display morphological similarities with OSNs. Importantly, we have found that the absolute number of cells expressing these genes in the MOE of adult mice is comparable to what has been reported for *Vmn2r26* in the VNO (Del Punta et al., 2002; Leinders-Zufall et al., 2009). We have demonstrated by qRT-PCR that the expression levels of *Vmn2r26* and *Vmn2r69* in the MOE of sexually mature, adult mice are comparable to that of ORs with a similar total tags count in the nanoCAGE libraries.

Interestingly, *V2r1b* (*Vmn2r26* in the Refseq database) has been recently shown to respond to subpicomolar concentrations of MHC class I peptides in the VNO (Leinders-Zufall et al., 2009). Since the same class of non-volatile chemosignals is able to elicit physiological responses in the MOE (Spehr et al., 2006), our data suggests that *Vmn2r26* or additional highly homologous V2Rs may have a role in MHC class I peptides detection.

In the case of cells expressing *Vmn2r69*, we observed a spatial restriction in the dorsal turbinates of MOE suggesting the anatomical segregation of some V2Rs, as shown for several ORs. Interestingly, cells expressing *Vmn1r201* were much less abundant than those positive for *Vmn2r26* and *Vmn2r69* although consistently found in the MOE of all tested animals. It will be important to assess in details the identity of V1Rs- and V2Rs-expressing cells in the MOE by mapping their axonal projections.

NanoCAGE data have also revealed the expression of several V1Rs and V2Rs in addition to the ones we have validated. In some cases the low associated TPM scores suggest that a given VR may be expressed by a very restricted number of cells, as shown by the comparison of the DeepCAGE data with *in situ* hybridization for the hippocampus (Valen et al., 2009).

We have also detected relevant TSSs for several core components of the V1Rs- and V2Rs-associated chemo-transduction machinery including *Trpc2*, *Gαo*, and *Gαi2* that were then validated by RT-PCR from LCM-purified RNA. While they have been previously found expressed in the rodent MOE (Berghard and Buck, 1996), these observations have apparently attracted little attention.

These results strongly support the idea that transcripts from VRs loci are translated in signaling receptors. However, within the context of this work, we cannot exclude that some of V1Rs and V2Rs transcripts may not encode for VRs proteins but be non-coding RNA isoforms. A detailed analysis of transcript anatomy for every single gene will assess this important issue.

Interestingly, more than half of TCs upstream of V1Rs and V2Rs overlap with LINE1 transposable elements. The extraordinary content of LINE1 in V1Rs, V2Rs and ORs loci has been investigated for the first time by Kambere and Lane (2009). Focusing on V1Rs loci, the authors proposed an epigenetic role of LINE1 elements in the monoallelic expression of VR and OR genes on the basis of similar observations made on the inactivation mechanism of the X chromosome (Bailey et al., 2000).

Our analysis of MOE nanoCAGE data shows that LINE1s hosted in V1Rs and V2Rs loci are transcribed, thus adding a significant piece of information. Although we observe the transcription of members of active, young LINE1 families in both V1Rs and V2Rs loci (L1Md_A, L1Md_F, L1Md_F2, L1Md_F3, L1Md_Gf, L1Md_T) we do not confirm any preferential expression in comparison to ancestral, non-active ones (Supplementary Table S3). Several models can be proposed for how LINE1 elements can modulate the transcription of proximal VR genes. LINE1s present predominantly single-peak promoters that are active in somatic cells and exhibit far higher tissue specificity than conventional promoters, frequently driving transcription of nearby protein-coding genes. Along with the canonical 5′-sense promoter, LINE1s host a 5′-antisense promoter that in human cell lines is involved in the transcription of chimeric transcripts harboring partial sequences of sense and antisense downstream protein-coding mRNAs (Speek, 2001). An additional sense promoter is contained within the 3′ of LINE1s that may influence the local transcriptional activity of genes proximal to the insertion sites and even constitute alternative promoters for downstream sense protein-coding genes (Faulkner and Carninci, 2009; Faulkner et al., 2009). Alternatively, the presence of LINE1s may modulate nearby VR and OR genes expression through epigenetic mechanisms. In this context it is interesting that the GC-rich region of the LINE1 5′-sense promoter is a target for methylation and a potential trigger for seeding and spreading of heterochromatin (Zhang et al., 2012). The demethylation of this region can drive transcription of LINE1s and induce functional chromatin domains that may inhibit the influence of repressive chromatin modifications, a mechanism already described for the mouse growth hormone locus (Lunyak et al., 2007). In addition, small non-coding RNAs transcribed from LINE1s and other retrotransposons may also be involved in the regulation of local chromatin structure (Olovnikov et al., 2012).

Considering the sustained neurogenic capabilities of MOE and VNO throughout the lifespan of rodents (Dulac and Zakhary, 2004; Brann and Firestein, 2010) and the documented activation of LINE1s during adult neurogenesis (Muotri et al., 2005; Kuwabara et al., 2009), transcriptional activation of LINE1s in VRs and ORs loci may be involved in the neurogenesis and/or maturation of OSNs and VSNs. The 5′-UTR, ORF1, and ORF2 regions of mouse, rat and human LINE1s share conserved binding sites for Sox2/LEF which act as bi-directional promoters once transcribed during neurogenesis and can induce the transcriptional activation of proximal neuronal genes. Intriguingly, our nanoCAGE libraries show a pronounced expression of Sox2 in the MOE.

In summary, the application of next generation sequencing coupled to LCM-based tissue sampling is a powerful strategy to unveil unexpected transcription of protein coding genes and repetitive elements. While we and others are observing ORs expression outside of sites involved in olfactory chemoreception (Kang and Koo, 2012; Flegel et al., 2013; Foster et al., 2013; Li et al., 2013), evidences for VRs transcription in the central nervous system have been reported while drafting this manuscript (Ansoleaga et al., 2013; Garcia-Esparcia et al., 2013). Once again, these discoveries strongly suggest that our definition of “ectopic” expression needs to be revised and that a better understanding of its biological meaning is required for each case. V1Rs and V2Rs transcription in both chemosensory organs supports the possibility of a functional cross-talk between MOE and VNO posing interesting questions about their relative contribution to pheromone-triggered social behaviors in rodents.

## Authors contributions

Giovanni Pascarella, Charles Plessy, Dejan Lazarevic, Christina Vlachouli, Roberto Simone, Silvia Zucchelli performed the experiments, Charles Plessy, Jun Kawai, Carsten O. Daub, Yoshihide Hayashizaki, and Piero Carninci were involved in the large-scale data production, Charles Plessy, Nicolas Bertin, Altuna Akalin, Geoffrey J. Faulkner, Boris Lenhard analyzed the data, Giovanni Pascarella, Charles Plessy, Stefano Gustincich, and Piero Carninci wrote the manuscript, Stefano Gustincich and Piero Carninci designed the experiments and directed the project.

### Conflict of interest statement

The authors declare that the research was conducted in the absence of any commercial or financial relationships that could be construed as a potential conflict of interest.
