# Supplementary material for: NanoCAGE analysis of the mouse olfactory epithelium identifies the expression of vomeronasal receptors and of proximal LINE elements
Source: Front Cell Neurosci. 2014 Feb 18;8:41. doi: 10.3389/fncel.2014.00041 (PMC3927265; doi:10.3389/fncel.2014.00041)
Supplement: Supplementary file 1 [file Presentation1.PDF]

Supplementary Material

NanoCAGE analysis of the mouse olfactory epithelium identifies the expression of vomeronasal receptors and of proximal LINE elements.

Giovanni Pascarella^{1, 2}, Dejan Lazarevic^{1, 3}, Charles Plessy², Nicolas Bertin², Altuna Akalin⁴, Christina Vlachouli¹, Roberto Simone¹, Geoffrey J. Faulkner⁵, Silvia Zucchelli^{1, 6}, Jun Kawai², Carsten O. Daub², Yoshihide Hayashizaki², Boris Lenhard^{4*}, Piero Carninci^{2*} and Stefano Gustincich^{1*}.

¹ Sector of Neurobiology, International School for Advanced Studies (SISSA), Via Bonomea 265, 34136 Trieste, Italy

² RIKEN Yokohama Institute, Center for Life Science Technologies, 1-7-22 Suehiro-chô, Tsurumi-ku, Yokohama, Kanagawa 230-0045 Japan

³ CBM, AREA Science Park, s.s. 14, Km 163.5, Basovizza, 34012 Trieste, Italy

⁴ University of Bergen, Bergen Center for Computational Science - Computational Biology Unit and Sars Centre for Marine Molecular Biology, Hoyteknologisenteret, Thormohlensgate 55, N-5008 Bergen, Norway.

⁵ Cancer Biology Program, Mater Medical Research Institute, South Brisbane, Australia; School of Biomedical Sciences, University of Queensland, Brisbane, Australia.

⁶ Department of Health Sciences, University of Eastern Piedmont “A. Avogadro”, via Solaroli 17, 28100 Novara, Italy

Correspondence:

Corresponding Authors:

Boris Lenhard

University of Bergen, Bergen Center for Computational Science - Computational Biology Unit and Sars Centre for Marine Molecular Biology, Hoyteknologisenteret, Thormohlensgate 55, N-5008 Bergen, Norway. boris.lenhard@bccs.uib.no

Piero Carninci

RIKEN Yokohama Institute, Center for Life Science Technologies, 1-7-22 Suehiro-chô, Tsurumi-ku, Yokohama, Kanagawa 230-0045 Japan. carninci@riken.jp

Stefano Gustincich

Area of Neurobiology, International School for Advanced Studies (SISSA), Via Bonomea 265, 34136 Trieste, Italy. gustinci@sissa.it

1. Supplementary Data

1.1 URLs of NanoCAGE tracks uploadable in the UCSC Genome Browser.

<http://plessy-20090306:sRehJ6RY@134.160.84.27/plessy-20090306/Plessy-2009.wig.bz2>

Accession Number for deposited Raw sequences: SRP000696

1.2 Primers used for validation of nanoCAGE data.

1.2.1 Primers used to validate the expression of V1Rs, V2Rs and components of the pheromones vomeronasal transduction pathway.

Gene name	Sequences (5'→3')	Expected PCR product size (bp)
Vmn2r3	Fw: AGACTGAGGCAATTGTGAATC<br>Rev: TCATATTCACCTCTGTGATCCAC	549bp
Vmn2r26	Fw: TGCTTTCTCTCCCCTAGTTG<br>Rev: ATAGCTGATCTGTGGGACGT	511bp
Vmn2r29	Fw: CAGGAAGTAGAAAGATGCCATCCTC<br>Rev: GAAACAGCCACAGTGAATACAATTCC	484bp
Vmn2r69	Fw: CAGAATTCCTGAGCTTTACTGTGGT<br>Rev: AGGTATCTCAGGTTGCTGGTGT	508bp
Vmn2r76	Fw: GAATTCCTCTTCAACTCCTTTCA<br>Rev: GTAGAGTCTGTCTAACATCTGTG	604bp
Vmn2r95	Fw: CTTTCCATGGCAGCTTAATACTTTCC<br>Rev: GAAGTTGCCATCCATATTCCACAAAG	858bp
Gnao1	Fw: GCAACCTATTTGACTGCTTCATGG<br>Rev: CACTGCCTGGTGGTATATGAGG	534bp
Gnai2	Fw: CTTACACTTCAAGATGTTTGATGTG<br>Rev: GTCCTTCAGGTTGTTCTTGAT	472bp
Trpc2	Fw: CCAGAAGATCGAGGATGATGCTG<br>Rev: CAATCCCAGGCATAGTCAGCT	569bp
Vmn1r50	Fw: TTCCATCTGAACAGGCTTTCTCTG<br>Rev: TCCTCACTTCCAGAAACGTTATAGAC	576bp
Vmn1r51	Fw: ATTCTCTGAAGTGAGTGTTGGGATC<br>Rev: CCTCATGGTCATCATTGTGGAAAGTA	526bp
Vmn1r10	Fw: TTCAATTTGGCCTTCAGTACTAACC<br>Rev: GGGTAATTGTGGGATAGGCATACA	433bp
Vmn1r201	Fw: CCTCTCGACCTCATTCTCAT<br>Rev: AGGTAGAGGACACGTTTGT	527bp
Omp	Fw: AGCTAGCAACAGTGATGTCCCTG<br>Rev: CGGATCCGAGTGAGGCAGAGTTG	655bp

1.2.2 Primers used to validate the TSSs identified by NanoCAGE of selected V2Rs.

Gene name	Sequences (5'→3')
Vmn1r228	Fw: ATCCACTTGAAGTGAAGTTTACA<br>Rev: GAGCATTAAAGAATAGTGATACTCC
Vmn2r3	Fw: ACACACTCTAACACTCTATTAAGG<br>Rev: AAACAGTTGTCAAATATCTGGT
Vmn2r69	Fw: GAACAAAGGTATTACCACTTATTTGT<br>Rev: ACCTTCTTGTCTATATTCCAAGT
Vmn2r76	Fw: GCATAATTCATACATGGCATTCTAT<br>Rev: GGAAAATAATCACCTCTTCTTGAG

1.3 PCR products obtained for TCs validation:

Vmn1r228

TCTGCTGAAAAGAGCGAGGTGTGCAATTTGTACTGAATCTGATCTGCCATGGAAGATGC
AACTTTGGATACAATTTCCCTGAGGGTGTTCAGCTGCCTCAACAAGAAAAGTATTCCAG
AGATCGTCACTGTGTATCACCTGGGGCAGTGAAGTTGCAATGGTCAAGTGGGGACGTCT
CTTATTAGCAGTGGCTACTTGTTTATACTTTGTGAAATTTGGTTTTGAAGAAATCATCAC
AACAGTGAGATTTTTGACAAATATGTCCATAAGTCTTCTTGATAAATATTTTCAGTCATGT
GAAAGCTATCAACAGTTCTTGATACCTACATTATCCTAATCCTTAATAAGAGCTGCTATC
TCCAATGTTTTTTTTTCATTACATCTATGCTACTGTATAATGACACCAAACAGTGAATCTA
ATAAGAACACTGGGCATTTTTTCCTTGTTTAATCATCATTTCAATATGATTTTGTGTTTTT
TTTCTTATATACAATGTCCTGTAGCTTTGAATAATCTATTTTCTTTGTATTTTCACTTGGA
AAGAGTTCACCACACAAGGTAATTTTGCTGTGACTCCCTCCTACAAGTGAAGCAT
ACATATGGCATCACATATGAGTTTTAGTGAAATTTGATTAAAGCTCTTTTCACTGTAAC
ATGTTCTAATTGTTAGCATCGACTTACACTTAGTATTCTATTGCTTACTATATACATTTTT
CTACAATTATATATTTCACTGCTGTGTATAAAACATATGGTTTATAGCTTCAGTGTCACAT
GAGGGCCATATGAATTTTTTCATGTTCAAAATGGTGACTAAATTCTATTTTCTTCTATATG
ACTGGATTGAAGAAGAAGTGAGTATTCA TTCCATCTAAACTCATAGCATTTCCTT

Vmn2r3

ACACACTCTAACACTCTATTAAGGTGCTTCCTTAAAACCTGTGTCACTTCAGCCCTGTAA
TTACTCTCAATCTCACCACATGAAAATGGCTATCAGAAAGAAGTGCTTGATTCTGGAATT
AACTGTATTTTTGTGTGTTGAATTATATGCTCAATATGAAGACCAGAATATGACTTGCAG
GTTGATGCGCAAATTTAATTTGACTGGATATGTAGAAGCTGAAAATCATCATTTTGTAT
TGGAGGACAGTTTCCTGTTCACTATAGGACCATCCCAACAAGTGAAGTCTGATGAGGAAC
CAGAATCTCCCATGTGTGAGGGGTTTAATTTCCGGGGTTTCCGCTGGATGAAAACCATG
ATCCACACCATCAAGGAGATTAATGAGAGGAAGGATATTTTGCCCGAACACACTCTGGG
CTACCAGATATTTGACAACTGTTT

72 **Vmn2r69**

73 GAACAAAGGTATTACCACTTATTTGTCACCTGAACATATGAACTCAGATGCTAAGCCTA
 74 AGGACATAGATTGCTCAAGAATCCTCCAGAGGCAAACGTAGAACTGAAAAGTAAATTA
 75 CTAAGAGAAGTACATTTGTCCTTTAGTAGTGAGTACAGTAATAAATAGTGAGTTCTGGA
 76 TGTGTTACACAATAATAATGCATGAGATTCTGTATATCTGACTGTATATGCTACATTTT
 77 GCTCTGGCATATATTTTGTGGACTGGAAAGAGATGTTCTCTTTGATCTCTGTCATCTGGTT
 78 TCTGAAGATTAACTTATGTTTTGTCATTTGAGTGACCCCAGTTGCTTTTGGAGAATAAA
 79 AGAACTGAAAATGATCTAGGAGATACAGAACTTATTGTTTGTCTTCTATTTCACAAA
 80 GCAAGGTTATGTGAAGAATGATTATTTTCAGTTGGAATATAGACAAGAAGGT

81 **Vmn2r76**

82 GCATAATTCATACATGGCATTCTATCTGATGCTTCATGTTAACTTCTGCTCAGGCTCAT
 83 AGCTTGCATGACATGATAGACATGTTCTCCTTCATTTCTGTCTTCTTGGTCTTAAAGGCTT
 84 CCATGCTTTTCTGCTCTTTGAATGAACCCAAGTGCTTTTTTGAAGAATAAAAGACAAGAAAA
 85 ACAAGGATGGCGATAAGGAAGTTGATTGCTTTTTTTCCATTTATACTAAGCATGGACATA
 86 TGAAGAATGAACATTTTAGTGGAATCTAGACAAGCAGTGACGACTGTTCTGATTATC
 87 CTAGTCAAATGGATACACTGATGGATTGCACACATAAATCAACCTGCTTTGTAAGAGAT
 88 TGACAGGTTGACAATAAGAACATCCACTTGATTTTATCACTTTATTTTGCTATTGAAGA
 89 AATCAATATGAGCCCCCATCTATTACCTAATATTTCTCTATTAGTTAAAGTTGAGTGTA
 90 GTTATTGGCTGATGGGAGTAAAGTCAGTTTATCCTCAAGAAGAGGTGATTATTTTCC

91 **1.4 *In situ* hybridization protocol.**

92 Slides were incubated in 4% PFA/PBS1x at RT for 10 minutes and washed in PBS1x two times for 5
 93 minutes. After incubation in a 1%-3% H₂O₂ /PBS1x solution at RT for 30 minutes to block
 94 endogenous peroxidases, the slides were put at 37°C in a 0.1µg/ml Proteinase K solution prepared in
 95 TE buffer (1M Tris-Cl pH8.0 / 0.5M EDTA pH8.0) for 10 minutes. A second incubation on 4%
 96 PFA/PBS1x for 10 minutes at RT was followed by two PBS1x washes for 5 minutes each. The slides
 97 were then treated with 0.2M HCl at RT for 10 minutes, washed again in PBS1x and pre- incubated
 98 for 1 minute in 0.1M Triethanol amine-HCl pH8.0 (TEA buffer). After the pre- incubation, acetic
 99 anhydrate was slowly added to the TEA solution and incubated at RT for 5 minutes while gently
 100 stirring. The slides were washed in PBS1x for 5 minutes and air-dried for 30- 180 minutes. 200-
 101 600ng of RNA probes were added to 150µl of hybridization mix pre-heated at 85°C for 5 minutes
 102 and left at the same temperature for additional 3 minutes. The composition of the hybridization mix
 103 was 1x salts solution (0.3M NaCl, 0.01M Tris-Cl pH8.0, 0.01M NaH₂PO₄, 0.005M NaEDTA pH8.0,
 104 0.2% Ficoll, 0.2% Polyvinyl pyrrolidone), 0.05M DTT, 0.1mg/ml yeast tRNA (Sigma-Aldrich), 10%
 105 Dextrane sulphate, 50% Formamide and 0.5mg/ml Polyadenilic acid (Sigma-Aldrich). The
 106 appropriate mix was pipetted on the sections, the slides were covered with Parafilm (Alcan
 107 Packaging, WI, USA) and put at 58°C-60°C in humid chambers containing 50% formamide. After
 108 14-16 hours the parafilm covers were gently removed and the slides were washed with 5x SSC for 5
 109 minutes at 65°C, moved in 2x SSC/50% formamide for 30 minutes at 65°C, then washed with TNE
 110 (10mMTris-Cl pH 7.5, 500mM NaCl, 1mM EDTA pH8.0) for 10 minutes at 37°C, incubated with
 111 0.2mg/ml RNase A solution (Sigma) at 37°C for 30 minutes and again with TNE for 10 minutes at
 112 37°C. Slides were then washed with 2x SSC for 20 minutes at 65°C, 0.2x SSC for 20 minutes at
 113 65°C and with 0.1x SSC for 20 minutes at 65°C. Sections were circled with Super PapPen and
 114 processed for detection.

1.5 Detection of biotin-labelled RNA probes.

Slides were blocked with 0.5% Blocking Reagent (Roche) in 0.1M Tris-Cl pH7.5, 0.15M NaCl for 1 hour at RT and incubated with HRP-conjugated streptavidin (1:2000; Perkin Elmer Life Sciences). After washing in TNT buffer (0.1M Tris-Cl pH7.5, 0.15M NaCl, 0.05% Tween20) the sections were reacted for 5 to 10 minutes with Cy3-tyramide (Perkin Elmer) dissolved in the proper buffer, washed 3x with TNT and mounted with Vectashield (Vector Laboratories, Burlingame, CA).

2. Supplementary Tables and Figures.

Supplementary Table S1. The list of all nanoCAGE Tag Clusters (TCs) detected by nanoCAGE defining transcription starting sites (TSSs) in V1Rs and V2Rs genes loci along with a detailed qualitative description of the mapping for each TC.

Supplementary Table S2. The list of all V1Rs and V2Rs genes having a TCs mapped 5' upstream of the Refseq annotation and a total tag count for genes involved in the ORs and VRs signal transduction pathways.

Supplementary Table S3. Classification of all LINE1 elements associated with V1Rs and V2Rs

Supplementary Figure S1. *in situ* hybridization for *Vmn2r26* and *Vmn2r69* on the dorsal/central turbinates where the highest densities of positive cells were detected.

Supplementary Figure S2. Representative images of negative controls for the ISH experiments on VNO and MOE sections.

Supplementary Figure S3. qRT-PCR data showing the expression levels of the selected targets in the VNO of all tested mice and the copy number calculation.

2.1   Supplementary Tables.

Supplementary Table S1 (next page).

List of all TCs associated with downstream V1Rs and V2Rs genes. For TCs mapping in conserved blocks, the column “cons.clu.new” is the conserved block cluster overlap, with the indication of “0” for no overlap and “1” for evidence of overlap.

name	chrom	strand	start.lbase	end	exp.tpm.OE_S	distance to annotated TSS (bp)	refseqID	gene_symbol	repeat overlap	rat	human	horse	dog	opposm	platps	cons.clu.new	repeat Class
chr13:221567 chr13		+	22156795	22156795	0,02415145	22951	NM_145850	V1rh17	1	0	1	0	0	0	0	0	ow_complexity
chr13:221724 chr13		+	22172429	22172429	0,02415145	7317	NM_145850	V1rh17	1	0	0	0	0	0	0	1	SINE
chr13:222192 chr13		-	22219245	22219245	0,09660578	24710	NM_145844	V1rh2	0	1	0	0	0	0	1	0	none
chr13:222828 chr13		-	22282838	22282838	0,02415145	33703	NM_145844	V1rh2	0	1	1	0	0	0	0	0	none
chr13:222673 chr13		-	22267322	22267322	0,02415145	29927	NM_145846	V1ri8	0	1	1	0	0	1	1	0	none
chr13:222691 chr13		-	22269110	22269112	0,04830289	31715	NM_145846	V1ri8	0	0	0	0	0	0	0	0	none
chr13:223480 chr13		+	22348046	22348046	0,02415145	22184	NM_134223	V1ri6	1	0	0	0	0	0	0	0	LINE
chr13:223744 chr13		+	22374460	22374461	0,28981734	45319	NM_134244	V1rh21	0	0	0	0	0	1	0	0	none
chr13:223745 chr13		+	22374509	22374514	0,21736301	45265	NM_134244	V1rh21	0	0	0	0	0	1	0	0	none
chr13:223745 chr13		+	22374541	22374541	0,07245434	45238	NM_134244	V1rh21	0	0	0	0	0	1	0	0	none
chr13:223745 chr13		+	22374562	22374562	0,01449087	45217	NM_134244	V1rh21	0	0	0	0	0	1	0	0	none
chr13:223754 chr13		+	22375454	22375454	0,02415145	44325	NM_134244	V1rh21	0	0	0	0	0	1	0	0	none
chr13:223758 chr13		+	22375852	22375852	0,04830289	43927	NM_134244	V1rh21	0	0	0	0	0	1	0	0	none
chr13:224104 chr13		+	22410417	22410417	0,14490867	9362	NM_134244	V1rh21	0	0	0	0	0	0	0	0	none
chr13:224128 chr13		+	22412840	22412840	0,02415145	6939	NM_134244	V1rh21	1	0	0	0	0	0	0	0	SINE
chr13:224143 chr13		+	22414387	22414387	0,02415145	5392	NM_134244	V1rh21	1	0	0	0	0	0	0	0	LINE
chr13:224171 chr13		+	22417194	22417194	0,02415145	2585	NM_134244	V1rh21	0	0	1	0	0	1	1	1	none
chr13:224268 chr13		+	22426815	22426815	0,02415145	19399	NM_134220	V1ri3	1	0	0	0	0	0	0	0	LINE
chr13:224280 chr13		+	22428023	22428023	0,02415145	18191	NM_134220	V1ri3	0	0	0	0	0	1	0	0	none
chr13:224443 chr13		+	22444340	22444340	0,12075723	1874	NM_134220	V1ri3	0	1	1	0	0	0	1	1	none
chr13:224642 chr13		+	22464263	22464263	0,24151445	10143	NM_134213	V1rh4	1	0	0	0	0	0	0	0	LINE
chr13:224678 chr13		+	22467838	22467838	0,02415145	6568	NM_134213	V1rh4	1	0	0	0	0	0	0	0	Other
chr13:224695 chr13		+	22469569	22469569	0,02415145	4837	NM_134213	V1rh4	1	0	0	0	0	0	0	0	LTR
chr13:224733 chr13		+	22473373	22473373	0,02415145	1033	NM_134213	V1rh4	0	0	1	0	0	1	1	1	none
chr13:224863 chr13		+	22486359	22486359	0,02415145	538	NM_134212	V1rh3	1	0	0	0	0	1	1	1	LINE
chr13:224888 chr13		+	22488813	22488813	0,04830289	77673	NM_134221	V1ri4	0	0	1	0	0	0	0	0	none
chr13:224954 chr13		+	22495447	22495447	0,16906012	71039	NM_134221	V1ri4	1	0	0	0	0	0	0	0	LTR
chr13:225056 chr13		+	22505633	22505633	0,02415145	60853	NM_134221	V1ri4	0	0	0	0	0	0	0	0	none
chr13:225092 chr13		+	22509213	22509213	0,02415145	57273	NM_134221	V1ri4	1	0	0	0	0	0	0	0	LTR
chr13:225177 chr13		+	22517741	22517741	0,19321156	48745	NM_134221	V1ri4	1	0	0	0	0	0	0	0	LINE
chr13:225222 chr13		+	22522218	22522218	0,02415145	44268	NM_134221	V1ri4	1	0	0	0	0	0	0	0	LINE
chr13:225352 chr13		+	22535271	22535271	0,02415145	31215	NM_134221	V1ri4	1	0	1	0	0	1	1	0	SINE
chr13:225406 chr13		+	22540645	22540645	0,02415145	25841	NM_134221	V1ri4	1	0	0	0	0	0	0	0	LINE
chr13:225441 chr13		+	22544133	22544133	0,02415145	22353	NM_134221	V1ri4	1	0	0	0	0	0	0	0	LINE
chr13:225483 chr13		+	22548300	22548300	0,02415145	18186	NM_134221	V1ri4	1	0	0	0	0	0	0	0	SINE
chr13:225497 chr13		+	22549702	22549702	0,04830289	16784	NM_134221	V1ri4	1	0	0	0	0	0	0	0	LINE
chr13:225589 chr13		+	22558944	22558944	0,14490867	7542	NM_134221	V1ri4	0	0	0	0	0	1	1	1	none
chr13:226004 chr13		-	22600447	22600447	0,38642312	6332	NM_134224	V1ri7	1	0	0	0	0	0	0	0	LINE
chr13:226052 chr13		-	22605241	22605241	0,02415145	11126	NM_134224	V1ri7	0	0	0	0	0	0	0	0	LINE
chr13:226067 chr13		+	22606767	22606767	0,02415145	9152	NM_134236	V1rh11	0	0	0	0	0	0	0	0	none
chr13:226883 chr13		-	22688330	22688330	0,02415145	3530	NM_134217	V1rh8	1	0	0	0	0	0	0	0	LINE
chr13:227090 chr13		-	22709074	22709074	0,12075723	24274	NM_134217	V1rh8	1	0	0	0	0	0	0	0	LINE
chr13:227213 chr13		-	22721347	22721347	0,02415145	8442	NM_134216	V1rh7	0	0	1	0	0	1	0	1	none
chr13:227253 chr13		-	22725359	22725359	0,02415145	12454	NM_134216	V1rh7	1	0	0	0	0	0	0	0	LINE
chr13:227426 chr13		-	22742636	22742636	0,02415145	29731	NM_134216	V1rh7	0	0	1	0	0	0	0	0	none
chr13:227546 chr13		-	22754672	22754672	0,02415145	41767	NM_134216	V1rh7	1	0	0	0	0	0	0	0	LINE
chr13:227717 chr13		-	22771737	22771737	0,19321156	58832	NM_134216	V1rh7	1	0	0	0	0	0	0	0	LTR
chr13:227752 chr13		-	22775218	22775220	0,14490867	62315	NM_134216	V1rh7	1	0	0	0	0	0	0	0	LTR
chr13:228277 chr13		-	22827736	22827736	0,09660578	114831	NM_134216	V1rh7	0	0	1	0	0	1	0	0	none
chr13:228489 chr13		-	22848940	22848940	0,02415145	136035	NM_134216	V1rh7	1	0	0	0	0	0	0	0	LINE
chr13:228790 chr13		-	22879036	22879036	0,02415145	13841	NM_134218	V1rh9	1	0	0	0	0	0	0	0	SINE
chr13:228970 chr13		-	22897074	22897074	0,14490867	31879	NM_134218	V1rh9	1	0	0	0	0	0	0	0	SINE
chr13:229384 chr13		-	22938403	22938403	0,04830289	18419	NM_134235	V1rh10	1	0	0	0	0	0	0	0	LTR
chr13:229388 chr13		-	22938878	22938878	0,04830289	18894	NM_134235	V1rh10	1	0	0	0	0	0	0	0	LTR
chr13:229404 chr13		-	22940488	22940488	0,2656659	20504	NM_134235	V1rh10	0	0	0	0	0	0	0	0	none
chr13:229583 chr13		-	22958334	22958334	0,09660578	13969	NM_134243	V1rh20	0	0	0	0	0	0	0	0	none
chr13:229716 chr13		-	22971667	22971667	0,02415145	27302	NM_134243	V1rh20	1	0	0	0	0	0	0	0	LINE
chr13:229722 chr13		-	22972269	22972269	0,02415145	27904	NM_134243	V1rh20	1	0	0	0	0	0	0	0	LTR
chr13:229833 chr13		-	22983393	22983393	0,02415145	7362	NM_134241	V1rh18	0	0	0	0	0	0	0	0	none
chr13:230760 chr13		+	23076016	23076016	0,02415145	27101	NM_134215	V1rh6	0	0	0	0	0	1	0	0	none
chr13:230776 chr13		-	23077601	23077601	0,07245434	101570	NM_134241	V1rh18	1	0	0	0	0	0	0	0	LTR
chr13:230788 chr13		-	23078874	23078874	0,02415145	102843	NM_134241	V1rh18	1	0	0	0	0	0	0	0	Simple_repeat
chr13:230862 chr13		+	23086210	23086210	0,02415145	16907	NM_134215	V1rh6	1	0	0	0	0	0	0	0	LINE
chr13:230906 chr13		+	23090685	23090685	0,02415145	12432	NM_134215	V1rh6	1	0	0	0	0	0	0	0	LTR
chr13:230970 chr13		+	23097015	23097015	0,02415145	6102	NM_134215	V1rh6	1	0	0	0	0	0	0	0	LINE
chr13:231019 chr13		-	23101948	23101948	0,02415145	125917	NM_134241	V1rh18	0	0	1	0	0	1	0	0	none
chr13:231173 chr13		+	23117397	23117397	0,02415145	8809	NM_134214	V1rh5	1	0	0	0	0	0	0	0	LINE
chr13:231574 chr13		+	23157489	23157489	0,14490867	10171	NM_134219	V1ri2	1	0	0	0	0	0	0	0	LTR
chr13:231790 chr13		+	23179069	23179069	0,02415145	11948	NM_134245	V1ri10	0	0	0	0	0	0	0	0	none
chr13:232068 chr13		-	23206806	23206809	0,16906012	209	NM_134239	V1rh14	0	0	1	0	0	0	1	1	none
chr13:232201 chr13		+	23220164	23220164	0,02415145	8189	NM_134222	V1ri5	1	0	0	0	0	0	0	0	LINE

chr13:232202 chr13	-	23220272	23220272	0,02415145	13672	NM_134239	V1rh14	1	0	0	0	0	0	0	0	0	0	LINE
chr13:232278 chr13	-	23227880	23227880	0,12075723	21280	NM_134239	V1rh14	0	1	1	0	0	0	0	0	0	0	none
chr13:232389 chr13	+	23238997	23238997	0,12075723	15514	NM_134238	V1rh13	1	0	0	0	0	0	0	0	0	0	LTR
chr13:232450 chr13	+	23245090	23245090	0,12075723	9421	NM_134238	V1rh13	0	0	0	0	0	0	1	0	0	1	none
chr13:232472 chr13	+	23247249	23247249	0,12075723	7262	NM_134238	V1rh13	0	1	0	0	0	0	0	0	0	1	none
chr13:232790 chr13	-	23279022	23279022	0,02415145	2628	NM_134237	V1rh12	0	0	1	0	0	0	0	0	0	1	none
chr13:232800 chr13	-	23280069	23280069	0,04830289	3675	NM_134237	V1rh12	0	0	1	0	0	0	0	0	0	1	none
chr13:233133 chr13	-	23313320	23313322	0,04830289	36926	NM_134237	V1rh12	0	0	0	0	0	0	0	0	0	0	none
chr13:233179 chr13	-	23317999	23317999	0,02415145	41605	NM_134237	V1rh12	0	0	0	0	0	0	0	0	0	0	none
chr17:206223 chr17	+	20622394	20622394	0,02415145	16868	NM_134194	V1re5	0	0	0	0	0	0	0	0	0	0	none
chr17:206299 chr17	+	20629977	20629977	0,02415145	9285	NM_134194	V1re5	1	0	0	0	0	0	0	0	0	0	LINE
chr17:206329 chr17	+	20632956	20632956	0,12075723	6306	NM_134194	V1re5	1	0	0	0	0	0	0	0	0	0	LINE
chr17:207471 chr17	+	20747181	20747181	0,16906012	77290	NM_134191	V1re2	1	0	0	0	0	0	0	0	0	0	LINE
chr17:207503 chr17	+	20750370	20750370	0,02415145	74101	NM_134191	V1re2	1	0	0	0	0	0	0	0	0	0	LTR
chr17:207520 chr17	+	20752050	20752050	0,02415145	72421	NM_134191	V1re2	1	0	0	0	0	0	0	0	0	0	LINE
chr17:207538 chr17	+	20753870	20753870	0,09660578	70601	NM_134191	V1re2	1	0	0	0	0	0	0	0	0	0	LINE
chr17:207653 chr17	+	20765364	20765378	0,07245434	59107	NM_134191	V1re2	1	0	0	0	0	0	0	0	0	0	LINE
chr17:207751 chr17	+	20775173	20775173	0,07245434	49298	NM_134191	V1re2	1	1	0	0	0	0	0	0	0	0	LINE
chr17:207831 chr17	+	20783144	20783145	0,03018931	41327	NM_134191	V1re2	0	1	0	0	0	0	0	0	0	0	none
chr17:207863 chr17	+	20786325	20786325	0,09660578	38146	NM_134191	V1re2	0	1	0	0	0	0	0	0	0	0	none
chr17:207985 chr17	+	20798589	20798639	5,80253693	25876	NM_134191	V1re2	0	0	0	0	0	0	1	0	0	0	none
chr17:207986 chr17	+	20798662	20798677	0,61317618	25802	NM_134191	V1re2	0	0	0	0	0	0	1	0	0	0	none
chr17:207987 chr17	+	20798704	20798733	3,1358947	25739	NM_134191	V1re2	0	0	0	0	0	0	1	0	0	0	none
chr17:207987 chr17	+	20798773	20798792	3,48354344	25682	NM_134191	V1re2	0	0	0	0	0	0	1	0	0	0	none
chr17:207988 chr17	+	20798849	20798915	2,11542905	25605	NM_134191	V1re2	0	0	0	0	0	0	1	0	0	0	none
chr17:207989 chr17	+	20798945	20798996	10,0544647	25510	NM_134191	V1re2	0	0	0	0	0	0	1	0	0	0	none
chr17:207991 chr17	+	20799150	20799150	0,02415145	25321	NM_134191	V1re2	1	0	0	0	0	0	1	0	0	0	Satellite
chr17:208016 chr17	+	20801671	20801671	0,02415145	22800	NM_134191	V1re2	1	0	0	0	0	0	1	0	0	0	LINE
chr17:208017 chr17	+	20801780	20801780	0,07245434	22691	NM_134191	V1re2	1	0	0	0	0	0	1	0	0	0	LINE
chr17:208057 chr17	+	20805716	20805716	0,02415145	18755	NM_134191	V1re2	1	0	0	0	0	0	1	0	0	0	LINE
chr17:208121 chr17	+	20812118	20812118	0,02415145	12353	NM_134191	V1re2	1	0	0	0	0	0	0	0	0	0	LINE
chr17:208135 chr17	+	20813519	20813519	0,02415145	10952	NM_134191	V1re2	1	0	0	0	0	0	0	0	0	0	LINE
chr17:208156 chr17	+	20815667	20815667	0,16906012	8804	NM_134191	V1re2	1	0	0	0	0	0	0	0	0	0	LINE
chr17:208167 chr17	+	20816770	20816770	0,02415145	7701	NM_134191	V1re2	1	0	0	0	0	0	0	0	0	0	LINE
chr17:208169 chr17	+	20816956	20816956	0,02415145	7515	NM_134191	V1re2	0	0	0	0	0	0	0	0	0	0	none
chr17:208199 chr17	+	20819955	20819955	0,12075723	4516	NM_134191	V1re2	0	1	0	0	0	0	1	0	0	1	none
chr17:208208 chr17	+	20820863	20820863	0,28981734	3608	NM_134191	V1re2	0	1	0	0	0	0	0	0	0	1	none
chr17:208296 chr17	+	20829697	20829697	0,02415145	42366	NM_134195	V1re6	0	1	0	0	0	0	0	0	0	0	none
chr17:208338 chr17	+	20833838	20833838	0,16906012	38225	NM_134195	V1re6	1	0	0	0	0	0	0	0	0	0	LINE
chr17:208353 chr17	+	20835368	20835368	0,02415145	36695	NM_134195	V1re6	1	0	0	0	0	0	0	0	0	0	LINE
chr17:208603 chr17	+	20860359	20860359	0,04830289	11704	NM_134195	V1re6	1	0	0	0	0	0	0	0	0	0	LINE
chr17:208608 chr17	+	20860826	20860826	0,02415145	11237	NM_134195	V1re6	0	1	0	0	0	0	0	0	0	1	none
chr17:208634 chr17	+	20863429	20863429	0,02415145	8634	NM_134195	V1re6	1	0	0	0	0	0	0	0	0	1	LINE
chr17:208669 chr17	+	20866917	20866921	0,19321156	5146	NM_134195	V1re6	0	1	0	0	0	0	0	0	0	1	none
chr17:209227 chr17	-	20922799	20922802	0,38642312	8580	NM_134192	V1re3	0	1	0	0	0	0	0	0	0	1	none
chr17:209251 chr17	-	20925110	20925112	0,16906012	10893	NM_134192	V1re3	1	0	0	0	0	0	0	0	0	0	LINE
chr17:209321 chr17	-	20932157	20932157	0,09660578	17938	NM_134192	V1re3	1	0	0	0	0	0	0	0	0	0	LINE
chr17:209451 chr17	+	20945132	20945132	0,07245434	6326	NM_134190	V1re1	1	0	0	0	0	0	0	0	0	0	LTR
chr17:209482 chr17	-	20948227	20948227	0,02415145	34008	NM_134192	V1re3	0	1	0	0	0	0	0	0	0	0	none
chr17:209494 chr17	-	20949479	20949479	0,02415145	35260	NM_134192	V1re3	1	1	0	0	0	0	1	0	0	0	LINE
chr17:209569 chr17	+	20956947	20956949	0,07245434	26567	NM_134197	V1re8	0	0	0	0	0	0	0	0	0	0	LINE
chr17:209769 chr17	+	20976975	20976975	0,02415145	6539	NM_134197	V1re8	0	1	0	0	0	0	0	0	0	1	none
chr17:210351 chr17	-	21035122	21035122	0,16906012	7506	NM_134196	V1re7	0	1	0	0	0	0	0	0	0	1	none
chr17:210400 chr17	-	21040041	21040041	0,02415145	12425	NM_134196	V1re7	0	1	0	0	0	0	0	0	0	1	none
chr17:210488 chr17	-	21048867	21048867	0,14490867	21251	NM_134196	V1re7	1	1	0	0	0	0	1	0	0	0	LINE
chr17:210588 chr17	-	21058864	21058864	0,02415145	7563	NM_134193	V1re4	0	1	0	0	0	0	0	0	0	1	none
chr17:210820 chr17	-	21082053	21082053	0,02415145	30752	NM_134193	V1re4	0	1	1	0	0	0	0	0	0	0	none
chr17:210824 chr17	-	21082409	21082409	0,07245434	31108	NM_134193	V1re4	0	1	1	1	0	0	0	0	1	0	none
chr17:211333 chr17	-	21133358	21133358	0,02415145	1707	NM_134202	V1rf5	0	1	0	0	0	0	1	0	1	1	none
chr17:211399 chr17	-	21139951	21139951	0,07245434	8300	NM_134202	V1rf5	1	0	0	0	0	0	0	0	0	0	LINE
chr17:211401 chr17	-	21140158	21140158	0,21736301	8507	NM_134202	V1rf5	1	0	0	0	0	0	0	0	0	0	LINE
chr17:211419 chr17	-	21141927	21141927	0,04830289	10276	NM_134202	V1rf5	1	0	0	0	0	0	0	0	0	0	LINE
chr17:211693 chr17	+	21169303	21169303	0,14490867	196486	NM_134198	V1rf1	0	1	0	0	0	0	1	1	0	0	none
chr17:211693 chr17	+	21169375	21169375	0,02415145	196414	NM_134198	V1rf1	0	1	0	0	0	0	1	1	0	0	none
chr17:211798 chr17	+	21179897	21179897	0,04830289	185892	NM_134198	V1rf1	0	0	0	0	0	0	0	0	0	0	none
chr17:211829 chr17	+	21182989	21182989	0,02415145	182800	NM_134198	V1rf1	1	0	0	0	0	0	0	0	0	0	LINE
chr17:211959 chr17	+	21195928	21195928	0,14490867	169861	NM_134198	V1rf1	0	1	0	0	0	0	0	0	0	0	none
chr17:212089 chr17	+	21208976	21208976	0,07245434	156813	NM_134198	V1rf1	1	0	0	0	0	0	0	0	0	0	LINE
chr17:212105 chr17	+	21210593	21210593	0,02415145	155196	NM_134198	V1rf1	1	0	0	0	0	0	0	0	0	0	LINE
chr17:212158 chr17	+	21215803	21215803	0,09660578	149986	NM_134198	V1rf1	1	0	0	0	0	0	0	0	0	0	LINE
chr17:212282 chr17	+	21228206	21228206	0,14490867	137583	NM_134198	V1rf1	0	0	0	0	0	0	0	0	0	0	none
chr17:212447 chr17	+	21244731	21244731	0,09660578	121058	NM_134198	V1rf1	0	0	0	0	0	0	0	0	0	0	none

chr17:213232 chr17	+	21323201	21323201	0,02415145	42588	NM_134198	V1rf1	0	0	0	0	0	0	1	0	none
chr17:213560 chr17	+	21356069	21356069	0,02415145	9720	NM_134198	V1rf1	1	0	0	0	0	0	0	0	LTR
chr17:214002 chr17	+	21400242	21400253	0,21736301	23343	NM_134201	V1rf4	0	1	0	0	0	1	1	0	none
chr17:214113 chr17	+	21411363	21411363	0,02415145	12222	NM_134201	V1rf4	1	0	0	0	0	0	0	0	LINE
chr17:214178 chr17	+	21417867	21417867	0,02415145	5718	NM_134201	V1rf4	1	0	0	0	0	0	0	0	LINE
chr17:214368 chr17	+	21436847	21436864	0,14490867	14116	NM_134200	V1rf3	1	0	0	0	0	0	0	0	LINE
chr17:214427 chr17	+	21442709	21442709	0,04830289	8271	NM_134200	V1rf3	1	0	0	0	0	0	0	0	LINE
chr6:5687402 chr6	+	56874026	56874026	0,12075723	32480	NM_134176	V1rc21	0	1	1	1	0	0	1	0	none
chr6:5687426 chr6	+	56874267	56874267	0,09660578	32239	NM_134176	V1rc21	0	1	1	1	0	0	1	0	none
chr6:5687432 chr6	+	56874322	56874331	0,14490867	32175	NM_134176	V1rc21	0	1	1	1	0	0	1	0	none
chr6:5687463 chr6	+	56874630	56874630	0,07245434	31876	NM_134176	V1rc21	0	1	1	1	0	0	0	0	none
chr6:5687535 chr6	+	56875350	56875350	0,12075723	31156	NM_134176	V1rc21	1	1	0	1	0	0	0	0	LTR
chr6:5687564 chr6	+	56875640	56875640	0,02415145	30866	NM_134176	V1rc21	1	1	0	1	0	0	0	0	LTR
chr6:5687834 chr6	+	56878345	56878345	0,02415145	28161	NM_134176	V1rc21	1	0	0	0	0	0	0	0	LTR
chr6:5687940 chr6	+	56879409	56879412	0,36227168	27097	NM_134176	V1rc21	1	0	0	0	0	0	0	0	LINE
chr6:5689682 chr6	+	56896820	56896859	0,82114914	9686	NM_134176	V1rc21	0	0	0	0	0	1	1	1	none
chr6:5689688 chr6	+	56896882	56896882	0,09660578	9624	NM_134176	V1rc21	0	0	0	0	0	1	1	1	none
chr6:5689690 chr6	+	56896908	56896908	0,02415145	9598	NM_134176	V1rc21	0	0	0	0	0	1	1	1	none
chr6:5690757 chr6	+	56907571	56907571	0,02415145	27764	NM_134174	V1rc19	0	1	0	0	0	0	0	0	none
chr6:5691747 chr6	+	56917478	56917478	0,04830289	17857	NM_134174	V1rc19	1	0	0	0	0	0	0	0	LINE
chr6:5691787 chr6	+	56917873	56917873	0,14490867	17462	NM_134174	V1rc19	0	0	0	0	0	0	0	0	none
chr6:5693176 chr6	+	56931763	56931771	0,04830289	3572	NM_134174	V1rc19	1	0	0	0	0	0	0	0	LINE
chr6:5698199 chr6	+	56981994	56981994	0,02415145	3965	NM_134187	V1rc32	0	1	0	0	0	0	0	1	none
chr6:5698412 chr6	+	56984120	56984120	0,12075723	1839	NM_134187	V1rc32	0	1	0	0	0	1	0	1	none
chr6:5698496 chr6	+	56984969	56984969	0,02415145	990	NM_134187	V1rc32	1	1	0	0	0	0	1	1	LINE
chr6:5700596 chr6	+	57005961	57005963	0,09660578	14974	NM_134185	V1rc30	1	0	0	0	0	0	0	0	LINE
chr6:5700598 chr6	+	57005988	57005988	0,16906012	14947	NM_134185	V1rc30	1	0	0	0	0	0	0	0	LINE
chr6:5700725 chr6	+	57007251	57007251	0,02415145	13684	NM_134185	V1rc30	1	0	0	0	0	0	0	0	LINE
chr6:5703402 chr6	+	57034022	57034022	0,02415145	29396	NM_053231	V1rc1	1	0	0	0	0	0	0	0	LINE
chr6:5705923 chr6	+	57059231	57059231	0,14490867	4187	NM_053231	V1rc1	0	1	0	0	0	0	0	1	none
chr6:5707389 chr6	+	57073898	57073898	0,14490867	13448	NM_053233	V1rc3	1	0	0	0	0	0	0	0	LINE
chr6:5707396 chr6	+	57073965	57073965	0,07245434	13381	NM_053233	V1rc3	1	0	0	0	0	0	0	0	LINE
chr6:5708285 chr6	+	57082856	57082856	0,16906012	4490	NM_053233	V1rc3	1	1	0	0	0	0	0	1	LINE
chr6:5711931 chr6	+	57119317	57119317	0,12075723	40534	NM_053235	V1rc5	1	0	0	0	0	0	0	0	LINE
chr6:5712056 chr6	+	57120568	57120568	0,02415145	39283	NM_053235	V1rc5	1	0	0	0	0	0	0	0	LINE
chr6:5713593 chr6	+	57135930	57135930	0,02415145	23921	NM_053235	V1rc5	0	1	0	0	0	0	1	0	none
chr6:5714172 chr6	+	57141725	57141725	0,02415145	18126	NM_053235	V1rc5	1	0	0	0	0	0	0	0	LTR
chr6:5714426 chr6	+	57144265	57144277	0,04830289	15586	NM_053235	V1rc5	1	0	0	0	0	0	0	0	LINE
chr6:5714777 chr6	+	57147774	57147775	0,09056792	12076	NM_053235	V1rc5	0	0	0	0	0	0	0	0	none
chr6:5715627 chr6	+	57156273	57156273	0,07245434	3578	NM_053235	V1rc5	0	1	0	0	0	0	0	1	none
chr6:5715663 chr6	+	57156634	57156634	0,02415145	3217	NM_053235	V1rc5	0	1	0	0	0	0	0	1	none
chr6:5715917 chr6	+	57159176	57159176	0,02415145	675	NM_053235	V1rc5	0	0	0	0	0	1	1	1	none
chr6:5716357 chr6	+	57163575	57163575	0,07245434	19857	NM_053237	V1rc7	1	0	0	0	0	0	0	0	LINE
chr6:5716734 chr6	+	57167343	57167343	0,02415145	16089	NM_053237	V1rc7	1	0	0	0	0	0	0	0	LINE
chr6:5719607 chr6	+	57196074	57196074	0,02415145	12068	NM_053236	V1rc6	1	0	0	0	0	0	0	0	LINE
chr6:5720437 chr6	+	57204375	57204375	0,02415145	3767	NM_053236	V1rc6	0	1	0	0	0	0	1	1	none
chr6:5729901 chr6	-	57299015	57299015	0,04830289	25385	NM_134184	V1rc29	1	0	0	0	0	0	0	0	LINE
chr6:5731474 chr6	-	57314747	57314749	0,24151445	3376	NM_134171	V1rc16	1	0	0	0	0	0	0	0	LINE
chr6:5732821 chr6	-	57328219	57328219	0,04830289	16846	NM_134171	V1rc16	1	0	0	0	0	0	0	0	LTR
chr6:5733535 chr6	-	57335350	57335350	0,04830289	23977	NM_134171	V1rc16	1	0	0	0	0	0	0	0	LINE
chr6:5734295 chr6	+	57342958	57342958	0,02415145	11499	NM_134182	V1rc27	0	1	0	0	0	0	0	0	none
chr6:5734306 chr6	+	57343060	57343060	0,02415145	11397	NM_134182	V1rc27	0	1	0	0	0	0	0	0	none
chr6:5734416 chr6	-	57344163	57344163	0,04830289	3601	NM_134181	V1rc26	0	1	0	0	0	0	0	1	none
chr6:5734462 chr6	-	57344624	57344624	0,16906012	4062	NM_134181	V1rc26	0	1	0	0	0	1	0	1	none
chr6:5734937 chr6	-	57349378	57349378	0,07245434	8816	NM_134181	V1rc26	1	1	0	0	0	0	0	0	LTR
chr6:5735057 chr6	+	57350570	57350570	0,07245434	3887	NM_134182	V1rc27	0	1	0	0	0	0	0	1	none
chr6:5779465 chr6	-	57794651	57794655	0,12075723	203	NM_134183	V1rc28	0	1	0	0	0	1	1	1	none
chr6:5780771 chr6	-	57807716	57807716	0,04830289	13264	NM_134183	V1rc28	1	0	0	0	0	0	0	0	LINE
chr6:5780868 chr6	-	57808686	57808686	0,04830289	14234	NM_134183	V1rc28	1	0	0	0	0	0	0	0	Other
chr6:5781773 chr6	-	57817733	57817733	0,00966058	23281	NM_134183	V1rc28	1	0	0	0	0	0	0	0	LINE
chr6:5781779 chr6	-	57817793	57817793	0,04830289	23341	NM_134183	V1rc28	1	0	0	0	0	0	0	0	LINE
chr6:5782101 chr6	-	57821017	57821017	0,02415145	26565	NM_134183	V1rc28	1	1	0	0	0	0	0	0	LINE
chr6:5787911 chr6	-	57879113	57879113	0,09660578	2327	NM_134179	V1rc24	0	0	0	0	0	1	0	1	LINE
chr6:5788039 chr6	-	57880398	57880398	0,02415145	3612	NM_134179	V1rc24	1	1	0	0	0	1	0	1	SINE
chr6:5791106 chr6	-	57911063	57911063	0,02415145	4537	NM_134173	V1rc18	0	1	0	0	0	0	0	1	none
chr6:5791512 chr6	-	57915129	57915129	0,02415145	8603	NM_134173	V1rc18	1	0	0	0	0	0	0	0	LINE
chr6:5791883 chr6	-	57918836	57918836	0,02415145	12310	NM_134173	V1rc18	1	0	0	0	0	0	0	0	LTR
chr6:5793032 chr6	-	57930327	57930328	0,24151445	1031	NM_053238	V1rc8	1	1	0	0	0	1	0	1	LINE
chr6:5794068 chr6	-	57940685	57940685	0,02415145	11388	NM_053238	V1rc8	1	0	0	0	0	0	0	1	LTR
chr6:5794577 chr6	-	57945771	57945771	0,02415145	16474	NM_053238	V1rc8	1	0	0	0	0	0	0	0	LINE
chr6:5795397 chr6	-	57953972	57953972	0,02415145	24675	NM_053238	V1rc8	1	0	0	0	0	0	0	0	LINE
chr6:5797787 chr6	-	57977871	57977871	0,02415145	18674	NM_134172	V1rc17	1	0	0	0	0	0	0	0	SINE

chr6:8988694	chr6	-	89886942	89886942	0,02415145	2961	NM_011684	V1ra2	1	1	0	0	0	0	0	0	LTR
chr6:8988852	chr6	-	89888527	89888527	0,02415145	4546	NM_011684	V1ra2	0	1	0	0	0	0	0	0	none
chr6:8989208	chr6	-	89892088	89892088	0,02415145	8107	NM_011684	V1ra2	1	0	0	0	0	0	0	0	LINE
chr6:8990167	chr6	-	89901675	89901675	0,02415145	17694	NM_011684	V1ra2	1	0	0	0	0	0	0	0	LINE
chr6:8990265	chr6	-	89902653	89902653	0,02415145	18672	NM_011684	V1ra2	1	0	0	0	0	0	0	0	LINE
chr6:8992132	chr6	+	89921322	89921322	0,12075723	4842	NM_053229	V1rb8	0	1	0	0	0	0	0	1	none
chr6:8992142	chr6	+	89921424	89921424	0,02415145	4740	NM_053229	V1rb8	1	0	0	0	0	0	0	1	none
chr6:8992517	chr6	+	89925171	89925171	0,12075723	993	NM_053229	V1rb8	0	1	0	0	0	0	1	1	none
chr6:8992532	chr6	+	89925321	89925321	0,02415145	843	NM_053229	V1rb8	1	1	0	0	0	0	1	1	SINE
chr6:8994163	chr6	+	89941631	89941632	0,09660578	30250	NM_053219	V1ra4	1	0	0	0	0	0	0	0	LTR
chr6:9003717	chr6	+	90037171	90037171	0,02415145	20097	NM_053225	V1rb1	1	0	0	0	0	0	0	0	LTR
chr6:9004248	chr6	+	90042484	90042484	0,09660578	14784	NM_053225	V1rb1	1	0	0	0	0	0	0	0	LINE
chr6:9004707	chr6	+	90047079	90047079	0,02415145	10189	NM_053225	V1rb1	0	0	0	0	0	0	0	0	none
chr6:9005422	chr6	+	90054220	90054220	0,04830289	3048	NM_053225	V1rb1	0	1	0	0	0	0	0	1	none
chr6:9005880	chr6	+	90058809	90058809	0,02415145	20288	NM_011683	V1ra1	0	1	0	0	0	0	0	0	none
chr6:9007620	chr6	+	90076206	90076206	0,16906012	2891	NM_011683	V1ra1	1	1	0	0	0	0	0	0	LTR
chr6:9009243	chr6	+	90092431	90092431	0,02415145	36278	NM_053222	V1ra7	1	0	0	0	0	0	0	0	LINE
chr6:9009286	chr6	+	90092865	90092865	0,21736301	35844	NM_053222	V1ra7	1	0	0	0	0	0	0	0	LINE
chr6:9011104	chr6	+	90111045	90111045	0,04830289	17664	NM_053222	V1ra7	0	1	0	0	0	0	0	0	none
chr6:9012084	chr6	+	90120843	90120843	0,24151445	7866	NM_053222	V1ra7	1	0	0	0	0	0	0	1	LINE
chr6:9013135	chr6	+	90131359	90131359	0,02415145	21451	NM_053223	V1ra8	1	0	0	0	0	0	0	0	LTR
chr6:9013472	chr6	+	90134720	90134720	0,12075723	18090	NM_053223	V1ra8	1	0	0	0	0	0	0	0	LINE
chr6:9014922	chr6	+	90149228	90149228	0,41057457	3582	NM_053223	V1ra8	0	0	0	0	0	0	1	0	none
chr6:9018568	chr6	+	90185687	90185687	0,14490867	33412	NM_053224	V1ra9	0	0	0	0	0	0	1	0	none
chr6:9018779	chr6	+	90187799	90187799	0,07245434	31300	NM_053224	V1ra9	0	0	0	0	0	0	1	1	none
chr6:9019499	chr6	-	90194993	90194993	0,12075723	20658	NM_053226	V1rb3	0	0	0	0	0	0	0	0	none
chr6:9021781	chr6	+	90217810	90217813	0,38642312	1286	NM_053224	V1ra9	1	0	0	0	0	0	0	0	LTR
chr7:1086109	chr7	+	10861093	10861093	0,02415145	171066	NM_134229	V1re10	0	0	0	0	0	0	1	0	none
chr7:1086174	chr7	-	10861744	10861744	0,12075723	1290	NM_134230	V1re11	0	0	0	0	0	0	0	1	none
chr7:1086534	chr7	+	10865341	10865341	0,02415145	166818	NM_134229	V1re10	1	0	0	0	0	0	1	0	LINE
chr7:1086813	chr7	-	10868138	10868153	0,04830289	7684	NM_134230	V1re11	0	0	0	0	0	0	1	0	none
chr7:1086874	chr7	-	10868744	10868744	0,02415145	8290	NM_134230	V1re11	0	0	0	0	0	0	1	0	none
chr7:1088341	chr7	+	10883418	10883418	0,16906012	148741	NM_134229	V1re10	1	0	0	0	0	0	0	0	LTR
chr7:1088759	chr7	+	10887596	10887596	0,14490867	144563	NM_134229	V1re10	0	0	0	0	0	0	0	0	none
chr7:1091415	chr7	+	10914158	10914158	0,02415145	118001	NM_134229	V1re10	1	1	0	0	0	0	0	0	SINE
chr7:1091949	chr7	+	10919494	10919494	0,07245434	112665	NM_134229	V1re10	1	0	0	0	0	0	0	0	LINE
chr7:1092247	chr7	+	10922472	10922472	0,16906012	109687	NM_134229	V1re10	1	0	0	0	0	0	0	0	LINE
chr7:1092268	chr7	+	10922682	10922682	0,02415145	109477	NM_134229	V1re10	1	0	0	0	0	0	0	0	LINE
chr7:1092381	chr7	+	10923812	10923812	0,02415145	108347	NM_134229	V1re10	1	0	0	0	0	0	0	0	LINE
chr7:1092497	chr7	+	10924972	10924972	0,02415145	64518	NM_134230	V1re11	0	1	0	0	0	0	0	0	none
chr7:1093735	chr7	-	10937355	10937355	0,12075723	76901	NM_134230	V1re11	0	1	0	0	0	0	0	0	none
chr7:1095189	chr7	+	10951896	10951896	0,16906012	80263	NM_134229	V1re10	1	0	0	0	0	0	0	0	LINE
chr7:1096560	chr7	+	10965600	10965600	0,02415145	66559	NM_134229	V1re10	1	0	0	0	0	0	0	0	SINE
chr7:1096562	chr7	-	10965627	10965630	0,12075723	105176	NM_134230	V1re11	1	0	0	0	0	0	0	0	SINE
chr7:1096572	chr7	+	10965720	10965720	0,01610096	105266	NM_134230	V1re11	0	0	0	0	0	0	1	0	none
chr7:1097817	chr7	+	10978173	10978173	0,02415145	53986	NM_134229	V1re10	1	1	0	0	0	0	0	0	LINE
chr7:1098087	chr7	+	10980874	10980874	0,33812023	51285	NM_134229	V1re10	1	0	0	0	0	0	0	0	LINE
chr7:1101833	chr7	-	11018331	11018331	0,12075723	157877	NM_134230	V1re11	1	0	0	0	0	0	0	0	LINE
chr7:1102335	chr7	-	11023354	11023354	0,02415145	162900	NM_134230	V1re11	1	0	0	0	0	0	1	0	LINE
chr7:1102345	chr7	-	11023452	11023452	0,02415145	162998	NM_134230	V1re11	1	0	0	0	0	0	1	0	LINE
chr7:1102672	chr7	-	11026725	11026725	0,07245434	166271	NM_134230	V1re11	1	0	0	0	0	0	0	0	none
chr7:1102803	chr7	-	11028036	11028036	0,12075723	167582	NM_134230	V1re11	1	1	0	0	0	0	1	0	LINE
chr7:1102814	chr7	-	11028148	11028148	0,02415145	167694	NM_134230	V1re11	0	0	0	0	0	0	1	0	none
chr7:1103199	chr7	-	11031992	11031994	0,12075723	171540	NM_134230	V1re11	0	1	0	0	0	0	1	1	none
chr7:1118229	chr7	+	11182298	11182298	0,21736301	36637	NM_134228	V1rl1	0	0	0	0	0	0	0	0	none
chr7:1118795	chr7	+	11187950	11187950	0,02415145	30985	NM_134228	V1rl1	1	0	0	0	0	0	0	0	LINE
chr7:1119104	chr7	+	11191048	11191048	0,12075723	27887	NM_134228	V1rl1	1	0	0	0	0	0	0	0	LTR
chr7:1121350	chr7	+	11213506	11213506	0,07245434	5429	NM_134228	V1rl1	0	0	0	0	0	0	0	1	none
chr7:1121425	chr7	-	11214250	11214250	0,14490867	48098	NM_145842	V1re9	0	0	0	0	0	0	1	0	none
chr7:1121502	chr7	+	11215027	11215027	0,43472601	3908	NM_134228	V1rl1	0	0	0	0	0	0	0	1	none
chr7:1121857	chr7	+	11218577	11218577	0,21736301	360	NM_134228	V1rl1	0	1	0	0	0	0	1	1	none
chr7:1137816	chr7	-	11378165	11378165	0,02415145	44056	NM_145848	V1re13	1	0	0	0	0	0	0	0	Unknown
chr7:1137822	chr7	-	11378224	11378224	0,02415145	44115	NM_145848	V1re13	1	0	0	0	0	0	0	0	DNA
chr7:1142715	chr7	-	11427152	11427154	0,04830289	93043	NM_145848	V1re13	1	0	0	0	0	0	0	0	LTR
chr7:1142721	chr7	-	11427219	11427219	0,07245434	93110	NM_145848	V1re13	1	0	0	0	0	0	0	0	LTR
chr7:1142724	chr7	-	11427244	11427253	0,0058549	93135	NM_145848	V1re13	1	0	0	0	0	0	0	0	LTR
chr7:1148346	chr7	-	11483464	11483464	0,02415145	149355	NM_145848	V1re13	1	0	0	0	0	0	0	0	LINE
chr7:1225934	chr7	-	12259340	12259340	0,02415145	3471	NM_145843	V1rg1	1	1	0	0	0	0	1	0	SINE
chr7:1225946	chr7	-	12259461	12259461	0,09660578	3592	NM_145843	V1rg1	0	1	0	0	0	0	1	0	none
chr7:1225953	chr7	-	12259532	12259532	0,02415145	3663	NM_145843	V1rg1	1	1	0	0	0	0	1	0	none
chr7:1226119	chr7	+	12261191	12261191	0,19321156	80414	NM_134203	V1rg2	1	1	0	0	0	0	1	0	LINE
chr7:1226453	chr7	+	12264531	12264531	0,21736301	77074	NM_134203	V1rg2	0	1	0	0	0	0	0	0	LINE

chr7:1226748 chr7	-	12267483	12267483	0,02415145	11614	NM_145843	V1rg1	1	1	0	0	0	0	0	0	1	LINE
chr7:1226775 chr7	-	12267757	12267757	0,02415145	11888	NM_145843	V1rg1	1	0	0	0	0	0	0	0	1	LINE
chr7:1226901 chr7	-	12269013	12269023	0,16906012	13154	NM_145843	V1rg1	1	0	0	0	0	0	0	0	1	LINE
chr7:1228900 chr7	-	12289008	12289008	0,02415145	33139	NM_145843	V1rg1	1	0	0	0	0	0	0	0	0	ow_complexity
chr7:1229701 chr7	+	12297017	12297017	0,02415145	44588	NM_134203	V1rg2	1	0	0	0	0	0	0	0	0	LINE
chr7:1229962 chr7	-	12299624	0,02415145	43755	NM_145843	V1rg1	0	1	0	0	0	0	0	0	0	0	none
chr7:1230737 chr7	-	12307379	12307379	0,02415145	51510	NM_145843	V1rg1	1	0	0	0	0	0	0	0	0	LTR
chr7:1230965 chr7	-	12309655	0,02415145	53786	NM_145843	V1rg1	1	1	0	0	0	0	0	0	0	0	LINE
chr7:1231116 chr7	+	12311169	12311169	0,04830289	30436	NM_134203	V1rg2	1	1	0	0	0	0	0	0	0	LINE
chr7:1231524 chr7	-	12315245	0,16906012	59376	NM_145843	V1rg1	1	1	0	0	0	0	0	0	0	0	LINE
chr7:1231807 chr7	-	12318070	0,04830289	62201	NM_145843	V1rg1	1	1	0	0	0	0	0	0	0	0	none
chr7:1232912 chr7	-	12329121	0,09660578	73252	NM_145843	V1rg1	1	0	0	0	0	0	0	0	0	0	LINE
chr7:1233423 chr7	-	12334235	0,14490867	78366	NM_145843	V1rg1	1	0	0	0	0	0	0	0	0	0	LINE
chr7:1233558 chr7	+	12335582	0,16906012	6023	NM_134203	V1rg2	0	1	0	0	0	0	0	0	0	1	none
chr7:1233962 chr7	+	12339627	12339627	0,04830289	1978	NM_134203	V1rg2	1	0	0	0	0	1	1	1	1	LINE
chr7:1234900 chr7	+	12349008	0,02415145	83115	NM_134206	V1rg5	1	0	0	0	0	0	0	0	0	0	LINE
chr7:1234954 chr7	+	12349543	12349543	0,21736301	82580	NM_134206	V1rg5	1	0	0	0	0	0	0	0	0	LINE
chr7:1234991 chr7	+	12349918	0,07245434	82205	NM_134206	V1rg5	1	0	0	0	0	0	0	0	0	0	LINE
chr7:1235075 chr7	+	12350751	12350751	0,09660578	81372	NM_134206	V1rg5	1	0	0	0	0	0	0	0	0	LINE
chr7:1235402 chr7	+	12354022	0,02415145	78101	NM_134206	V1rg5	1	0	0	0	0	0	0	0	0	0	LINE
chr7:1235731 chr7	+	12357315	0,02415145	74808	NM_134206	V1rg5	1	0	0	0	0	0	0	0	0	0	LINE
chr7:1236375 chr7	+	12363755	12363762	0,03622717	68361	NM_134206	V1rg5	1	0	0	0	0	0	0	0	0	LINE
chr7:1236715 chr7	+	12367157	0,04830289	64966	NM_134206	V1rg5	0	1	0	0	0	0	0	0	0	0	none
chr7:1236721 chr7	+	12367213	0,21736301	64910	NM_134206	V1rg5	0	1	0	0	0	0	0	0	0	0	none
chr7:1240063 chr7	+	12400635	0,02415145	31488	NM_134206	V1rg5	1	0	0	0	0	0	0	0	0	0	LTR
chr7:1240763 chr7	+	12407639	0,43472601	24484	NM_134206	V1rg5	0	0	0	0	0	0	0	0	0	0	none
chr7:1240891 chr7	+	12408918	0,02415145	23205	NM_134206	V1rg5	1	0	0	0	0	0	0	0	0	0	LTR
chr7:1241463 chr7	+	12414637	0,16906012	17486	NM_134206	V1rg5	1	0	0	0	0	0	0	0	0	0	LTR
chr7:1241770 chr7	+	12417709	0,02415145	14414	NM_134206	V1rg5	0	0	0	0	0	0	0	0	0	0	none
chr7:1241942 chr7	+	12419432	0,21736301	12695	NM_134206	V1rg5	0	0	0	0	0	0	0	0	0	0	none
chr7:1242046 chr7	+	12420466	0,09660578	11657	NM_134206	V1rg5	0	0	0	0	0	0	0	0	0	0	none
chr7:1242442 chr7	+	12424426	0,16906012	7697	NM_134206	V1rg5	1	0	0	0	0	0	0	1	1	1	LINE
chr7:1245241 chr7	+	12452412	0,07698273	13279	NM_134207	V1rg6	0	0	0	0	0	0	1	1	1	0	none
chr7:1245246 chr7	+	12452466	0,04321702	13203	NM_134207	V1rg6	0	0	0	0	0	0	1	1	1	0	none
chr7:1246161 chr7	+	12461611	0,02415145	4080	NM_134207	V1rg6	0	1	0	0	0	0	0	0	0	1	none
chr7:1246450 chr7	+	12464509	0,02415145	1182	NM_134207	V1rg6	0	1	0	0	0	0	1	1	1	1	none
chr7:1252199 chr7	-	12521998	0,02415145	5363	NM_134205	V1rg4	0	1	0	0	0	0	0	0	0	1	none
chr7:1252204 chr7	-	12522046	0,02415145	5411	NM_134205	V1rg4	0	1	0	0	0	0	0	0	0	1	none
chr7:1253380 chr7	-	12533801	0,14490867	17166	NM_134205	V1rg4	0	0	0	0	0	0	0	0	0	0	none
chr7:1253431 chr7	+	12534315	0,02415145	203497	NM_134208	V1rg7	0	1	0	0	0	0	0	0	0	0	none
chr7:1254417 chr7	+	12544179	0,02415145	193633	NM_134208	V1rg7	0	0	0	0	0	0	0	1	1	0	none
chr7:1254719 chr7	-	12547197	0,04830289	30562	NM_134205	V1rg4	1	0	0	0	0	0	0	0	0	0	LTR
chr7:1254725 chr7	-	12547253	0,02415145	30618	NM_134205	V1rg4	1	0	0	0	0	0	0	0	0	0	LTR
chr7:1255064 chr7	+	12550645	0,02415145	187167	NM_134208	V1rg7	0	0	0	0	0	0	0	0	0	0	LINE
chr7:1255260 chr7	-	12552605	0,09660578	35974	NM_134205	V1rg4	1	0	0	0	0	0	0	0	0	0	snRNA,LINE
chr7:1255339 chr7	+	12553390	0,02415145	184422	NM_134208	V1rg7	0	0	0	0	0	0	0	0	0	0	none
chr7:1255690 chr7	+	12556903	0,0049902	180912	NM_134208	V1rg7	0	1	0	0	0	0	1	1	1	0	none
chr7:1255693 chr7	+	12556930	0,24151445	180882	NM_134208	V1rg7	0	1	0	0	0	0	1	1	1	0	none
chr7:1255783 chr7	+	12557837	0,38642312	179975	NM_134208	V1rg7	0	1	0	0	0	0	1	1	1	0	none
chr7:1257518 chr7	+	12575188	0,04830289	162624	NM_134208	V1rg7	1	1	0	0	0	0	0	0	0	0	SINE
chr7:1257819 chr7	+	12578192	0,02415145	159620	NM_134208	V1rg7	0	1	0	0	0	0	0	0	0	0	LINE
chr7:1257849 chr7	-	12578492	0,12075723	61857	NM_134205	V1rg4	1	1	0	0	0	0	0	0	0	0	Simple_repeat
chr7:1257874 chr7	-	12578746	0,16906012	62111	NM_134205	V1rg4	0	1	0	0	0	0	0	0	0	0	LTR
chr7:1258403 chr7	-	12584039	1,40078382	67411	NM_134205	V1rg4	0	0	0	0	0	0	1	1	1	0	none
chr7:1258409 chr7	-	12584099	0,16906012	67466	NM_134205	V1rg4	0	0	0	0	0	0	1	1	1	0	none
chr7:1258484 chr7	-	12584849	0,02415145	68214	NM_134205	V1rg4	0	0	0	0	0	0	1	1	1	0	none
chr7:1258595 chr7	-	12585953	0,02415145	69318	NM_134205	V1rg4	0	1	0	0	0	0	0	0	0	0	none
chr7:1258805 chr7	-	12588053	0,02415145	71418	NM_134205	V1rg4	1	1	0	0	0	0	0	0	0	0	LINE
chr7:1259589 chr7	+	12595893	0,07245434	141919	NM_134208	V1rg7	0	0	0	0	0	0	1	1	1	0	none
chr7:1260337 chr7	+	12603379	0,02415145	86744	NM_134205	V1rg4	1	0	0	0	0	0	0	0	0	0	Simple_repeat
chr7:1262051 chr7	+	12620512	0,02415145	117300	NM_134208	V1rg7	0	0	0	0	0	0	0	0	0	0	none
chr7:1263170 chr7	+	12631706	0,12075723	106106	NM_134208	V1rg7	0	1	0	0	0	0	0	0	0	0	none
chr7:1263196 chr7	-	12631960	0,04830289	115325	NM_134205	V1rg4	1	1	0	0	0	0	0	0	0	0	LINE
chr7:1267193 chr7	-	12671932	0,04985518	155300	NM_134205	V1rg4	1	0	0	0	0	0	1	1	1	0	LTR
chr7:1267200 chr7	-	12672002	0,07438645	155369	NM_134205	V1rg4	1	0	0	0	0	0	1	1	1	0	LTR
chr7:1267203 chr7	-	12672031	0,12075723	155396	NM_134205	V1rg4	0	0	0	0	0	0	1	1	1	0	none
chr7:1267206 chr7	-	12672066	0,07245434	155431	NM_134205	V1rg4	0	0	0	0	0	0	1	1	1	0	none
chr7:1267608 chr7	+	12676089	0,04830289	61723	NM_134208	V1rg7	0	1	0	0	0	0	1	1	1	0	none
chr7:1268506 chr7	+	12685064	0,02415145	168429	NM_134205	V1rg4	0	0	0	0	0	0	0	0	0	0	none
chr7:1269033 chr7	+	12690336	0,02415145	47476	NM_134208	V1rg7	0	1	0	0	0	0	0	0	0	0	none
chr7:1269764 chr7	+	12697642	0,07245434	40170	NM_134208	V1rg7	1	0	0	0	0	0	0	0	0	0	LTR
chr7:1270046 chr7	-	12700462	0,02415145	183827	NM_134205	V1rg4	0	0	0	0	0	0	0	0	0	0	none

[illegible]

chr7:2469452 chr7	+	24694523	24694523	0,02415145	18881	NM_207545	V1rd17	1	0	0	0	0	0	0	0	0	LTR
chr7:2469757 chr7	+	24697578	24697578	0,09660578	15826	NM_207545	V1rd17	1	1	0	0	0	0	0	0	0	SINE
chr7:2470392 chr7	+	24703925	24703925	0,04830289	9479	NM_207545	V1rd17	0	0	0	0	0	0	0	0	0	none
chr7:2473698 chr7	+	24736989	24736989	0,14490867	443	NM_206869	V1rd16	0	0	0	0	0	0	1	0	1	none
chr7:2473703 chr7	+	24737039	24737039	0,02415145	393	NM_206869	V1rd16	0	0	0	0	0	0	1	0	1	none
chr7:2473889 chr7	+	24738890	24738890	0,02415145	30240	NM_207546	V1rd20	0	0	0	0	0	0	1	0	0	none
chr7:2474028 chr7	+	24740281	24740292	0,21736301	28849	NM_207546	V1rd20	1	0	0	0	0	0	1	0	0	LINE
chr7:2474055 chr7	+	24740558	24740558	0,41057457	28572	NM_207546	V1rd20	0	0	0	0	0	0	1	0	0	none
chr7:2474058 chr7	+	24740583	24740583	0,33812023	28547	NM_207546	V1rd20	0	0	0	0	0	0	1	0	0	none
chr7:2474234 chr7	+	24742349	24742349	0,02415145	26781	NM_207546	V1rd20	1	0	0	0	0	0	0	0	0	LINE
chr7:2475909 chr7	+	24759095	24759095	0,12075723	10035	NM_207546	V1rd20	1	0	0	0	0	0	0	0	0	Other
chr7:2476493 chr7	+	24764938	24764938	0,02415145	4192	NM_207546	V1rd20	1	1	0	0	0	0	0	0	1	LINE
chr7:2477216 chr7	+	24772164	24772164	0,02415145	15965	NM_207619	V1rd19	1	0	0	0	0	0	0	0	0	LINE
chr7:2479305 chr7	+	24793053	24793054	0,21736301	46739	NM_203489	V1rd15	1	0	0	0	0	0	0	0	0	LINE
chr7:2479561 chr7	+	24795616	24795616	0,02415145	44176	NM_203489	V1rd15	1	0	0	0	0	0	0	0	0	LINE
chr7:2482226 chr7	+	24822264	24822264	0,02415145	17528	NM_203489	V1rd15	1	0	0	0	0	0	0	0	0	LTR
chr7:2482273 chr7	+	24822737	24822737	0,33812023	17055	NM_203489	V1rd15	1	0	0	0	0	0	0	0	0	LTR
chr7:2742613 chr7	-	27426138	27426138	0,02415145	29040	NM_134231	V1re12	1	1	0	0	0	0	0	0	0	LINE
chr7:2742744 chr7	-	27427446	27427446	0,14490867	30348	NM_134231	V1re12	0	1	0	0	0	0	0	0	0	none
chr7:2743211 chr7	-	27432112	27432112	0,21736301	35014	NM_134231	V1re12	1	0	0	0	0	0	0	0	0	LINE
chr7:2743702 chr7	-	27437028	27437028	0,02415145	39930	NM_134231	V1re12	1	0	0	0	0	0	0	0	0	LTR
chr7:5213757 chr7	-	5213757	5213757	0,14490867	65538	NM_030740	V1rd3	0	0	0	0	0	0	0	0	0	none
chr7:5230369 chr7	-	5230369	5230369	0,12075723	82150	NM_030740	V1rd3	0	0	0	0	0	0	0	0	0	none
chr7:5236800 chr7	-	5236800	5236800	0,04830289	88581	NM_030740	V1rd3	1	0	0	0	0	0	0	0	0	LINE
chr7:5238394 chr7	-	5238394	5238395	0,07245434	90176	NM_030740	V1rd3	1	0	0	0	0	0	0	0	0	DNA
chr7:5248847 chr7	-	5248847	5248847	0,02415145	100628	NM_030740	V1rd3	0	0	0	0	0	0	1	0	0	none
chr7:5374748 chr7	-	5374748	5374748	0,09660578	11916	NM_030739	V1rd4	1	0	0	0	0	0	0	0	0	LINE
chr7:5395109 chr7	-	5395109	5395109	0,02415145	32277	NM_030739	V1rd4	1	0	0	0	0	0	0	0	0	LINE
chr7:5449768 chr7	+	5449768	5449768	0,02415145	177155	NM_207542	V1rd8	0	0	0	0	0	0	0	0	0	none
chr7:5496834 chr7	+	5496834	5496834	0,02415145	130089	NM_207542	V1rd8	0	1	0	1	0	0	1	1	0	none
chr7:5522072 chr7	+	5522072	5522072	0,07245434	104851	NM_207542	V1rd8	1	0	0	0	0	0	0	0	0	LINE
chr7:5523977 chr7	+	5523977	5523977	0,02415145	102946	NM_207542	V1rd8	1	0	0	0	0	0	0	0	0	LINE
chr7:5525469 chr7	+	5525469	5525469	0,02415145	101454	NM_207542	V1rd8	0	1	0	0	0	0	0	0	0	none
chr7:5531778 chr7	+	5531778	5531778	0,02415145	95145	NM_207542	V1rd8	1	0	0	0	0	0	0	0	0	LINE
chr7:5547413 chr7	+	5547413	5547413	0,02415145	79510	NM_207542	V1rd8	1	0	0	0	0	0	0	0	0	LINE
chr7:5560847 chr7	+	5560847	5560847	0,07245434	66076	NM_207542	V1rd8	1	0	0	0	0	0	0	0	0	LINE
chr7:5582111 chr7	+	5582111	5582111	0,02415145	44812	NM_207542	V1rd8	1	0	0	0	0	0	0	0	0	LINE
chr7:5585958 chr7	+	5585958	5585958	0,02415145	40965	NM_207542	V1rd8	1	0	0	0	0	0	0	0	0	LINE
chr7:5798359 chr7	-	5798359	5798359	0,04830289	43125	NM_030742	V1rd1	1	0	0	0	0	0	0	0	0	LINE
chr7:5809863 chr7	-	5809863	5809863	0,02415145	54629	NM_030742	V1rd1	1	0	0	0	0	0	0	0	0	LTR
chr7:5859674 chr7	-	5859674	5859674	0,02415145	23529	NM_207544	V1rd11	1	0	0	0	0	0	0	0	0	LINE
chr7:5873766 chr7	-	5873766	5873766	0,02415145	37621	NM_207544	V1rd11	1	0	0	0	0	0	0	0	0	LTR
chr7:5878980 chr7	-	5878980	5878980	0,07245434	42835	NM_207544	V1rd11	1	0	0	0	0	0	0	0	0	LINE
chr7:5879005 chr7	-	5879005	5879005	0,19321156	42860	NM_207544	V1rd11	0	1	0	0	0	0	0	0	0	LINE
chr7:5890038 chr7	-	5890038	5890038	0,12075723	53893	NM_207544	V1rd11	0	1	0	0	0	0	0	0	0	none
chr7:5898263 chr7	-	5898263	5898265	0,2656659	62120	NM_207544	V1rd11	1	0	0	0	0	0	0	0	0	LINE
chr7:5913297 chr7	-	5913297	5913297	0,01207572	77152	NM_207544	V1rd11	1	0	0	0	0	0	0	0	0	Simple_repeat
chr7:5913356 chr7	-	5913356	5913356	0,02415145	77211	NM_207544	V1rd11	1	0	0	0	0	0	0	0	0	LINE
chr7:5918756 chr7	-	5918756	5918756	0,02415145	82611	NM_207544	V1rd11	0	1	0	1	0	0	1	1	0	none
chr7:5962144 chr7	-	5962144	5962144	0,02415145	1308	NM_030738	V1rd6	0	0	1	0	1	0	1	1	0	LTR
chr7:5964019 chr7	-	5964019	5964019	0,09660578	3183	NM_030738	V1rd6	0	1	0	0	0	0	0	0	1	none
chr7:5972656 chr7	-	5972656	5972656	0,02415145	11820	NM_030738	V1rd6	0	1	0	0	0	0	0	0	0	LINE
chr7:5984391 chr7	-	5984391	5984391	0,09660578	23555	NM_030738	V1rd6	1	0	0	0	0	0	0	0	0	LTR
chrX:1438939 chrX	-	143893926	143893944	0,16906012	5672	NM_053240	V1rb10	1	0	0	0	0	0	0	0	0	LINE
chrX:1438952 chrX	-	143895250	143895264	0,04830289	6996	NM_053240	V1rb10	0	1	0	0	0	0	1	1	0	none
chrX:1438953 chrX	-	143895322	143895343	0,00966058	7068	NM_053240	V1rb10	0	1	0	0	0	0	1	1	0	none
chrX:1438954 chrX	-	143895456	143895456	0,00241514	7202	NM_053240	V1rb10	0	1	0	0	0	0	1	1	0	none
chrX:1438955 chrX	-	143895510	143895510	0,02415145	7256	NM_053240	V1rb10	0	1	0	0	0	0	1	1	0	none
chrX:1438957 chrX	-	143895712	143895712	0,04830289	7458	NM_053240	V1rb10	0	1	0	0	0	0	1	1	0	none
chrX:1439005 chrX	-	143900521	143900521	0,04830289	12267	NM_053240	V1rb10	1	0	0	0	0	0	0	0	0	LINE
chrX:1439049 chrX	-	143904944	143904958	0,04830289	16690	NM_053240	V1rb10	0	1	1	1	1	1	1	0	0	none
chrX:1439049 chrX	-	143904979	143904979	0,14490867	16725	NM_053240	V1rb10	0	1	1	1	1	1	1	0	0	none
chrX:1439050 chrX	-	143905020	143905020	0,02415145	16766	NM_053240	V1rb10	0	1	1	1	1	1	1	0	0	none
chrX:1439050 chrX	-	143905090	143905090	0,21736301	16836	NM_053240	V1rb10	0	1	1	1	1	1	1	0	0	none
chrX:1439051 chrX	-	143905149	143905149	0,24151445	16895	NM_053240	V1rb10	0	1	1	1	1	1	1	0	0	none
chrX:1439051 chrX	-	143905171	143905174	0,33812023	16917	NM_053240	V1rb10	0	1	1	1	1	1	1	0	0	none
chrX:1439052 chrX	-	143905222	143905222	0,02415145	16968	NM_053240	V1rb10	0	1	1	1	1	1	1	0	0	none
chrX:1439052 chrX	-	143905245	143905288	3,01893065	16993	NM_053240	V1rb10	0	1	1	1	1	1	1	0	0	none
chrX:1439053 chrX	-	143905321	143905321	0,02415145	17067	NM_053240	V1rb10	0	1	1	1	1	1	1	0	0	none
chrX:1439054 chrX	-	143905441	143905441	0,12075723	17187	NM_053240	V1rb10	0	1	1	1	1	1	1	0	0	none
chrX:1439054 chrX	-	143905488	143905488	0,02415145	17234	NM_053240	V1rb10	0	1	1	1	1	1	1	0	0	none
chrX:1439055 chrX	-	143905520	143905520	0,02415145	17266	NM_053240	V1rb10	0	1	1	1	1	1	1	0	0	none

chrX:1439056 chrX	-	143905676	143905676	0,02415145	17422	NM_053240	V1rb10	0	1	1	1	1	1	0	0	none
chrX:1439061 chrX	-	143906107	143906107	0,12075723	17853	NM_053240	V1rb10	0	1	1	1	1	1	0	0	none

name	chrom	strand	start.lbase	end	exp.tpm.OE_SiM_MuM_04Nov09	distance to annotated TSS(bp)	refseqID	gene_symbol	repeat overlap	rat	human	horse	dog	opposm	platps	cons.clu.new	repeat Class
chr10:129867760..129867760	chr10	-	129867760	129867760		591	NM_001102602	Vmn2r85	0	1	0	0	0	0	0	1	none
chr10:129876300..129876300	chr10	-	129876300	129876300		9131	NM_001102602	Vmn2r85	0	1	0	0	0	0	0	0	none
chr10:129898507..129898507	chr10	-	129898507	129898507		5056	NM_001103365	Vmn2r86	1	0	0	0	0	0	0	0	LINE
chr10:129899806..129899806	chr10	-	129899806	129899806		6355	NM_001103365	Vmn2r86	0	1	0	0	0	0	0	0	none
chr10:129908127..129908127	chr10	-	129908127	129908127		14676	NM_001103365	Vmn2r86	0	1	0	0	0	1	0	0	none
chr10:129935116..129935116	chr10	-	129935116	129935116		180	NM_001103366	Vmn2r87	0	1	0	0	0	0	0	1	none
chr10:129948630..129948630	chr10	-	129948630	129948630		13694	NM_001103366	Vmn2r87	0	0	0	0	0	0	0	0	none
chr10:129956007..129956007	chr10	-	129956007	129956007		21071	NM_001103366	Vmn2r87	1	0	0	0	0	0	0	0	LINE
chr10:129960989..129961009	chr10	-	129960989	129961009		26053	NM_001103366	Vmn2r87	1	0	0	0	0	0	0	0	Simple_repeat
chr10:129963474..129963474	chr10	-	129963474	129963474		28538	NM_001103366	Vmn2r87	0	1	0	0	0	0	0	0	none
chr10:129972463..129972477	chr10	-	129972463	129972477		37541	NM_001103366	Vmn2r87	0	1	0	0	0	1	0	0	none
chr10:129972666..129972666	chr10	-	129972666	129972666		37730	NM_001103366	Vmn2r87	0	1	0	0	0	1	1	0	none
chr10:129972768..129972815	chr10	-	129972768	129972815		37832	NM_001103366	Vmn2r87	0	1	0	0	0	1	1	0	none
chr10:129972867..129972882	chr10	-	129972867	129972882		37931	NM_001103366	Vmn2r87	0	1	0	0	0	1	1	0	none
chr10:129973887..129973887	chr10	-	129973887	129973887		38951	NM_001103366	Vmn2r87	0	1	0	0	0	0	0	0	none
chr10:129978035..129978035	chr10	-	129978035	129978035		43099	NM_001103366	Vmn2r87	0	0	0	0	0	1	0	0	none
chr10:129978984..129978984	chr10	-	129978984	129978984		44048	NM_001103366	Vmn2r87	0	1	0	0	0	0	0	0	none
chr10:129981913..129981913	chr10	-	129981913	129981913		46977	NM_001103366	Vmn2r87	0	1	0	0	0	0	0	0	none
chr10:129987006..129987006	chr10	-	129987006	129987006		52070	NM_001103366	Vmn2r87	1	0	0	0	0	0	0	0	Simple_repeat
chr10:129990894..129990894	chr10	-	129990894	129990894		55958	NM_001103366	Vmn2r87	1	0	0	0	0	0	0	0	LTR
chr10:78565374..78565374+	chr10	+	78565374	78565374		46186	NM_001103368	Vmn2r80	0	1	0	0	0	0	0	0	none
chr10:78565416..78565416+	chr10	+	78565416	78565416		46144	NM_001103368	Vmn2r80	0	1	0	0	0	0	0	0	none
chr10:78596724..78596724+	chr10	+	78596724	78596724		14836	NM_001103368	Vmn2r80	1	0	0	0	0	0	0	0	LINE
chr10:78603490..78603490+	chr10	+	78603490	78603490		8070	NM_001103368	Vmn2r80	0	0	0	0	0	0	0	0	LTR
chr10:78604776..78604776+	chr10	+	78604776	78604776		6784	NM_001103368	Vmn2r80	1	0	0	0	0	0	0	0	LINE
chr10:78609029..78609029+	chr10	+	78609029	78609029		2531	NM_001103368	Vmn2r80	1	0	0	0	0	0	0	0	LINE
chr10:78678378..78678378+	chr10	+	78678378	78678378		32160	NM_175936	Vmn2r81	1	0	0	0	0	0	0	0	LINE
chr10:78680402..78680402+	chr10	+	78680402	78680402		30136	NM_175936	Vmn2r81	1	0	0	0	0	0	0	0	LTR
chr10:78685193..78685193+	chr10	+	78685193	78685193		25345	NM_175936	Vmn2r81	1	0	0	0	0	0	0	0	LTR
chr10:78701929..78701929+	chr10	+	78701929	78701929		8609	NM_175936	Vmn2r81	1	0	0	0	0	0	0	0	LINE
chr10:78703612..78703612+	chr10	+	78703612	78703612		6926	NM_175936	Vmn2r81	1	0	0	0	0	0	0	0	LINE
chr10:78784937..78784937+	chr10	+	78784937	78784937		34398	NM_001101572	Vmn2r82	0	0	0	0	0	0	0	0	none
chr10:78786105..78786106+	chr10	+	78786105	78786106		33230	NM_001101572	Vmn2r82	1	0	0	0	0	0	0	0	LINE
chr10:78817371..78817371+	chr10	+	78817371	78817371		1964	NM_001101572	Vmn2r82	1	0	0	0	0	0	0	0	LTR
chr10:78883564..78883564+	chr10	+	78883564	78883564		48138	NM_001104537	Vmn2r83	1	0	0	0	0	0	0	0	LTR
chr10:78885795..78885795+	chr10	+	78885795	78885795		45907	NM_001104537	Vmn2r83	1	0	0	0	0	0	0	0	LTR
chr10:78904191..78904191+	chr10	+	78904191	78904191		27511	NM_001104537	Vmn2r83	1	0	0	0	0	0	0	0	LINE
chr10:78921539..78921539+	chr10	+	78921539	78921539		10163	NM_001104537	Vmn2r83	0	0	0	0	0	0	0	0	LINE
chr17:17761641..17761641+	chr17	+	17761641	17761641		79263	NM_001104539	Vmn2r90	0	1	1	1	1	0	1	0	none
chr17:17761671..17761671+	chr17	+	17761671	17761671		79233	NM_001104539	Vmn2r90	0	1	1	1	1	0	1	0	none
chr17:17762106..17762106+	chr17	+	17762106	17762106		78798	NM_001104539	Vmn2r90	0	1	1	1	1	0	1	0	none
chr17:17762344..17762344+	chr17	+	17762344	17762344		78560	NM_001104539	Vmn2r90	0	1	1	1	1	0	1	0	none
chr17:17762445..17762445+	chr17	+	17762445	17762445		78459	NM_001104539	Vmn2r90	0	1	1	1	1	0	0	0	none
chr17:17762841..17762841+	chr17	+	17762841	17762841		78063	NM_001104539	Vmn2r90	0	1	1	1	1	0	0	0	none
chr17:17763265..17763265+	chr17	+	17763265	17763265		77639	NM_001104539	Vmn2r90	0	1	1	1	1	0	0	0	none
chr17:17774690..17774690+	chr17	+	17774690	17774690		66214	NM_001104539	Vmn2r90	1	1	1	0	0	0	0	0	LTR
chr17:17783048..17783048+	chr17	+	17783048	17783048		57856	NM_001104539	Vmn2r90	0	0	1	0	0	0	0	0	none
chr17:17785599..17785511+	chr17	+	17785599	17785511		55299	NM_001104539	Vmn2r90	0	0	0	0	0	0	0	0	none
chr17:17788619..17788619+	chr17	+	17788619	17788619		52285	NM_001104539	Vmn2r90	1	0	1	0	0	0	0	0	LINE
chr17:17788886..17788886+	chr17	+	17788886	17788886		52018	NM_001104539	Vmn2r90	1	0	1	0	0	0	0	0	Simple_repeat
chr17:17792341..17792348+	chr17	+	17792341	17792348		48563	NM_001104539	Vmn2r90	0	0	0	0	0	1	1	0	none
chr17:17805012..17805012+	chr17	+	17805012	17805012		35892	NM_001104539	Vmn2r90	0	1	0	0	0	0	0	0	none
chr17:17805496..17805496+	chr17	+	17805496	17805496		35408	NM_001104539	Vmn2r90	0	1	0	0	0	0	0	0	none
chr17:17825936..17825936+	chr17	+	17825936	17825936		14968	NM_001104539	Vmn2r90	1	0	0	0	0	0	0	0	LINE
chr17:17828287..17828287+	chr17	+	17828287	17828287		12617	NM_001104539	Vmn2r90	1	0	0	0	0	0	0	0	LINE
chr17:17840579..17840579+	chr17	+	17840579	17840579		325	NM_001104539	Vmn2r90	0	0	0	0	0	1	0	0	none
chr17:18113244..18113244+	chr17	+	18113244	18113244		108776	NM_001104540	Vmn2r91	1	0	0	0	0	0	0	0	LTR
chr17:18119744..18119744+	chr17	+	18119744	18119744		102276	NM_001104540	Vmn2r91	1	0	0	0	0	0	0	0	LTR
chr17:18122274..18122274+	chr17	+	18122274	18122274		99746	NM_001104540	Vmn2r91	1	0	0	0	0	0	0	0	LINE
chr17:18138531..18138531+	chr17	+	18138531	18138531		83489	NM_001104540	Vmn2r91	0	1	0	0	0	0	0	0	none
chr17:18149038..18149041+	chr17	+	18149038	18149041		72982	NM_001104540	Vmn2r91	1	0	0	0	0	0	0	0	LINE
chr17:18151878..18151878+	chr17	+	18151878	18151878		70142	NM_001104540	Vmn2r91	1	0	0	0	0	0	0	0	LINE
chr17:18173426..18173426+	chr17	+	18173426	18173426		48594	NM_001104540	Vmn2r91	1	0	0	0	0	0	0	0	LINE
chr17:18174371..18174371+	chr17	+	18174371	18174371		47649	NM_001104540	Vmn2r91	1	0	0	0	0	0	0	0	LINE
chr17:18174450..18174450+	chr17	+	18174450	18174450		47570	NM_001104540	Vmn2r91	1	0	0	0	0	0	0	0	LINE
chr17:18202367..18202367+	chr17	+	18202367	18202367		19653	NM_001104540	Vmn2r91	0	1	0	0	0	1	0	0	none
chr17:18206855..18206855+	chr17	+	18206855	18206855		15165	NM_001104540	Vmn2r91	1	1	0	0	0	0	0	0	LINE
chr17:18282905..18282905+	chr17	+	18282905	18282905		5988	NM_001104541	Vmn2r92	1	0	0	0	0	0	0	0	LINE
chr17:18324991..18324991+	chr17	+	18324991	18324991		1190601	NM_001104551	Vmn2r99	1	0	0	0	0	0	0	0	LTR
chr17:18342601..18342601+	chr17	+	18342601	18342601		1172991	NM_001104551	Vmn2r99	1	0	0	0	0	0	0	0	LTR
chr17:18372598..18372598+	chr17	+	18372598	18372598		1142994	NM_001104551	Vmn2r99	1	0	0	0	0	0	0	0	LTR
chr17:18376564..18376564+	chr17	+	18376564	18376564		1139028	NM_001104551	Vmn2r99	0	1	0	0	0	0	0	0	none
chr17:18377864..18377864+	chr17	+	18377864	18377864		1137728	NM_001104551	Vmn2r99	1	0	0	0	0	0	0	0	Simple_repeat
chr17:18390896..18390896+	chr17	+	18390896	18390896		1124696	NM_001104551	Vmn2r99	1	1	0	0	0	1	0	0	LINE

chr17:18398402..18398402+	chr17 +	18398402	18398402	0,169060117	1117190	NM_001104551	Vmn2r99	1	0	0	0	0	0	0	0	0	LTR
chr17:18419246..18419246-	chr17 -	18419246	18419246	0,193211562	4773	NM_001104543	Vmn2r94	1	0	0	0	0	0	0	0	1	LINE
chr17:18426302..18426302+	chr17 +	18426302	18426302	0,096605781	8942	NM_001104542	Vmn2r93	1	0	0	0	0	0	0	0	0	LINE
chr17:18467150..18467150+	chr17 +	18467150	18467150	0,241514452	93917	NM_001102581	Vmn2r95	1	0	0	0	0	0	0	0	0	LINE
chr17:18506180..18506180+	chr17 +	18506180	18506180	0,024151445	54887	NM_001102581	Vmn2r95	1	0	0	0	0	0	0	0	0	LTR
chr17:18509367..18509367+	chr17 +	18509367	18509367	0,024151445	51700	NM_001102581	Vmn2r95	1	1	0	0	0	0	0	0	0	Other
chr17:18509720..18509720+	chr17 +	18509720	18509720	0,072454336	51347	NM_001102581	Vmn2r95	1	1	0	0	0	0	0	0	0	Other
chr17:18514946..18514946+	chr17 +	18514946	18514946	0,024151445	46121	NM_001102581	Vmn2r95	1	0	0	0	0	0	0	0	0	LINE
chr17:18529691..18529691+	chr17 +	18529691	18529691	0,265665897	31376	NM_001102581	Vmn2r95	1	0	0	0	0	0	0	0	0	LINE
chr17:18533888..18533888+	chr17 +	18533888	18533888	0,024151445	27179	NM_001102581	Vmn2r95	1	0	0	0	0	0	0	0	0	LTR
chr17:18542928..18542928+	chr17 +	18542928	18542928	0,024151445	18139	NM_001102581	Vmn2r95	1	0	0	0	0	0	0	0	0	LTR
chr17:18548366..18548369+	chr17 +	18548366	18548369	0,072454336	12698	NM_001102581	Vmn2r95	1	0	0	0	0	0	0	0	0	LTR
chr17:18562999..18562999+	chr17 +	18562999	18562999	0,024151445	952593	NM_001104551	Vmn2r99	0	0	0	0	0	0	0	0	0	none
chr17:18568452..18568452+	chr17 +	18568452	18568452	0,024151445	947140	NM_001104551	Vmn2r99	0	1	0	0	0	0	0	0	0	none
chr17:18575233..18575233+	chr17 +	18575233	18575233	0,024151445	940359	NM_001104551	Vmn2r99	1	1	0	0	0	1	0	0	0	Simple_repeat
chr17:18593102..18593102+	chr17 +	18593102	18593102	0,024151445	126267	NM_001104547	Vmn2r96	1	0	0	0	0	0	0	0	0	LTR
chr17:18606216..18606216+	chr17 +	18606216	18606216	0,024151445	113153	NM_001104547	Vmn2r96	1	0	0	0	0	0	0	0	0	LINE
chr17:18607156..18607156+	chr17 +	18607156	18607156	0,217363007	112213	NM_001104547	Vmn2r96	1	0	0	0	0	0	0	0	0	LINE
chr17:18607498..18607498+	chr17 +	18607498	18607498	0,024151445	111871	NM_001104547	Vmn2r96	1	0	0	0	0	0	0	0	0	LINE
chr17:18607827..18607827+	chr17 +	18607827	18607827	0,024151445	111542	NM_001104547	Vmn2r96	1	0	0	0	0	0	0	0	0	LINE
chr17:18609387..18609387+	chr17 +	18609387	18609387	0,024151445	109982	NM_001104547	Vmn2r96	1	0	0	0	0	0	0	0	0	LTR
chr17:18612097..18612097+	chr17 +	18612097	18612097	0,169060117	107272	NM_001104547	Vmn2r96	1	0	0	0	0	0	0	0	0	LINE
chr17:18623997..18623997+	chr17 +	18623997	18623997	0,120757226	95372	NM_001104547	Vmn2r96	1	0	0	0	0	0	0	0	0	LINE
chr17:18628415..18628415+	chr17 +	18628415	18628415	0,265665897	90954	NM_001104547	Vmn2r96	1	1	0	0	0	0	0	0	0	LTR
chr17:18629346..18629347+	chr17 +	18629346	18629347	0,120757226	90023	NM_001104547	Vmn2r96	0	1	0	0	0	1	0	0	0	none
chr17:18629954..18629954+	chr17 +	18629954	18629954	0,072454336	89415	NM_001104547	Vmn2r96	0	1	0	0	0	1	0	0	0	none
chr17:18638413..18638413+	chr17 +	18638413	18638413	0,04830289	80956	NM_001104547	Vmn2r96	1	0	0	0	0	0	0	0	0	LTR
chr17:18643755..18643755+	chr17 +	18643755	18643755	0,024151445	75614	NM_001104547	Vmn2r96	1	0	0	0	0	0	0	0	0	LINE
chr17:18644478..18644478+	chr17 +	18644478	18644478	0,144908671	74891	NM_001104547	Vmn2r96	0	0	0	0	0	0	0	0	0	none
chr17:18645828..18645828+	chr17 +	18645828	18645828	0,024151445	73541	NM_001104547	Vmn2r96	1	0	0	0	0	0	0	0	0	LINE
chr17:18655411..18655411+	chr17 +	18655411	18655411	0,024151445	63958	NM_001104547	Vmn2r96	1	0	0	0	0	0	0	0	0	LINE
chr17:18659609..18659609+	chr17 +	18659609	18659609	0,024151445	59760	NM_001104547	Vmn2r96	1	0	0	0	0	0	0	0	0	LTR
chr17:18659680..18659682+	chr17 +	18659680	18659682	1,874755936	59687	NM_001104547	Vmn2r96	1	0	0	0	0	0	0	0	0	LTR
chr17:18662310..18662310+	chr17 +	18662310	18662310	0,04830289	57059	NM_001104547	Vmn2r96	1	0	0	0	0	0	0	0	0	LINE
chr17:18668869..18668869+	chr17 +	18668869	18668869	0,096605781	50500	NM_001104547	Vmn2r96	1	0	0	0	0	0	0	0	0	LINE
chr17:18730298..18730298+	chr17 +	18730298	18730298	0,217363007	785294	NM_001104551	Vmn2r99	0	0	0	0	0	0	0	0	0	none
chr17:18750135..18750135+	chr17 +	18750135	18750135	0,024151445	301150	NM_001104549	Vmn2r97	1	0	0	0	0	0	0	0	0	LTR
chr17:18750218..18750218+	chr17 +	18750218	18750218	0,024151445	301067	NM_001104549	Vmn2r97	1	0	0	0	0	0	0	0	0	LTR
chr17:18753197..18753197+	chr17 +	18753197	18753197	0,072454336	298088	NM_001104549	Vmn2r97	0	0	0	0	0	0	0	0	0	none
chr17:18795465..18795465+	chr17 +	18795465	18795465	0,024151445	255820	NM_001104549	Vmn2r97	1	0	0	0	0	0	0	0	0	LINE
chr17:18797353..18797353+	chr17 +	18797353	18797353	0,024151445	253932	NM_001104549	Vmn2r97	1	0	0	0	0	0	0	0	0	LINE
chr17:18828096..18828096+	chr17 +	18828096	18828096	0,024151445	223189	NM_001104549	Vmn2r97	1	0	0	0	0	0	0	0	0	LTR
chr17:18836250..18836250+	chr17 +	18836250	18836250	0,55548324	215035	NM_001104549	Vmn2r97	0	0	0	0	0	1	0	0	0	none
chr17:18849679..18849679+	chr17 +	18849679	18849679	0,024151445	201606	NM_001104549	Vmn2r97	1	0	0	0	0	0	0	0	0	LTR
chr17:18851181..18851181+	chr17 +	18851181	18851181	0,024151445	200104	NM_001104549	Vmn2r97	1	0	0	0	0	0	0	0	0	LTR
chr17:18852627..18852627+	chr17 +	18852627	18852627	0,024151445	198658	NM_001104549	Vmn2r97	1	0	0	0	0	0	0	0	0	LTR
chr17:18853266..18853266+	chr17 +	18853266	18853266	0,024151445	198019	NM_001104549	Vmn2r97	1	0	0	0	0	0	0	0	0	LTR
chr17:18853318..18853318+	chr17 +	18853318	18853318	0,04830289	197967	NM_001104549	Vmn2r97	1	0	0	0	0	0	0	0	0	LTR
chr17:18854618..18854618+	chr17 +	18854618	18854618	0,024151445	196667	NM_001104549	Vmn2r97	0	0	0	0	0	0	0	0	0	none
chr17:18889971..18889971+	chr17 +	18889971	18889971	0,024151445	161314	NM_001104549	Vmn2r97	0	0	0	0	0	0	0	0	0	none
chr17:18900147..18900147+	chr17 +	18900147	18900147	0,313968788	151138	NM_001104549	Vmn2r97	0	1	0	0	0	0	0	0	0	none
chr17:18952376..18952376+	chr17 +	18952376	18952376	0,024151445	98909	NM_001104549	Vmn2r97	0	1	0	0	0	1	0	0	0	none
chr17:18963717..18963717+	chr17 +	18963717	18963717	0,120757226	87568	NM_001104549	Vmn2r97	1	0	0	0	0	0	1	0	0	LINE
chr17:18975316..18975316+	chr17 +	18975316	18975316	0,024151445	75969	NM_001104549	Vmn2r97	0	1	0	0	0	0	1	1	0	none
chr17:18977334..18977334+	chr17 +	18977334	18977334	0,024151445	73951	NM_001104549	Vmn2r97	0	0	0	0	0	0	1	1	0	none
chr17:18977658..18977658+	chr17 +	18977658	18977658	0,072454336	73627	NM_001104549	Vmn2r97	0	0	0	0	0	0	1	1	0	none
chr17:18992740..18992740+	chr17 +	18992740	18992740	0,024151445	58545	NM_001104549	Vmn2r97	1	0	0	0	0	0	0	0	0	LTR
chr17:19038612..19038612+	chr17 +	19038612	19038612	0,024151445	12673	NM_001104549	Vmn2r97	1	0	0	0	0	0	0	0	0	LTR
chr17:19049982..19049985+	chr17 +	19049982	19049985	0,193211562	1303	NM_001104549	Vmn2r97	1	1	0	0	0	0	0	0	1	LTR
chr17:19055357..19055357+	chr17 +	19055357	19055357	0,024151445	460235	NM_001104551	Vmn2r99	0	0	0	0	0	0	0	0	0	none
chr17:19088897..19088897+	chr17 +	19088897	19088897	0,024151445	101559	NM_001104550	Vmn2r98	0	0	0	0	0	0	1	0	0	none
chr17:19095828..19095828+	chr17 +	19095828	19095828	0,144908671	94628	NM_001104550	Vmn2r98	0	0	0	0	0	0	0	0	0	none
chr17:19097537..19097537+	chr17 +	19097537	19097537	0,169060117	92919	NM_001104550	Vmn2r98	1	0	0	0	0	0	1	0	0	LTR
chr17:19159299..19159299+	chr17 +	19159299	19159299	0,024151445	31157	NM_001104550	Vmn2r98	1	0	0	0	0	0	0	0	0	LTR
chr17:19174634..19174634+	chr17 +	19174634	19174634	0,120757226	15822	NM_001104550	Vmn2r98	1	0	0	0	0	0	0	0	0	LTR
chr17:19196809..19196809+	chr17 +	19196809	19196809	0,193211562	318783	NM_001104551	Vmn2r99	0	1	0	0	0	0	0	0	0	none
chr17:19231807..19231807+	chr17 +	19231807	19231807	0,024151445	283785	NM_001104551	Vmn2r99	1	0	0	0	0	0	0	0	0	LINE
chr17:19234499..19234499+	chr17 +	19234499	19234499	0,04830289	281093	NM_001104551	Vmn2r99	0	0	0	0	0	0	0	0	0	none
chr17:19237681..19237681+	chr17 +	19237681	19237681	0,096605781	277911	NM_001104551	Vmn2r99	1	0	0	0	0	0	0	0	0	LINE
chr17:19237904..19237904+	chr17 +	1923790															

chr17:19322690..19322690+	chr17 +	19322690	19322690		0,024151445	192902	NM_001104551	Vmn2r99	1	0	0	0	0	0	0	0	0	LINE
chr17:19323450..19323450+	chr17 +	19323450	19323450		0,024151445	192142	NM_001104551	Vmn2r99	0	0	0	0	0	0	0	0	0	LINE
chr17:19323473..19323473+	chr17 +	19323473	19323473		0,024151445	192119	NM_001104551	Vmn2r99	1	0	0	0	0	0	0	0	0	LINE
chr17:19327873..19327873+	chr17 +	19327873	19327873		0,096605781	187719	NM_001104551	Vmn2r99	1	0	0	0	0	0	0	0	0	LTR
chr17:19330760..19330760+	chr17 +	19330760	19330760		0,096605781	184832	NM_001104551	Vmn2r99	1	0	0	0	0	0	0	0	0	LTR
chr17:19336523..19336524+	chr17 +	19336523	19336524		0,04830289	179069	NM_001104551	Vmn2r99	0	0	0	0	0	0	0	0	0	none
chr17:19346888..19346888+	chr17 +	19346888	19346888		0,024151445	168704	NM_001104551	Vmn2r99	1	0	0	0	0	0	0	0	0	LTR
chr17:19354790..19354790+	chr17 +	19354790	19354790		0,024151445	160802	NM_001104551	Vmn2r99	0	1	0	0	0	0	0	0	0	none
chr17:19359686..19359686+	chr17 +	19359686	19359686		0,024151445	155906	NM_001104551	Vmn2r99	1	0	0	0	0	0	0	0	0	LTR
chr17:19360928..19360928+	chr17 +	19360928	19360928		0,024151445	154664	NM_001104551	Vmn2r99	0	0	0	0	0	0	0	0	0	none
chr17:19374752..19374752+	chr17 +	19374752	19374752		0,024151445	140840	NM_001104551	Vmn2r99	1	0	0	0	0	0	0	0	0	LINE
chr17:19383633..19383633+	chr17 +	19383633	19383633		0,024151445	131959	NM_001104551	Vmn2r99	0	0	0	0	0	0	0	0	0	none
chr17:19384923..19384923+	chr17 +	19384923	19384923		0,072454336	130669	NM_001104551	Vmn2r99	1	0	0	0	0	0	0	0	0	LINE
chr17:19415030..19415030+	chr17 +	19415030	19415030		0,024151445	100562	NM_001104551	Vmn2r99	0	0	0	0	0	0	1	1	0	none
chr17:19426901..19426902+	chr17 +	19426901	19426902		0,04830289	88691	NM_001104551	Vmn2r99	0	1	0	0	0	1	0	0	0	none
chr17:19447338..19447338+	chr17 +	19447338	19447338		0,024151445	68254	NM_001104551	Vmn2r99	1	0	0	0	0	0	0	0	0	LINE
chr17:19461155..19461155+	chr17 +	19461155	19461155		0,072454336	54437	NM_001104551	Vmn2r99	1	0	0	0	0	0	0	0	0	LTR
chr17:19495617..19495617+	chr17 +	19495617	19495617		0,265665897	19975	NM_001104551	Vmn2r99	1	0	0	0	0	0	0	0	0	LINE
chr17:19513856..19513856+	chr17 +	19513856	19513856		0,024151445	1736	NM_001104551	Vmn2r99	1	0	0	0	0	0	0	0	0	LINE
chr17:19541594..19541594+	chr17 +	19541594	19541594		0,144908671	100180	NM_001104562	Vmn2r100	1	0	0	0	0	0	0	0	0	LINE
chr17:19554429..19554429+	chr17 +	19554429	19554429		0,144908671	87345	NM_001104562	Vmn2r100	1	0	0	0	0	0	0	0	0	LINE
chr17:19554522..19554522+	chr17 +	19554522	19554522		0,024151445	87252	NM_001104562	Vmn2r100	1	0	0	0	0	0	0	0	0	LINE
chr17:19557635..19557635+	chr17 +	19557635	19557635		0,072454336	84139	NM_001104562	Vmn2r100	1	0	0	0	0	0	0	0	0	LTR
chr17:19559794..19559794+	chr17 +	19559794	19559794		0,120757226	81980	NM_001104562	Vmn2r100	1	0	0	0	0	0	1	0	0	LINE
chr17:19563231..19563231+	chr17 +	19563231	19563231		0,024151445	78543	NM_001104562	Vmn2r100	1	0	0	0	0	0	0	0	0	LINE
chr17:19582714..19582719+	chr17 +	19582714	19582719		0,04830289	59060	NM_001104562	Vmn2r100	1	0	0	0	0	0	0	0	0	LTR
chr17:19584811..19584811+	chr17 +	19584811	19584811		0,096605781	56963	NM_001104562	Vmn2r100	1	0	0	0	0	0	1	0	0	none
chr17:19602470..19602470+	chr17 +	19602470	19602470		0,217363007	39304	NM_001104562	Vmn2r100	1	0	0	0	0	0	0	0	0	LINE
chr17:19602715..19602715+	chr17 +	19602715	19602715		0,144908671	39059	NM_001104562	Vmn2r100	1	0	0	0	0	0	0	0	0	LINE
chr17:19606334..19606334+	chr17 +	19606334	19606334		0,289817343	35440	NM_001104562	Vmn2r100	0	0	0	0	0	0	0	0	0	none
chr17:19623148..19623148+	chr17 +	19623148	19623148		0,024151445	18626	NM_001104562	Vmn2r100	1	0	0	0	0	0	0	0	0	LTR
chr17:19628512..19628512+	chr17 +	19628512	19628512		0,072454336	13262	NM_001104562	Vmn2r100	1	0	0	0	0	0	0	0	0	LINE
chr17:19669922..19669922+	chr17 +	19669922	19669922		0,024151445	44272	NM_001104563	Vmn2r101	0	0	0	0	0	0	0	0	0	none
chr17:19670066..19670066+	chr17 +	19670066	19670066		0,144908671	44128	NM_001104563	Vmn2r101	0	0	0	0	0	0	0	0	0	none
chr17:19675621..19675621+	chr17 +	19675621	19675621		0,144908671	38573	NM_001104563	Vmn2r101	1	0	0	0	0	0	0	0	0	LTR
chr17:19692599..19692599+	chr17 +	19692599	19692599		0,024151445	21595	NM_001104563	Vmn2r101	1	0	0	0	0	0	0	0	0	LTR
chr17:19698437..19698437+	chr17 +	19698437	19698437		0,531331795	15757	NM_001104563	Vmn2r101	1	0	0	0	0	0	0	0	0	LTR
chr17:19703464..19703464+	chr17 +	19703464	19703464		0,024151445	10730	NM_001104563	Vmn2r101	1	0	0	0	0	0	0	0	0	LINE
chr17:19710999..19710999+	chr17 +	19710999	19710999		0,024151445	3195	NM_001104563	Vmn2r101	0	1	0	0	0	0	0	0	0	none
chr17:19794193..19794193+	chr17 +	19794193	19794193		0,120757226	3169	NM_001104564	Vmn2r102	0	1	0	0	0	0	0	0	0	none
chr17:19834965..19834965+	chr17 +	19834965	19834965		0,169060117	75361	NM_001104565	Vmn2r103	0	0	0	0	0	0	0	0	0	none
chr17:19850436..19850436+	chr17 +	19850436	19850436		0,024151445	59890	NM_001104565	Vmn2r103	1	0	0	0	0	0	0	0	0	LINE
chr17:19851389..19851389+	chr17 +	19851389	19851389		0,096605781	58937	NM_001104565	Vmn2r103	1	0	0	0	0	0	0	0	0	LINE
chr17:19861587..19861587+	chr17 +	19861587	19861587		0,169060117	48739	NM_001104565	Vmn2r103	1	0	0	0	0	0	0	0	0	none
chr17:19881752..19881752+	chr17 +	19881752	19881752		0,024151445	28574	NM_001104565	Vmn2r103	0	1	0	0	0	0	0	0	0	none
chr17:19882785..19882785+	chr17 +	19882785	19882785		0,024151445	27541	NM_001104565	Vmn2r103	1	0	0	0	0	0	0	0	0	LINE
chr17:19882980..19882980+	chr17 +	19882980	19882980		0,024151445	27346	NM_001104565	Vmn2r103	1	0	0	0	0	0	0	0	0	LINE
chr17:19889400..19889400+	chr17 +	19889400	19889400		0,024151445	20926	NM_001104565	Vmn2r103	1	0	0	0	0	0	0	0	0	LINE
chr17:19889706..19889706+	chr17 +	19889706	19889706		0,024151445	20620	NM_001104565	Vmn2r103	1	0	0	0	0	0	0	0	0	LINE
chr17:19891023..19891023+	chr17 +	19891023	19891023		0,024151445	19303	NM_001104565	Vmn2r103	1	0	0	0	0	0	0	0	0	LTR
chr17:19899820..19899820+	chr17 +	19899820	19899820		0,024151445	10506	NM_001104565	Vmn2r103	0	0	0	0	0	0	0	0	0	none
chr17:19905654..19905654+	chr17 +	19905654	19905654		0,169060117	4672	NM_001104565	Vmn2r103	1	1	0	0	0	0	0	0	1	LTR
chr17:20186280..20186280+	chr17 -	20186280	20186280		0,011146821	610	NM_001104566	Vmn2r104	0	0	0	0	0	0	1	1	1	none
chr17:20186364..20186364+	chr17 -	20186364	20186364		0,04830289	694	NM_001104566	Vmn2r104	0	0	0	0	0	0	1	1	1	none
chr17:20218194..20218194+	chr17 -	20218194	20218194		0,096605781	32524	NM_001104566	Vmn2r104	0	0	0	0	0	0	1	1	0	none
chr17:20251738..20251738+	chr17 -	20251738	20251738		0,024151445	66068	NM_001104566	Vmn2r104	0	1	0	0	0	0	0	0	0	none
chr17:20255068..20255068+	chr17 -	20255068	20255068		0,024151445	69398	NM_001104566	Vmn2r104	1	0	0	0	0	0	0	0	0	LTR
chr17:20266119..20266119+	chr17 -	20266119	20266119		0,024151445	80449	NM_001104566	Vmn2r104	0	1	0	0	0	0	0	0	0	none
chr17:20268478..20268478+	chr17 -	20268478	20268478		0,241514452	82808	NM_001104566	Vmn2r104	0	0	0	0	0	0	1	0	0	none
chr17:20272960..20272960+	chr17 -	20272960	20272960		0,024151445	87290	NM_001104566	Vmn2r104	0	0	0	0	0	0	0	0	0	LINE
chr17:20278527..20278527+	chr17 -	20278527	20278527		0,072454336	92857	NM_001104566	Vmn2r104	0	0	0	0	0	0	1	1	0	none
chr17:20291675..20291675+	chr17 -	20291675	20291675		0,04830289	106005	NM_001104566	Vmn2r104	0	0	0	0	0	0	0	0	0	none
chr17:20296801..20296801+	chr17 -	20296801	20296801		0,04830289	111131	NM_001104566	Vmn2r104	1	0	0	0	0	0	0	0	0	LINE
chr17:20297645..20297645+	chr17 -	20297645	20297645		0,024151445	111975	NM_001104566	Vmn2r104	1	0	0	0	0	0	0	0	0	LINE
chr17:20298248..20298248+	chr17 -	20298248	20298248		0,024151445	112578	NM_001104566	Vmn2r104	1	0	0	0	0	0	0	0	0	SINE
chr17:20298474..20298474+	chr17 -	20298474	20298474		0,04830289	112804	NM_001104566	Vmn2r104	1	0	0	0	0	0	0	0	0	none
chr17:20301796..20301796+	chr17 -	20301796	20301796		0,04830289	116126	NM_001104566	Vmn2r104	0	0	0	0	0	0	0	0	0	LINE
chr17:20307074..20307074+	chr17 -	20307074	20307074		0,024151445	121404	NM_001104566	Vmn2r104	0	0	0	0	0	0	1	1	0	none
chr17:20310467..20																		

chr17:20454131..20454131-	chr17 -	20454131	20454131	0,313968788	31236	NM_001104568	Vmn2r106	0	0	0	0	0	0	0	0	0	none
chr17:20457766..20457766-	chr17 -	20457766	20457766	0,024151445	34871	NM_001104568	Vmn2r106	0	0	0	0	0	0	0	0	0	none
chr17:20458634..20458634-	chr17 -	20458634	20458634	0,144908671	35739	NM_001104568	Vmn2r106	0	0	0	0	0	0	0	0	0	none
chr17:20463360..20463360-	chr17 -	20463360	20463360	0,169060117	40465	NM_001104568	Vmn2r106	1	0	0	0	0	0	0	0	0	LINE
chr17:20477154..20477154-	chr17 -	20477154	20477154	0,04830289	54259	NM_001104568	Vmn2r106	1	0	0	0	0	0	1	0	0	LINE
chr17:20478869..20478869+	chr17 +	20478869	20478869	0,072454336	3519	NM_001104569	Vmn2r107	1	0	0	0	0	0	0	0	0	LINE
chr17:20479865..20479865+	chr17 +	20479865	20479865	0,024151445	2523	NM_001104569	Vmn2r107	1	0	0	0	0	0	0	0	0	LINE
chr17:20481013..20481013-	chr17 -	20481013	20481013	0,024151445	58118	NM_001104568	Vmn2r106	1	0	0	0	0	0	0	0	0	LTR
chr17:20626823..20626823-	chr17 -	20626823	20626823	0,024151445	8122	NM_001104570	Vmn2r108	0	0	0	0	0	0	0	0	0	none
chr17:20635405..20635405-	chr17 -	20635405	20635405	0,169060117	16704	NM_001104570	Vmn2r108	0	1	0	0	0	0	1	1	0	none
chr17:20636667..20636667-	chr17 -	20636667	20636667	0,024151445	17966	NM_001104570	Vmn2r108	1	1	0	0	0	0	1	0	0	Simple_repeat
chr17:20704275..20704275-	chr17 -	20704275	20704275	0,024151445	2054	NM_001104571	Vmn2r109	1	0	0	0	0	0	0	0	0	LINE
chr17:20705245..20705245-	chr17 -	20705245	20705245	0,024151445	3024	NM_001104571	Vmn2r109	0	0	0	0	0	0	0	0	1	none
chr17:20710363..20710363-	chr17 -	20710363	20710363	0,024151445	8142	NM_001104571	Vmn2r109	0	1	0	0	0	0	1	1	1	LTR
chr17:20743562..20743562-	chr17 -	20743562	20743562	0,04830289	9838	NM_001104572	Vmn2r110	1	0	0	0	0	0	1	0	0	LTR
chr17:20749544..20749544-	chr17 -	20749544	20749544	0,024151445	15820	NM_001104572	Vmn2r110	1	0	0	0	0	0	0	0	0	LINE
chr17:20753276..20753276-	chr17 -	20753276	20753276	0,072454336	19552	NM_001104572	Vmn2r110	1	0	0	0	0	0	0	0	0	LINE
chr17:20753778..20753778-	chr17 -	20753778	20753778	0,024151445	20054	NM_001104572	Vmn2r110	1	0	0	0	0	0	0	0	0	LINE
chr17:20756163..20756163-	chr17 -	20756163	20756163	0,024151445	22439	NM_001104572	Vmn2r110	1	0	0	0	0	0	0	0	0	LTR
chr17:20761970..20761970-	chr17 -	20761970	20761970	0,024151445	28246	NM_001104572	Vmn2r110	0	1	0	0	0	0	0	0	0	none
chr17:20783166..20783166-	chr17 -	20783166	20783166	0,077821324	49442	NM_001104572	Vmn2r110	0	1	0	0	0	0	0	0	0	none
chr17:20787242..20787242-	chr17 -	20787242	20787242	0,024151445	53518	NM_001104572	Vmn2r110	1	1	0	0	0	0	0	0	0	LINE
chr17:20787381..20787381-	chr17 -	20787381	20787381	0,024151445	53657	NM_001104572	Vmn2r110	1	1	0	0	0	0	0	0	0	LINE
chr17:20788852..20788852-	chr17 -	20788852	20788852	0,120757226	55128	NM_001104572	Vmn2r110	0	0	0	0	0	0	0	0	0	none
chr17:20792168..20792168-	chr17 -	20792168	20792168	0,024151445	58444	NM_001104572	Vmn2r110	1	0	0	0	0	0	0	0	0	LINE
chr17:20803127..20803127-	chr17 -	20803127	20803127	0,169060117	69403	NM_001104572	Vmn2r110	0	0	0	0	0	0	0	0	0	none
chr17:20810975..20810975-	chr17 -	20810975	20810975	0,169060117	77251	NM_001104572	Vmn2r110	1	0	0	0	0	0	0	0	0	LTR
chr17:20811780..20811780-	chr17 -	20811780	20811780	0,096605781	78056	NM_001104572	Vmn2r110	0	0	0	0	0	0	0	0	0	none
chr17:22714215..22714215+	chr17 +	22714215	22714215	0,410574569	23899	NM_001104575	Vmn2r112	0	0	0	0	0	0	0	0	0	none
chr17:22716778..22716778+	chr17 +	22716778	22716778	0,120757226	21336	NM_001104575	Vmn2r112	1	0	0	0	0	0	0	0	0	Other
chr17:22717664..22717664+	chr17 +	22717664	22717664	0,024151445	20450	NM_001104575	Vmn2r112	1	0	0	0	0	0	0	0	0	LINE
chr17:22721395..22721411+	chr17 +	22721395	22721411	0,04830289	16719	NM_001104575	Vmn2r112	1	0	0	0	0	0	0	0	0	LINE
chr17:22722080..22722083-	chr17 -	22722080	22722083	0,265665897	11339	NM_001104573	Vmn2r111	0	0	0	0	0	0	0	0	0	none
chr17:22728707..22728707+	chr17 +	22728707	22728707	0,024151445	17966	NM_001104573	Vmn2r111	1	0	0	0	0	0	0	0	0	Low_complexity
chr17:22731306..22731306+	chr17 +	22731306	22731306	0,144908671	6808	NM_001104575	Vmn2r112	1	0	0	0	0	0	0	0	0	LINE
chr17:23006234..23006235+	chr17 +	23006234	23006235	0,410574569	73916	NM_001104578	Vmn2r113	1	1	0	0	0	0	0	0	0	LTR
chr17:23009618..23009618+	chr17 +	23009618	23009618	0,024151445	70532	NM_001104578	Vmn2r113	0	0	0	0	0	0	0	0	0	none
chr17:23010743..23010743+	chr17 +	23010743	23010743	0,072454336	69407	NM_001104578	Vmn2r113	0	1	0	0	0	0	1	1	0	none
chr17:23010933..23010933+	chr17 +	23010933	23010933	0,024151445	69217	NM_001104578	Vmn2r113	0	1	0	0	0	0	1	1	0	none
chr17:23010968..23010968+	chr17 +	23010968	23010968	0,024151445	69182	NM_001104578	Vmn2r113	0	1	0	0	0	0	1	1	0	none
chr17:23011788..23011788+	chr17 +	23011788	23011788	0,024151445	68362	NM_001104578	Vmn2r113	0	1	0	0	0	0	1	0	0	none
chr17:23013236..23013236+	chr17 +	23013236	23013236	0,024151445	66914	NM_001104578	Vmn2r113	1	0	0	0	0	0	0	0	0	rRNA
chr17:23014353..23014361+	chr17 +	23014353	23014361	0,241514452	65789	NM_001104578	Vmn2r113	1	0	0	0	0	0	0	0	0	rRNA
chr17:23014431..23014436+	chr17 +	23014431	23014436	0,120757226	65717	NM_001104578	Vmn2r113	1	0	0	0	0	0	0	0	0	rRNA
chr17:23014564..23014567+	chr17 +	23014564	23014567	0,072454336	65583	NM_001104578	Vmn2r113	1	0	0	0	0	0	0	0	0	rRNA
chr17:23014607..23014607+	chr17 +	23014607	23014607	0,003018931	65543	NM_001104578	Vmn2r113	1	0	0	0	0	0	0	0	0	rRNA
chr17:23014865..23014874+	chr17 +	23014865	23014874	0,796997692	65283	NM_001104578	Vmn2r113	1	0	0	0	0	0	0	0	0	rRNA
chr17:23014933..23014933+	chr17 +	23014933	23014933	0,386423124	65217	NM_001104578	Vmn2r113	1	0	0	0	0	0	1	0	0	rRNA
chr17:23014961..23014970+	chr17 +	23014961	23014970	0,096605781	65180	NM_001104578	Vmn2r113	1	0	0	0	0	0	1	0	0	rRNA
chr17:23015034..23015042+	chr17 +	23015034	23015042	0,362271678	65114	NM_001104578	Vmn2r113	1	0	0	0	0	0	1	0	0	rRNA
chr17:23015071..23015089+	chr17 +	23015071	23015089	0,193211562	65079	NM_001104578	Vmn2r113	1	0	0	0	0	0	1	0	0	rRNA
chr17:23015127..23015135+	chr17 +	23015127	23015135	0,072454336	65023	NM_001104578	Vmn2r113	1	0	0	0	0	0	1	0	0	rRNA
chr17:23018595..23018598+	chr17 +	23018595	23018598	0,096605781	61555	NM_001104578	Vmn2r113	1	1	0	0	0	0	0	0	0	LTR
chr17:23018991..23018991+	chr17 +	23018991	23018991	0,096605781	61159	NM_001104578	Vmn2r113	0	1	0	0	0	0	0	0	0	none
chr17:23032144..23032144+	chr17 +	23032144	23032144	0,338120233	48006	NM_001104578	Vmn2r113	0	0	0	0	0	0	1	1	0	none
chr17:23075604..23075604+	chr17 +	23075604	23075604	0,024151445	4546	NM_001104578	Vmn2r113	1	0	0	0	0	0	0	0	0	LTR
chr17:23466321..23466321+	chr17 +	23466321	23466321	0,096605781	14622	NM_001104579	Vmn2r115	1	0	0	0	0	0	0	0	0	LINE
chr17:23467931..23467931+	chr17 +	23467931	23467931	0,265665897	18150	NM_001102584	Vmn2r114	1	0	0	0	0	0	0	0	0	LINE
chr17:23478667..23478667+	chr17 +	23478667	23478667	0,024151445	2276	NM_001104579	Vmn2r115	0	0	0	0	0	1	0	0	1	none
chr17:23501990..23501990+	chr17 +	23501990	23501990	0,024151445	19779	NM_001104580	Vmn2r116	1	0	0	0	0	0	0	0	0	LINE
chr17:23617293..23617293+	chr17 +	23617293	23617293	0,169060117	228	NM_001104581	Vmn2r117	0	0	0	0	0	0	0	0	1	none
chr17:23619064..23619064+	chr17 +	23619064	23619064	0,217363007	1999	NM_001104581	Vmn2r117	1	0	0	0	0	0	0	0	0	LINE
chr17:23626926..23626926+	chr17 +	23626926	23626926	0,024151445	9861	NM_001104581	Vmn2r117	0	0	0	0	0	0	0	0	1	none
chr17:23631542..23631542+	chr17 +	23631542	23631542	0,04830289	14477	NM_001104581	Vmn2r117	1	0	0	0	0	0	0	0	0	LINE
chr17:23656356..23656356+	chr17 +	23656356	23656356	0,241514452	39291	NM_001104581	Vmn2r117	1	0	0	0	0	0	0	0	0	Other
chr17:23656555..23656555+	chr17 +	23656555	23656555	0,024151445	39490	NM_001104581	Vmn2r117	1	0	0	0	0	0	0	0	0	LINE
chr17:23677153..23677153+	chr17 +	23677153	23677153	0,04830289	60088	NM_001104581	Vmn2r117	0	1	0	0	0	0	0	0	0	none
chr17:23679697..23679697+	chr17 +	23679697	23679697	0,120757226	62632	NM_001104581	Vmn2r117	0	1	0	0	0	0	0	0	0	none
chr17:23680396..23680396+	chr17 +	23680396	23680396	0,024151445	63331	NM_001104581	Vmn2r117	1	1	0	0	0	0	0	0	0	LTR
chr17:23681874..23681874+	chr17 +	23681874	23681874	0,217363007	64809	NM_001104581	Vmn2r117										

chr17:55785066..55785066-	chr17 -	55785066	55785066	0,04830289	20595	NM_001104582	Vmn2r118	0	1	0	0	0	0	0	0	none
chr17:55789138..55789138-	chr17 -	55789138	55789138	0,024151445	24667	NM_001104582	Vmn2r118	0	0	0	0	0	0	0	0	none
chr17:55789955..55789955-	chr17 -	55789955	55789955	0,024151445	25484	NM_001104582	Vmn2r118	1	0	0	0	0	0	0	0	LINE
chr17:55789997..55789998-	chr17 -	55789997	55789998	0,265665897	25527	NM_001104582	Vmn2r118	1	0	0	0	0	0	0	0	LINE
chr17:55790097..55790097-	chr17 -	55790097	55790097	0,144908671	25626	NM_001104582	Vmn2r118	1	0	0	0	0	0	0	0	LINE
chr17:55790346..55790346-	chr17 -	55790346	55790346	0,120757226	25875	NM_001104582	Vmn2r118	0	0	0	0	0	0	0	0	none
chr17:55790635..55790635-	chr17 -	55790635	55790635	0,024151445	26164	NM_001104582	Vmn2r118	0	1	0	0	0	0	0	0	none
chr17:55790668..55790668-	chr17 -	55790668	55790668	0,024151445	26197	NM_001104582	Vmn2r118	0	1	0	0	0	0	0	0	none
chr17:55790718..55790718-	chr17 -	55790718	55790718	0,024151445	26247	NM_001104582	Vmn2r118	0	1	0	0	0	0	0	0	none
chr17:55790766..55790766-	chr17 -	55790766	55790766	0,072454336	26295	NM_001104582	Vmn2r118	1	1	0	0	0	0	0	0	LINE
chr17:55790799..55790807-	chr17 -	55790799	55790807	0,04830289	26328	NM_001104582	Vmn2r118	1	1	0	0	0	0	0	0	LINE
chr17:55791253..55791276-	chr17 -	55791253	55791276	0,241514452	26782	NM_001104582	Vmn2r118	1	0	0	0	0	0	0	0	LINE
chr17:57704868..57704868-	chr17 -	57704868	57704868	0,024151445	1917	NM_001104591	Vmn2r120	1	0	0	0	0	0	1	1	0
chr17:57722855..57722855-	chr17 -	57722855	57722855	0,169060117	19630	NM_001104591	Vmn2r120	1	0	0	0	0	0	0	0	LINE
chr17:57723554..57723554-	chr17 -	57723554	57723554	0,072454336	37617	NM_001104591	Vmn2r120	1	0	0	0	0	0	0	0	LINE
chr17:57724918..57724918-	chr17 -	57724918	57724918	0,072454336	38316	NM_001104591	Vmn2r120	1	0	0	0	0	0	0	0	LINE
chr17:57729324..57729324-	chr17 -	57729324	57729324	0,04830289	39680	NM_001104591	Vmn2r120	1	0	0	0	0	0	0	0	LTR
chr17:57730616..57730619-	chr17 -	57730616	57730619	0,169060117	44086	NM_001104591	Vmn2r120	1	0	0	0	0	0	0	0	LINE
chr17:57737849..57737849-	chr17 -	57737849	57737849	0,024151445	45381	NM_001104591	Vmn2r120	1	0	0	0	0	0	0	0	LINE
chr17:57740956..57740956-	chr17 -	57740956	57740956	0,024151445	52611	NM_001104591	Vmn2r120	1	0	0	0	0	0	0	0	LINE
chr17:57761178..57761178-	chr17 -	57761178	57761178	0,193211562	55718	NM_001104591	Vmn2r120	1	0	0	0	0	0	0	0	none
chr17:57766121..57766121-	chr17 -	57766121	57766121	0,072454336	75940	NM_001104591	Vmn2r120	1	0	0	0	0	0	0	0	none
chr17:57766986..57766986-	chr17 -	57766986	57766986	0,04830289	80883	NM_001104591	Vmn2r120	1	0	0	0	0	0	0	0	none
chr17:57768780..57768780-	chr17 -	57768780	57768780	0,024151445	81748	NM_001104591	Vmn2r120	0	0	0	0	0	0	0	0	none
chr17:57781040..57781040-	chr17 -	57781040	57781040	0,04830289	83542	NM_001104591	Vmn2r120	0	0	0	0	0	0	0	0	none
chr17:57787952..57787952-	chr17 -	57787952	57787952	0,04830289	95802	NM_001104591	Vmn2r120	1	0	0	0	0	0	0	0	LTR
chr17:57812416..57812416-	chr17 -	57812416	57812416	0,024151445	102714	NM_001104591	Vmn2r120	0	0	0	0	0	0	1	0	none
chr17:57812463..57812463-	chr17 -	57812463	57812463	0,024151445	127178	NM_001104591	Vmn2r120	0	1	0	0	0	0	0	0	none
chr17:57822505..57822505-	chr17 -	57822505	57822505	0,024151445	127225	NM_001104591	Vmn2r120	0	1	0	0	0	0	0	0	none
chr17:57835923..57835927-	chr17 -	57835923	57835927	0,024151445	127225	NM_001104591	Vmn2r120	0	1	0	0	0	0	0	0	none
chr17:57836663..57836663-	chr17 -	57836663	57836663	0,024151445	137267	NM_001104591	Vmn2r120	1	1	0	0	0	0	0	0	SINE
chr17:57849985..57849985-	chr17 -	57849985	57849985	0,024151445	150689	NM_001104591	Vmn2r120	0	0	0	0	0	0	0	0	none
chr17:57852942..57852942-	chr17 -	57852942	57852942	0,024151445	151425	NM_001104591	Vmn2r120	1	0	0	0	0	0	0	0	none
chr17:57855655..57855655-	chr17 -	57855655	57855655	0,024151445	164747	NM_001104591	Vmn2r120	1	0	0	0	0	0	0	0	LINE
chr17:57857984..57857984-	chr17 -	57857984	57857984	0,096605781	167704	NM_001104591	Vmn2r120	1	0	0	0	0	0	0	0	LINE
chr17:57905147..57905147-	chr17 -	57905147	57905147	0,024151445	170417	NM_001104591	Vmn2r120	1	0	0	0	0	0	0	0	LINE
chr3:63823105..63823114+	chr3 +	63823105	63823114	0,120757226	172746	NM_001104591	Vmn2r120	1	1	0	0	0	0	0	0	LTR
chr3:63823165..63823165+	chr3 +	63823165	63823165	0,217363007	219909	NM_001104591	Vmn2r120	1	1	0	1	1	0	0	1	LINE
chr3:63823232..63823232+	chr3 +	63823232	63823232	0,04830289	62449	NM_001104591	Vmn2r120	0	1	1	1	1	0	0	0	none
chr3:63823269..63823276+	chr3 +	63823269	63823276	0,845300583	62398	NM_001104591	Vmn2r120	0	1	1	1	1	0	0	0	none
chr3:63823300..63823313+	chr3 +	63823300	63823313	0,386423124	62331	NM_001104591	Vmn2r120	0	1	1	1	1	0	0	0	none
chr3:63823364..63823384+	chr3 +	63823364	63823384	0,313968788	62294	NM_001104591	Vmn2r120	0	1	1	1	1	0	0	0	none
chr3:63823411..63823411+	chr3 +	63823411	63823411	0,024151445	62179	NM_001104591	Vmn2r120	0	1	1	1	1	0	0	0	none
chr3:63823444..63823444+	chr3 +	63823444	63823444	0,120757226	62152	NM_001104591	Vmn2r120	0	1	1	1	1	0	0	0	none
chr3:63823501..63823549+	chr3 +	63823501	63823549	0,917754919	62119	NM_001104591	Vmn2r120	0	1	1	1	1	0	0	0	none
chr3:63823574..63823574+	chr3 +	63823574	63823574	0,024151445	62045	NM_001104591	Vmn2r120	1	1	1	1	1	0	0	0	SINE
chr3:63823603..63823603+	chr3 +	63823603	63823603	0,024151445	61989	NM_001104591	Vmn2r120	0	1	1	1	1	0	0	0	none
chr3:63823897..63823897+	chr3 +	63823897	63823897	0,04830289	61960	NM_001104591	Vmn2r120	1	1	1	1	1	0	0	0	none
chr3:63823986..63823986+	chr3 +	63823986	63823986	0,144908671	61666	NM_001104591	Vmn2r120	0	1	1	1	1	0	0	0	none
chr3:63824243..63824243+	chr3 +	63824243	63824243	0,169060117	61577	NM_001104591	Vmn2r120	0	1	1	1	1	0	0	0	none
chr3:63824298..63824298+	chr3 +	63824298	63824298	0,289817343	61320	NM_001104591	Vmn2r120	0	1	1	1	1	0	0	0	none
chr3:63824366..63824366+	chr3 +	63824366	63824366	0,024151445	61265	NM_001104591	Vmn2r120	0	1	1	1	1	0	0	0	none
chr3:63824390..63824390+	chr3 +	63824390	63824390	0,096605781	61197	NM_001104591	Vmn2r120	0	1	1	1	1	0	0	0	none
chr3:63824586..63824586+	chr3 +	63824586	63824586	0,241514452	61173	NM_001104591	Vmn2r120	0	1	1	1	1	0	0	0	none
chr3:63824927..63824930+	chr3 +	63824927	63824930	1,0143607	60977	NM_001104591	Vmn2r120	0	1	1	1	1	0	0	0	none
chr3:63824978..63824978+	chr3 +	63824978	63824978	0,024151445	60636	NM_001104591	Vmn2r120	0	1	1	1	1	0	0	0	none
chr3:63825052..63825052+	chr3 +	63825052	63825052	0,024151445	60585	NM_001104591	Vmn2r120	0	1	1	1	1	0	0	0	none
chr3:63825167..63825167+	chr3 +	63825167	63825167	0,241514452	60511	NM_001104591	Vmn2r120	0	1	1	1	1	0	0	0	none
chr3:63825206..63825221+	chr3 +	63825206	63825221	0,458877459	60396	NM_001104591	Vmn2r120	0	1	1	1	1	1	0	0	none
chr3:63825258..63825258+	chr3 +	63825258	63825258	0,024151445	60342	NM_001104591	Vmn2r120	0	1	1	1	1	1	0	0	none
chr3:63825289..63825312+	chr3 +	63825289	63825312	0,289817343	60305	NM_001104591	Vmn2r120	0	1	1	1	1	1	1	0	none
chr3:63825395..63825397+	chr3 +	63825395	63825397	0,04830289	60251	NM_001104591	Vmn2r120	0	1	1	1	1	1	1	0	none
chr3:63826181..63826181+	chr3 +	63826181	63826181	0,024151445	60168	NM_001104591	Vmn2r120	0	1	1	1	1	1	1	0	none
chr3:63826792..63826792+	chr3 +	63826792	63826792	0,024151445	59382	NM_001104591	Vmn2r120	0	1	1	1	1	0	0	0	none
chr3:63826931..63826931+	chr3 +	63826931	63826931	0,120757226	58771	NM_001104591	Vmn2r120	0	1	0	0	0	0	0	0	none
chr3:63827066..63827066+	chr3 +	63827066	63827066	0,144908671	58632	NM_001104591	Vmn2r120	0	1	0	0	0	0	0	0	none
chr3:63827307..63827307+	chr3 +	63827307	63827307	0,144908671	58497	NM_001104591	Vmn2r120	0	1	0	0	0	0	0	0	none
chr3:63836620..63836620+	chr3 +	63836620	63836620	0,096605781	58256	NM_001104591	Vmn2r120	0	1	0	0	0	0	0	0	none
chr3:63844392..63844393+	chr3 +	63844392	63844393	0,072454336	48943	NM_001104591	Vmn2r120	0	0	0	0	0	0	0	0	none
chr3:63844795..63844795+	chr3 +	63844795	63844795	0,120757226	41171	NM_001104591	Vmn2r120	0	0	0	0	0	0	0	0	none
chr3:63850461..63850461+	chr3 +	63850461	63850461	0,024151445	40768	NM_001104591	Vmn2r120	0	0	0	0	0	0	0	0	none
chr3:63857759..63857759+	chr3 +	63857759	63857759	0,024151445	35102	NM_001104591	Vmn2r120	1	0	0	0	0	0	0	0	LTR
chr3:63862779..63862779+	chr3 +	63862779	63862779	0,02415												

chr3:63882119..63882119+	chr3	+	63882119	63882119	0,024151445	3444	NM_019918	Vmn2r1	1	1	1	1	1	0	0	1	SINE
chr3:63883053..63883053+	chr3	+	63883053	63883053	0,241514452	2510	NM_019918	Vmn2r1	0	0	1	1	1	0	0	1	none
chr3:63946788..63946788-	chr3	-	63946788	63946788	0,169060117	5394	NM_001104592	Vmn2r2	0	1	1	1	1	0	0	1	none
chr3:63947613..63947613-	chr3	-	63947613	63947613	0,024151445	6219	NM_001104592	Vmn2r2	0	1	1	1	1	0	0	1	none
chr3:63958175..63958175-	chr3	-	63958175	63958175	0,120757226	16781	NM_001104592	Vmn2r2	0	0	0	0	0	0	0	0	none
chr3:63986988..63986992-	chr3	-	63986988	63986992	0,072454336	45594	NM_001104592	Vmn2r2	1	0	0	0	0	0	0	0	LTR
chr3:63987380..63987380-	chr3	-	63987380	63987380	0,024151445	45986	NM_001104592	Vmn2r2	1	0	0	0	0	0	0	0	LTR
chr3:63999327..63999327-	chr3	-	63999327	63999327	0,144908671	57933	NM_001104592	Vmn2r2	1	1	0	0	0	0	0	0	none
chr3:64003359..64003359-	chr3	-	64003359	64003359	0,024151445	61965	NM_001104592	Vmn2r2	0	1	0	0	0	0	0	0	none
chr3:64019597..64019597-	chr3	-	64019597	64019597	0,024151445	78203	NM_001104592	Vmn2r2	1	1	0	0	0	0	0	0	LINE
chr3:64029680..64029680-	chr3	-	64029680	64029680	0,072454336	88286	NM_001104592	Vmn2r2	0	1	0	0	0	0	0	0	none
chr3:64057783..64057783-	chr3	-	64057783	64057783	0,024151445	116389	NM_001104592	Vmn2r2	1	0	0	0	0	0	0	0	LTR
chr3:64060157..64060157-	chr3	-	64060157	64060157	0,024151445	118763	NM_001104592	Vmn2r2	1	0	0	0	0	0	0	0	LTR
chr3:64091506..64091519-	chr3	-	64091506	64091519	0,241514452	174	NM_001104614	Vmn2r3	0	1	1	1	1	0	1	1	none
chr3:64094414..64094414-	chr3	-	64094414	64094414	0,024151445	3080	NM_001104614	Vmn2r3	0	1	1	1	1	0	0	1	none
chr3:64096467..64096467-	chr3	-	64096467	64096467	0,024151445	5133	NM_001104614	Vmn2r3	1	1	0	0	0	0	0	1	LINE
chr3:64103499..64103499-	chr3	-	64103499	64103499	0,024151445	12165	NM_001104614	Vmn2r3	1	0	0	0	0	0	0	0	Low_complexity
chr3:64126223..64126225-	chr3	-	64126223	64126225	1,135117926	34891	NM_001104614	Vmn2r3	1	0	0	0	0	0	0	0	LTR
chr3:64127535..64127535-	chr3	-	64127535	64127535	0,04830289	36201	NM_001104614	Vmn2r3	1	0	0	0	0	0	1	0	SINE
chr3:64133184..64133184-	chr3	-	64133184	64133184	0,289817343	41850	NM_001104614	Vmn2r3	1	0	0	0	0	1	0	0	LINE
chr3:64184445..64184445-	chr3	-	64184445	64184445	0,024151445	93111	NM_001104614	Vmn2r3	0	0	0	0	0	0	1	0	none
chr3:64244300..64244300-	chr3	-	64244300	64244300	0,024151445	30329	NM_001104615	Vmn2r4	0	1	1	0	1	0	0	0	none
chr3:64264303..64264303-	chr3	-	64264303	64264303	0,217363007	50332	NM_001104615	Vmn2r4	0	0	1	0	1	0	0	0	none
chr3:64266219..64266219-	chr3	-	64266219	64266219	0,024151445	52248	NM_001104615	Vmn2r4	0	0	1	0	1	0	0	0	none
chr3:64364420..64364420-	chr3	-	64364420	64364420	0,096605781	688	NM_001104619	Vmn2r6	1	1	1	0	0	0	0	1	LINE
chr3:64364627..64364627-	chr3	-	64364627	64364627	0,024151445	895	NM_001104619	Vmn2r6	1	1	1	0	0	0	0	1	LINE
chr3:64365307..64365307-	chr3	-	64365307	64365307	0,120757226	1575	NM_001104619	Vmn2r6	0	1	1	1	1	0	0	1	none
chr3:64372552..64372554-	chr3	-	64372552	64372554	0,04830289	8820	NM_001104619	Vmn2r6	0	0	1	1	1	1	0	0	none
chr3:64390979..64390979-	chr3	-	64390979	64390979	0,096605781	27247	NM_001104619	Vmn2r6	1	0	0	0	0	0	0	0	LTR
chr3:64391517..64391517-	chr3	-	64391517	64391517	0,024151445	27785	NM_001104619	Vmn2r6	1	0	0	0	0	0	0	0	LTR
chr3:64402119..64402122-	chr3	-	64402119	64402122	0,724543357	38390	NM_001104619	Vmn2r6	1	0	1	0	0	0	0	0	DNA
chr3:64469313..64469313-	chr3	-	64469313	64469313	0,120757226	105581	NM_001104619	Vmn2r6	1	1	1	0	1	0	0	0	LTR
chr3:64487939..64487939-	chr3	-	64487939	64487939	0,024151445	124207	NM_001104619	Vmn2r6	0	0	0	0	0	0	0	0	none
chr3:64538533..64538533-	chr3	-	64538533	64538533	0,217363007	15017	NM_175674	Vmn2r7	0	1	1	0	0	0	0	0	none
chr3:64555384..64555384-	chr3	-	64555384	64555384	0,024151445	31868	NM_175674	Vmn2r7	1	0	0	0	0	0	0	0	LINE
chr3:64555867..64555867-	chr3	-	64555867	64555867	0,024151445	32351	NM_175674	Vmn2r7	1	0	0	0	0	0	0	0	LINE
chr3:64581902..64581902-	chr3	-	64581902	64581902	0,265665897	58386	NM_175674	Vmn2r7	0	1	1	0	0	0	0	0	none
chr3:64582363..64582363-	chr3	-	64582363	64582363	0,120757226	58847	NM_175674	Vmn2r7	0	1	0	0	0	0	0	0	none
chr3:64583136..64583136-	chr3	-	64583136	64583136	0,04830289	59620	NM_175674	Vmn2r7	0	1	0	0	0	0	0	0	none
chr3:64589993..64589993-	chr3	-	64589993	64589993	0,144908671	66477	NM_175674	Vmn2r7	1	0	0	0	0	0	0	0	LINE
chr3:64591824..64591824-	chr3	-	64591824	64591824	0,024151445	68308	NM_175674	Vmn2r7	1	0	0	0	0	0	0	0	LINE
chr3:64624477..64624477-	chr3	-	64624477	64624477	0,024151445	100961	NM_175674	Vmn2r7	1	0	0	0	0	0	0	0	LINE
chr3:64629933..64629933-	chr3	-	64629933	64629933	0,386423124	106417	NM_175674	Vmn2r7	0	0	0	0	0	0	0	0	none
chr3:64644641..64644641-	chr3	-	64644641	64644641	0,024151445	121125	NM_175674	Vmn2r7	1	0	0	0	0	0	0	0	LINE
chr3:64647840..64647840-	chr3	-	64647840	64647840	0,04830289	124324	NM_175674	Vmn2r7	1	0	0	0	0	0	0	0	LTR
chr3:64650655..64650655-	chr3	-	64650655	64650655	0,072454336	127139	NM_175674	Vmn2r7	0	0	0	0	0	0	0	0	none
chr3:64651122..64651122-	chr3	-	64651122	64651122	0,362271678	127606	NM_175674	Vmn2r7	1	0	0	0	0	0	0	0	LINE
chr3:64667984..64667984-	chr3	-	64667984	64667984	0,024151445	144468	NM_175674	Vmn2r7	0	0	0	0	0	0	0	0	none
chr3:64670821..64670821-	chr3	-	64670821	64670821	0,072454336	147305	NM_175674	Vmn2r7	1	0	0	0	0	0	0	0	Simple_repeat
chr3:64672196..64672196-	chr3	-	64672196	64672196	0,169060117	148680	NM_175674	Vmn2r7	0	0	0	0	0	0	0	0	none
chr3:64683766..64683766-	chr3	-	64683766	64683766	0,024151445	160250	NM_175674	Vmn2r7	0	1	0	0	0	0	0	0	none
chr3:64695607..64695607-	chr3	-	64695607	64695607	0,024151445	172091	NM_175674	Vmn2r7	1	1	0	0	0	0	0	0	LINE
chr3:64703782..64703782-	chr3	-	64703782	64703782	0,120757226	180266	NM_175674	Vmn2r7	0	1	1	0	0	1	1	0	none
chr3:64710855..64710855-	chr3	-	64710855	64710855	0,193211562	187339	NM_175674	Vmn2r7	0	0	0	0	0	0	0	0	none
chr3:64714927..64714927-	chr3	-	64714927	64714927	0,096605781	191411	NM_175674	Vmn2r7	0	1	0	0	0	0	0	0	none
chr3:64750895..64750897-	chr3	-	64750895	64750897	0,241514452	227381	NM_175674	Vmn2r7	1	1	1	0	0	0	0	0	LTR
chr3:64751857..64751857-	chr3	-	64751857	64751857	0,241514452	228341	NM_175674	Vmn2r7	0	1	1	0	0	1	0	0	none
chr3:64751998..64751998-	chr3	-	64751998	64751998	0,024151445	228482	NM_175674	Vmn2r7	0	1	1	0	1	0	0	0	none
chr3:64753120..64753120-	chr3	-	64753120	64753120	0,289817343	229604	NM_175674	Vmn2r7	0	1	1	0	1	1	1	0	none
chr3:64754896..64754896-	chr3	-	64754896	64754896	0,024151445	231380	NM_175674	Vmn2r7	1	1	1	0	1	0	0	0	SINE
chr3:64755959..64755959-	chr3	-	64755959	64755959	0,096605781	232443	NM_175674	Vmn2r7	0	1	1	0	1	0	0	0	none
chr3:64756064..64756064-	chr3	-	64756064	64756064	0,120757226	232548	NM_175674	Vmn2r7	0	1	1	0	1	0	0	0	none
chr3:64766966..64766966-	chr3	-	64766966	64766966	0,024151445	243450	NM_175674	Vmn2r7	1	1	0	0	0	0	0	0	LINE
chr3:64770266..64770269-	chr3	-	64770266	64770269	0,169060117	246753	NM_175674	Vmn2r7	0	1	1	0	1	0	0	0	none
chr3:64794464..64794464-	chr3	-	64794464	64794464	0,072454336	270948	NM_175674	Vmn2r7	1	1	1	1	1	0	0	0	LINE
chr3:64802425..64802425-	chr3	-	64802425	64802425	0,024151445	278909	NM_175674	Vmn2r7	0	1	1	1	1	0	0	0	none
chr3:64806949..64806949-	chr3	-	64806949	64806949	0,072454336	283433	NM_175674	Vmn2r7	0	1	1	1	1	0	0	0	none
chr3:64812938..64812938-	chr3	-	64812938	64812938	0,024151445	289422	NM_175674	Vmn2r7	1	1	1	1	1	0	0	0	LINE
chr3:64813204..64813204-	chr3	-	64813204	64813204	0,072454336	289688	NM_175674	Vmn2r7	0	0	1	1	1	0	0	0	none
chr3:64825546..64825546-	chr3	-	64825546	64825546	0,04830289	302030	NM_175674	Vmn2r7	0	1	1	0	1	0	0	0	none
chr3:64839851..64839851-	chr3	-	64839851	64839851	0,024151445	316335	NM_175674	Vmn2r7	0	1	1	1	1	0	0	0	none
chr3:64846693..64846693-	chr3	-	64846693	64846693	0,024151445	323177	NM_175674	Vmn2r7	0	1	1	1	1	0	0	0	none

chr5:109251379..109251379-	chr5	-	109251379	109251379	0,144908671	13105	NM_001104620	Vmn2r8	0	0	0	0	0	1	1	0	none
chr5:109257492..109257492-	chr5	-	109257492	109257492	0,024151445	19218	NM_001104620	Vmn2r8	1	0	0	0	0	0	0	0	LINE
chr5:109284123..109284123-	chr5	-	109284123	109284123	0,120757226	2093	NM_001104621	Vmn2r9	0	1	0	0	0	0	0	1	none
chr5:109286287..109286287-	chr5	-	109286287	109286287	0,024151445	4257	NM_001104621	Vmn2r9	1	0	0	0	0	0	0	0	LINE
chr5:109340508..109340508-	chr5	-	109340508	109340508	0,024151445	58478	NM_001104621	Vmn2r9	1	0	0	0	0	0	0	0	LINE
chr5:109341829..109341829-	chr5	-	109341829	109341829	0,04830289	59799	NM_001104621	Vmn2r9	1	0	0	0	0	1	1	0	LTR
chr5:109348132..109348132-	chr5	-	109348132	109348132	0,144908671	66102	NM_001104621	Vmn2r9	1	0	0	0	0	0	0	0	LINE
chr5:109350466..109350466-	chr5	-	109350466	109350466	0,024151445	68436	NM_001104621	Vmn2r9	1	0	0	0	0	0	0	0	LTR
chr5:109360180..109360180-	chr5	-	109360180	109360180	0,120757226	78150	NM_001104621	Vmn2r9	1	0	0	0	0	0	0	0	LINE
chr5:109372080..109372080-	chr5	-	109372080	109372080	0,024151445	90050	NM_001104621	Vmn2r9	1	1	0	0	0	0	0	0	LTR
chr5:109444585..109444585-	chr5	-	109444585	109444585	0,083432265	8629	NM_009491	Vmn2r10	0	0	0	0	0	1	1	0	none
chr5:109444680..109444680-	chr5	-	109444680	109444680	0,024151445	8724	NM_009491	Vmn2r10	0	0	0	0	0	1	1	0	none
chr5:109444738..109444738-	chr5	-	109444738	109444738	0,001463724	8782	NM_009491	Vmn2r10	0	0	0	0	0	1	1	0	none
chr5:109569195..109569195-	chr5	-	109569195	109569195	0,024151445	41811	NM_001104623	Vmn2r12	1	0	0	0	0	0	0	0	LINE
chr5:109582067..109582067-	chr5	-	109582067	109582067	0,193211562	54683	NM_001104623	Vmn2r12	0	0	0	0	0	0	0	0	none
chr5:109633965..109633965-	chr5	-	109633965	109633965	0,024151445	12338	NM_001104624	Vmn2r13	1	0	0	0	0	0	0	0	LTR
chr5:109636454..109636454-	chr5	-	109636454	109636454	0,096005781	14827	NM_001104624	Vmn2r13	1	0	0	0	0	0	0	0	LINE
chr5:109636546..109636546-	chr5	-	109636546	109636546	0,04830289	14919	NM_001104624	Vmn2r13	1	0	0	0	0	0	0	0	LINE
chr5:109655591..109655591-	chr5	-	109655591	109655591	0,072454336	1449	NM_001104625	Vmn2r14	1	0	0	0	0	0	0	0	LINE
chr5:109669659..109669660-	chr5	-	109669659	109669660	0,289817343	15518	NM_001104625	Vmn2r14	1	0	0	0	0	0	0	0	LTR
chr5:109681227..109681227-	chr5	-	109681227	109681227	0,04830289	27085	NM_001104625	Vmn2r14	1	0	0	0	0	0	0	0	LINE
chr5:109681651..109681651-	chr5	-	109681651	109681651	0,096605781	27509	NM_001104625	Vmn2r14	1	0	0	0	0	0	0	0	LINE
chr5:109735440..109735440-	chr5	-	109735440	109735440	0,024151445	8364	NM_001104626	Vmn2r15	1	0	0	0	0	0	0	0	LINE
chr5:109740681..109740681-	chr5	-	109740681	109740681	0,024151445	13605	NM_001104626	Vmn2r15	1	0	0	0	0	0	0	0	LTR
chr5:109742028..109742028+	chr5	+	109742028	109742028	0,024151445	17371	NM_001104627	Vmn2r16	1	0	0	0	0	0	0	0	LINE
chr5:109742100..109742100+	chr5	+	109742100	109742100	0,024151445	17299	NM_001104627	Vmn2r16	1	0	0	0	0	0	0	0	LINE
chr5:109752792..109752796-	chr5	-	109752792	109752796	0,748694802	25720	NM_001104626	Vmn2r15	1	0	0	0	0	0	0	0	LINE
chr5:109755952..109755952-	chr5	-	109755952	109755952	0,024151445	28876	NM_001104626	Vmn2r15	1	0	0	0	0	0	0	0	LINE
chr5:109758683..109758683-	chr5	-	109758683	109758683	0,120757226	31607	NM_001104626	Vmn2r15	0	0	0	0	0	0	0	0	none
chr5:109804396..109804396+	chr5	+	109804396	109804396	0,072454336	44635	NM_001104628	Vmn2r17	1	0	0	0	0	0	0	0	LINE
chr5:109826346..109826346+	chr5	+	109826346	109826346	0,144908671	22685	NM_001104628	Vmn2r17	1	0	0	0	0	1	0	0	LINE
chr5:109832410..109832410+	chr5	+	109832410	109832410	0,024151445	16621	NM_001104628	Vmn2r17	1	0	0	0	0	1	0	0	LINE
chr5:152402935..152402935-	chr5	-	152402935	152402935	0,024151445	13453	NM_001102582	Vmn2r18	0	0	0	0	0	0	0	0	none
chr5:152417552..152417553-	chr5	-	152417552	152417553	0,144908671	28071	NM_001102582	Vmn2r18	1	0	0	0	0	0	0	0	LINE
chr5:152423217..152423217-	chr5	-	152423217	152423217	0,120757226	33735	NM_001102582	Vmn2r18	1	0	0	0	0	0	0	0	LINE
chr5:152436153..152436153-	chr5	-	152436153	152436153	0,024151445	46671	NM_001102582	Vmn2r18	1	0	0	0	0	0	0	0	LINE
chr5:152441379..152441379-	chr5	-	152441379	152441379	0,144908671	51897	NM_001102582	Vmn2r18	1	0	0	0	0	0	0	0	LINE
chr5:152443353..152443353-	chr5	-	152443353	152443353	0,024151445	53871	NM_001102582	Vmn2r18	1	1	0	0	0	0	0	0	LTR
chr6:123256503..123256503+	chr6	+	123256503	123256503	0,144908671	1847	NM_001104632	Vmn2r19	1	0	0	0	0	0	0	1	LTR
chr6:123258176..123258176+	chr6	+	123258176	123258176	0,169060117	174	NM_001104632	Vmn2r19	0	1	0	1	0	0	1	1	none
chr6:123606607..123606607-	chr6	-	123606607	123606607	0,024151445	5453	NM_001104637	Vmn2r22	1	0	0	0	0	0	0	0	LTR
chr6:123611926..123611926-	chr6	-	123611926	123611926	0,024151445	10772	NM_001104637	Vmn2r22	1	1	0	1	0	1	0	0	SINE
chr6:123613896..123613896-	chr6	-	123613896	123613896	0,096605781	12742	NM_001104637	Vmn2r22	1	0	0	0	0	0	0	0	LINE
chr6:123617815..123617815+	chr6	+	123617815	123617815	0,024151445	35023	NM_001104638	Vmn2r23	1	0	0	0	0	0	0	0	LINE
chr6:123618766..123618766+	chr6	+	123618766	123618766	0,072454336	34072	NM_001104638	Vmn2r23	0	1	0	0	0	0	0	0	none
chr6:123631973..123631973-	chr6	-	123631973	123631973	0,024151445	30819	NM_001104637	Vmn2r22	1	0	0	0	0	0	0	0	LTR
chr6:123635988..123635988-	chr6	-	123635988	123635988	0,024151445	34834	NM_001104637	Vmn2r22	1	0	0	0	0	0	0	0	LINE
chr6:123643887..123643888+	chr6	+	123643887	123643888	0,009056792	8950	NM_001104638	Vmn2r23	0	0	0	0	0	0	0	0	none
chr6:123643937..123643947+	chr6	+	123643937	123643947	0,072454336	8891	NM_001104638	Vmn2r23	0	0	0	0	0	0	0	0	none
chr6:123643973..123643983+	chr6	+	123643973	123643983	0,169060117	8855	NM_001104638	Vmn2r23	0	0	0	0	0	0	0	0	none
chr6:123648352..123648352+	chr6	+	123648352	123648352	0,024151445	4486	NM_001104638	Vmn2r23	1	0	0	0	0	0	0	0	LTR
chr6:123648994..123648994+	chr6	+	123648994	123648994	0,024151445	3844	NM_001104638	Vmn2r23	1	0	0	0	0	0	0	0	LTR
chr6:123651683..123651683+	chr6	+	123651683	123651683	0,024151445	1155	NM_001104638	Vmn2r23	1	0	0	0	0	0	0	0	Simple_repeat
chr6:123652651..123652668+	chr6	+	123652651	123652668	0,053669878	170	NM_001104638	Vmn2r23	0	1	0	1	0	1	1	1	none
chr6:123704676..123704676+	chr6	+	123704676	123704676	0,024151445	24312	NM_001104639	Vmn2r24	1	0	0	0	0	0	0	0	LTR
chr6:123727842..123727842+	chr6	+	123727842	123727842	0,024151445	1146	NM_001104639	Vmn2r24	0	0	0	0	0	0	0	0	none
chr6:123804752..123804755+	chr6	+	123804752	123804755	0,072454336	170023	NM_019917	Vmn2r26	0	1	0	0	0	0	0	0	none
chr6:123809044..123809044+	chr6	+	123809044	123809044	0,024151445	165731	NM_019917	Vmn2r26	1	0	0	0	0	0	0	0	LINE
chr6:123820971..123820971+	chr6	+	123820971	123820971	0,024151445	153804	NM_019917	Vmn2r26	1	0	0	0	0	0	0	0	LINE
chr6:123827063..123827063+	chr6	+	123827063	123827063	0,120757226	147712	NM_019917	Vmn2r26	1	0	0	0	0	0	0	0	LINE
chr6:123834078..123834078+	chr6	+	123834078	123834078	0,024151445	140697	NM_019917	Vmn2r26	1	0	0	0	0	0	0	0	LINE
chr6:123836586..123836586+	chr6	+	123836586	123836586	0,024151445	138189	NM_019917	Vmn2r26	0	0	0	0	0	1	1	0	none
chr6:123842681..123842681-	chr6	-	123842681	123842681	0,024151445	38972	NM_001104641	Vmn2r25	0	0	0	0	0	0	0	0	none
chr6:123844266..123844267-	chr6	-	123844266	123844267	0,144908671	40558	NM_001104641	Vmn2r25	1	0	0	0	0	0	0	0	LINE
chr6:123846004..123846004+	chr6	+	123846004	123846004	0,144908671	128771	NM_019917	Vmn2r26	0	1	0	0	0	0	0	0	none
chr6:123856303..123856303-	chr6	-	123856303	123856303	0,120757226	52594	NM_001104641	Vmn2r25	1	0	0	0	0	0	0	0	LINE
chr6:123859016..123859016+	chr6	+	123859016	123859016	0,024151445	115759	NM_019917	Vmn2r26	1	0	0	0	0	0	0	0	LINE
chr6:123859110..123859110+	chr6	+	123859110	123859110	0,024151445	115665	NM_019917	Vmn2r26	1	0	0	0	0	0	0	0	LINE
chr6:123864790..123864790+	chr6	+	123864790	123864790	0,024151445	109985	NM_019917	Vmn2r26	0	0	0	0	0	0	0	0	none
chr6:123877490..123877490-	chr6	-	123877490	123877490	0,024151445	73781	NM_001104641	Vmn2r25	1	0							

chr6:123897796..123897796+chr6	+	123897796	123897796	0,024151445	76979	NM_019917	Vmn2r26	1	0	0	0	0	0	0	0	0	SINE
chr6:123903719..123903719+chr6	+	123903719	123903719	0,024151445	71056	NM_019917	Vmn2r26	1	1	0	0	1	0	0	0	0	SINE
chr6:123903735..123903735+chr6	+	123903735	123903735	0,144908671	100026	NM_001104641	Vmn2r25	1	1	0	0	1	0	0	0	0	SINE
chr6:123904923..123904923+chr6	+	123904923	123904923	0,144908671	69852	NM_019917	Vmn2r26	0	1	0	0	0	0	0	0	0	none
chr6:123906846..123906846+chr6	+	123906846	123906846	0,169060117	103137	NM_001104641	Vmn2r25	0	1	0	0	0	0	0	0	0	none
chr6:123911561..123911562+chr6	+	123911561	123911562	1,0143607	63214	NM_019917	Vmn2r26	0	1	0	0	0	0	0	0	0	none
chr6:123911617..123911617+chr6	+	123911617	123911617	0,024151445	107908	NM_001104641	Vmn2r25	0	1	0	0	0	0	0	0	0	none
chr6:123913584..123913584+chr6	+	123913584	123913584	0,024151445	109875	NM_001104641	Vmn2r25	1	0	0	0	0	0	0	0	0	LINE
chr6:123913840..123913840+chr6	+	123913840	123913840	0,036227168	110131	NM_001104641	Vmn2r25	1	0	0	0	0	0	0	0	0	LINE
chr6:123916191..123916191+chr6	+	123916191	123916191	0,024151445	58584	NM_019917	Vmn2r26	1	0	0	0	0	0	0	0	0	LINE
chr6:123919645..123919645+chr6	+	123919645	123919645	0,024151445	115936	NM_001104641	Vmn2r25	0	1	0	0	1	0	1	1	0	none
chr6:123922722..123922722+chr6	+	123922722	123922722	0,04830289	52053	NM_019917	Vmn2r26	0	1	0	0	0	0	0	0	0	none
chr6:123928160..123928164+chr6	+	123928160	123928164	1,183420816	124454	NM_001104641	Vmn2r25	0	1	0	1	0	0	1	0	0	none
chr6:123929472..123929472+chr6	+	123929472	123929472	0,096605781	125763	NM_001104641	Vmn2r25	1	0	0	0	0	0	0	0	0	LINE
chr6:123942973..123942973+chr6	+	123942973	123942973	0,169060117	31802	NM_019917	Vmn2r26	1	0	0	0	0	0	0	0	0	LINE
chr6:123943833..123943833+chr6	+	123943833	123943833	0,096605781	140124	NM_001104641	Vmn2r25	0	1	0	0	0	0	0	0	0	none
chr6:123955013..123955013+chr6	+	123955013	123955013	0,04830289	151304	NM_001104641	Vmn2r25	1	0	0	0	0	0	0	0	0	LINE
chr6:123956134..123956134+chr6	+	123956134	123956134	0,096605781	18641	NM_019917	Vmn2r26	1	0	0	0	0	0	0	0	0	LINE
chr6:123973514..123973514+chr6	+	123973514	123973514	0,04830289	1261	NM_019917	Vmn2r26	1	1	0	0	0	0	0	0	1	SINE
chr6:123974586..123974589+chr6	+	123974586	123974589	0,067087348	189	NM_019917	Vmn2r26	0	1	0	1	0	0	1	1	0	none
chr6:123974671..123974671+chr6	+	123974671	123974671	0,120757226	104	NM_019917	Vmn2r26	0	1	0	1	0	0	1	1	0	none
chr6:124199132..124199132+chr6	+	124199132	124199132	0,04830289	16829	NM_001104642	Vmn2r27	1	0	0	0	0	0	0	0	0	LINE
chr6:124205850..124205850+chr6	+	124205850	124205850	0,024151445	23547	NM_001104642	Vmn2r27	0	1	0	1	1	0	0	0	0	none
chr6:124229204..124229204+chr6	+	124229204	124229204	0,072454336	46901	NM_001104642	Vmn2r27	0	1	0	1	1	0	0	0	0	none
chr6:124231846..124231846+chr6	+	124231846	124231846	0,04830289	49543	NM_001104642	Vmn2r27	0	1	0	0	0	0	0	0	0	none
chr6:124250854..124250854+chr6	+	124250854	124250854	0,024151445	68551	NM_001104642	Vmn2r27	0	1	0	0	0	0	0	0	0	none
chr6:124251365..124251365+chr6	+	124251365	124251365	0,120757226	69062	NM_001104642	Vmn2r27	1	1	0	0	0	0	0	0	0	LINE
chr7:10446353..10446353+chr7	+	10446353	10446353	0,024151445	19178	NM_001105151	Vmn2r47	1	0	0	0	0	0	0	0	0	LTR
chr7:10583545..10583545+chr7	+	10583545	10583545	0,024151445	5556	NM_001105156	Vmn2r49	1	0	0	0	0	0	0	0	0	LINE
chr7:10621647..10621647+chr7	+	10621647	10621647	0,024151445	43658	NM_001105156	Vmn2r49	1	1	0	0	0	0	0	0	0	LINE
chr7:10692481..10692481+chr7	+	10692481	10692481	0,04830289	972	NM_001105179	Vmn2r51	1	0	0	0	0	0	0	0	0	SINE
chr7:10692563..10692563+chr7	+	10692563	10692563	0,024151445	1054	NM_001105179	Vmn2r51	0	0	0	0	0	0	0	0	0	none
chr7:10699621..10699621+chr7	+	10699621	10699621	0,313968788	8112	NM_001105179	Vmn2r51	1	0	0	0	0	0	0	0	0	LINE
chr7:10699652..10699652+chr7	+	10699652	10699652	0,024151445	8143	NM_001105179	Vmn2r51	1	0	0	0	0	0	0	0	0	LINE
chr7:10725491..10725491+chr7	+	10725491	10725491	0,04830289	33982	NM_001105179	Vmn2r51	1	0	0	0	0	0	0	0	0	LTR
chr7:10725995..10725995+chr7	+	10725995	10725995	0,024151445	34486	NM_001105179	Vmn2r51	0	0	0	0	0	0	0	0	0	none
chr7:10732261..10732261+chr7	+	10732261	10732261	0,096605781	40752	NM_001105179	Vmn2r51	1	0	0	0	0	0	0	0	0	LINE
chr7:10740998..10740998+chr7	+	10740998	10740998	0,096605781	49489	NM_001105179	Vmn2r51	1	0	0	0	0	0	0	0	0	LINE
chr7:10770299..10770299+chr7	+	10770299	10770299	0,169060117	8163	NM_001105191	Vmn2r52	0	0	0	0	0	0	0	0	0	none
chr7:10788008..10788008+chr7	+	10788008	10788008	0,024151445	25872	NM_001105191	Vmn2r52	0	0	0	0	0	0	1	0	0	none
chr7:10820837..10820837+chr7	+	10820837	10820837	0,024151445	58701	NM_001105191	Vmn2r52	0	0	0	0	0	0	0	0	0	none
chr7:10823451..10823451+chr7	+	10823451	10823451	0,024151445	61315	NM_001105191	Vmn2r52	0	0	0	0	0	0	0	0	0	none
chr7:10824412..10824412+chr7	+	10824412	10824412	0,096605781	62276	NM_001105191	Vmn2r52	1	0	0	0	0	0	0	0	0	LINE
chr7:10828745..10828745+chr7	+	10828745	10828745	0,04830289	66609	NM_001105191	Vmn2r52	1	0	0	0	0	0	0	0	0	LINE
chr7:10832029..10832029+chr7	+	10832029	10832029	0,04830289	69893	NM_001105191	Vmn2r52	1	0	0	0	0	0	0	0	0	LINE
chr7:10836606..10836606+chr7	+	10836606	10836606	0,024151445	74470	NM_001105191	Vmn2r52	0	0	0	0	0	0	0	0	0	LTR
chr7:10841411..10841411+chr7	+	10841411	10841411	0,024151445	79275	NM_001105191	Vmn2r52	0	0	0	0	0	0	0	0	0	none
chr7:10841521..10841521+chr7	+	10841521	10841522	0,096605781	79386	NM_001105191	Vmn2r52	1	0	0	0	0	0	0	0	0	SINE
chr7:10845126..10845126+chr7	+	10845126	10845126	0,072454336	82990	NM_001105191	Vmn2r52	1	0	0	0	0	0	0	0	0	LINE
chr7:10855566..10855566+chr7	+	10855566	10855566	0,024151445	93430	NM_001105191	Vmn2r52	1	0	0	0	0	0	0	0	0	LINE
chr7:13192880..13192880+chr7	+	13192880	13192880	0,024151445	486	NM_001104644	Vmn2r53	0	1	1	0	0	0	0	0	1	none
chr7:13325442..13325442+chr7	+	13325442	13325442	0,193211562	6487	NM_001104648	Vmn2r56	1	0	0	0	0	0	0	0	1	LTR
chr7:13325763..13325763+chr7	+	13325763	13325763	0,217363007	6808	NM_001104648	Vmn2r56	0	0	0	0	0	0	0	0	1	none
chr7:13327915..13327915+chr7	+	13327915	13327915	0,024151445	8960	NM_001104648	Vmn2r56	0	0	0	0	0	1	0	1	0	none
chr7:13353517..13353517+chr7	+	13353517	13353517	0,072454336	34562	NM_001104648	Vmn2r56	0	1	1	1	1	1	1	0	0	none
chr7:13353655..13353655+chr7	+	13353655	13353655	0,144908671	34700	NM_001104648	Vmn2r56	0	1	1	1	1	1	1	0	0	none
chr7:13360053..13360053+chr7	+	13360053	13360053	0,024151445	41098	NM_001104648	Vmn2r56	0	1	1	1	1	1	1	1	0	none
chr7:13360179..13360179+chr7	+	13360179	13360179	0,04830289	41224	NM_001104648	Vmn2r56	0	1	1	1	1	1	1	1	0	none
chr7:13360803..13360804+chr7	+	13360803	13360804	0,169060117	41849	NM_001104648	Vmn2r56	0	1	1	1	1	0	1	0	0	none
chr7:13363084..13363084+chr7	+	13363084	13363084	0,024151445	44129	NM_001104648	Vmn2r56	0	1	1	1	1	0	0	0	0	none
chr7:13365965..13366001+chr7	+	13365965	13366001	1,980418509	47010	NM_001104648	Vmn2r56	0	1	1	1	1	1	0	0	0	none
chr7:13366023..13366023+chr7	+	13366023	13366023	0,096605781	47068	NM_001104648	Vmn2r56	0	1	1	1	1	0	0	0	0	none
chr7:13375346..13375346+chr7	+	13375346	13375346	0,024151445	56391	NM_001104648	Vmn2r56	0	1	0	0	0	0	1	0	0	none
chr7:13378089..13378089+chr7	+	13378089	13378089	0,386423124	59134	NM_001104648	Vmn2r56	0	0	0	0	0	0	0	0	0	none
chr7:13378174..13378174+chr7	+	13378174	13378174	0,072454336	59219	NM_001104648	Vmn2r56	0	0	0	0	0	0	0	0	0	none
chr7:13378212..13378220+chr7	+	13378212	13378220	0,265665897	59257	NM_001104648	Vmn2r56	0	0	0	0	0	0	0	0	0	none
chr7:13379187..13379187+chr7	+	13379187	13379187	0,362271678	60232	NM_001104648	Vmn2r56	0	0	0	0	0	0	0	0	0	none
chr7:13379222..13379224+chr7	+	13379222	13379224	0,169060117	60269	NM_001104648	Vmn2r56	0	0	0	0	0	0	0	0	0	none
chr7:13381138..13381138+chr7	+	13381138	13381138	0,024151445	62183	NM_001104648	Vmn2r56	0	1	0	0	0	0	1	0	0	none
chr7:13381611..13381611+chr7	+	13381611	13381611	0,410574569	62656	NM_001104648	Vmn2r56	0	1	0	1	0	1	0			

chr7:13384646..13384683-	chr7	-	13384646	13384683	0,627937576	65728	NM_001104648	Vmn2r56	1	1	1	1	1	1	1	0	Simple_repeat
chr7:13384721..13384721-	chr7	-	13384721	13384721	0,024151445	65766	NM_001104648	Vmn2r56	0	1	1	1	1	1	1	0	none
chr7:13384917..13384917-	chr7	-	13384917	13384917	0,024151445	65962	NM_001104648	Vmn2r56	0	1	1	1	1	1	1	0	none
chr7:13385085..13385092-	chr7	-	13385085	13385092	0,131257854	66137	NM_001104648	Vmn2r56	0	1	1	0	1	1	1	0	none
chr7:13385180..13385180-	chr7	-	13385180	13385180	0,04830289	66225	NM_001104648	Vmn2r56	0	1	1	0	1	1	1	0	none
chr7:13385267..13385267-	chr7	-	13385267	13385267	0,024151445	66312	NM_001104648	Vmn2r56	0	1	1	0	1	1	1	0	none
chr7:13385288..13385288-	chr7	-	13385288	13385288	0,096605781	66333	NM_001104648	Vmn2r56	0	1	1	0	1	1	1	0	none
chr7:13385336..13385357-	chr7	-	13385336	13385357	0,313968788	66381	NM_001104648	Vmn2r56	0	1	1	0	1	1	1	0	none
chr7:13385381..13385382-	chr7	-	13385381	13385382	0,04830289	66426	NM_001104648	Vmn2r56	0	1	1	0	1	1	1	0	none
chr7:13385460..13385460-	chr7	-	13385460	13385460	0,024151445	66505	NM_001104648	Vmn2r56	0	1	1	0	1	1	1	0	none
chr7:13385928..13385930-	chr7	-	13385928	13385930	0,579634685	66975	NM_001104648	Vmn2r56	1	1	1	0	1	1	0	SINE	
chr7:13385985..13385985-	chr7	-	13385985	13385985	0,024151445	67030	NM_001104648	Vmn2r56	1	1	1	0	1	1	0	LINE	
chr7:13386342..13386342-	chr7	-	13386342	13386342	0,072454336	67387	NM_001104648	Vmn2r56	0	1	1	0	1	1	0	none	
chr7:13386400..13386400-	chr7	-	13386400	13386400	0,193211562	67445	NM_001104648	Vmn2r56	0	1	1	0	1	1	0	none	
chr7:13386576..13386576-	chr7	-	13386576	13386576	0,289817343	67621	NM_001104648	Vmn2r56	1	1	1	0	1	1	0	Simple_repeat	
chr7:13386637..13386640-	chr7	-	13386637	13386640	1,424953268	67684	NM_001104648	Vmn2r56	0	1	1	0	1	1	0	none	
chr7:13387322..13387322-	chr7	-	13387322	13387322	0,120757226	68367	NM_001104648	Vmn2r56	0	1	1	0	1	1	0	LTR	
chr7:13388611..13388611-	chr7	-	13388611	13388611	0,120757226	69656	NM_001104648	Vmn2r56	0	1	1	0	0	1	0	none	
chr7:13388759..13388759-	chr7	-	13388759	13388759	0,652089021	69804	NM_001104648	Vmn2r56	0	1	1	0	0	1	0	none	
chr7:13388943..13388991-	chr7	-	13388943	13388991	1,231723707	69991	NM_001104648	Vmn2r56	0	1	1	0	0	0	1	none	
chr7:48706663..48706663-	chr7	-	48706663	48706663	0,024151445	2151	NM_177764	Vmn2r57	0	0	0	0	0	0	0	LINE	
chr7:48715254..48715254-	chr7	-	48715254	48715254	0,096605781	10742	NM_177764	Vmn2r57	0	0	0	0	0	0	0	none	
chr7:48722983..48722983-	chr7	-	48722983	48722983	0,052831286	18471	NM_177764	Vmn2r57	0	1	0	0	0	1	0	none	
chr7:48723019..48723080-	chr7	-	48723019	48723080	0,327361067	18552	NM_177764	Vmn2r57	0	1	0	0	0	1	0	none	
chr7:48723134..48723157-	chr7	-	48723134	48723157	0,122176653	18645	NM_177764	Vmn2r57	0	1	0	0	0	1	0	none	
chr7:48727513..48727513-	chr7	-	48727513	48727513	0,024151445	23001	NM_177764	Vmn2r57	1	0	0	0	0	1	0	LINE	
chr7:48730432..48730434-	chr7	-	48730432	48730434	0,410574569	25922	NM_177764	Vmn2r57	1	0	0	0	0	0	0	LINE	
chr7:48731792..48731793-	chr7	-	48731792	48731793	0,748694802	27281	NM_177764	Vmn2r57	1	0	0	0	0	0	0	LINE	
chr7:48734114..48734114-	chr7	-	48734114	48734114	0,024151445	29602	NM_177764	Vmn2r57	1	1	0	0	0	0	0	SINE	
chr7:49145380..49145380-	chr7	-	49145380	49145380	0,144908671	16839	NM_001105055	Vmn2r58	1	0	0	0	0	0	0	LINE	
chr7:49161085..49161085-	chr7	-	49161085	49161085	0,024151445	32544	NM_001105055	Vmn2r58	1	0	0	0	0	0	0	LINE	
chr7:49174520..49174520-	chr7	-	49174520	49174520	0,04830289	45979	NM_001105055	Vmn2r58	0	0	0	0	0	0	0	none	
chr7:49198835..49198835-	chr7	-	49198835	49198835	0,024151445	70294	NM_001105055	Vmn2r58	1	0	0	0	0	0	0	LINE	
chr7:49217798..49217798-	chr7	-	49217798	49217798	0,096605781	89257	NM_001105055	Vmn2r58	1	0	0	0	0	0	0	LINE	
chr7:49219168..49219168-	chr7	-	49219168	49219168	0,144908671	90627	NM_001105055	Vmn2r58	0	1	0	0	0	0	0	LINE	
chr7:49234969..49234969-	chr7	-	49234969	49234969	0,024151445	106428	NM_001105055	Vmn2r58	0	1	0	0	0	0	0	none	
chr7:49250216..49250217-	chr7	-	49250216	49250217	0,07513783	121676	NM_001105055	Vmn2r58	0	1	0	0	0	0	0	LINE	
chr7:49262638..49262638-	chr7	-	49262638	49262638	0,024151445	134097	NM_001105055	Vmn2r58	0	1	0	0	0	0	0	LINE	
chr7:49315243..49315243+	chr7	+	49315243	49315243	0,217363007	56597	NM_001105057	Vmn2r60	0	1	0	0	0	0	0	none	
chr7:49316685..49316685-	chr7	-	49316685	49316685	0,024151445	1833	NM_001105056	Vmn2r59	0	0	0	0	0	0	1	none	
chr7:49342948..49342948+	chr7	+	49342948	49342948	0,024151445	28892	NM_001105057	Vmn2r60	1	0	0	0	0	0	0	LINE	
chr7:49344532..49344532-	chr7	-	49344532	49344532	0,024151445	29680	NM_001105056	Vmn2r59	1	0	0	0	0	0	0	LTR	
chr7:49354264..49354264-	chr7	-	49354264	49354264	0,096605781	39412	NM_001105056	Vmn2r59	0	0	0	0	0	0	0	LINE	
chr7:49357072..49357072-	chr7	-	49357072	49357072	0,120757226	42220	NM_001105056	Vmn2r59	0	0	0	0	0	0	0	none	
chr7:49359379..49359379-	chr7	-	49359379	49359379	0,096605781	44527	NM_001105056	Vmn2r59	0	0	0	0	0	0	0	LINE	
chr7:49362536..49362552-	chr7	-	49362536	49362552	0,362271678	47700	NM_001105056	Vmn2r59	0	0	0	0	0	1	1	none	
chr7:49362582..49362636-	chr7	-	49362582	49362636	0,049811481	47775	NM_001105056	Vmn2r59	0	0	0	0	0	0	0	none	
chr7:49366407..49366407-	chr7	-	49366407	49366407	0,024151445	51555	NM_001105056	Vmn2r59	1	1	0	0	0	0	0	LTR	
chr7:49368496..49368496+	chr7	+	49368496	49368496	0,024151445	3344	NM_001105057	Vmn2r60	0	1	0	0	0	0	1	none	
chr7:49369770..49369770+	chr7	+	49369770	49369770	0,024151445	2070	NM_001105057	Vmn2r60	1	0	0	0	0	0	0	LINE	
chr7:49369864..49369864-	chr7	-	49369864	49369864	0,024151445	55012	NM_001105056	Vmn2r59	0	1	0	0	0	0	0	LINE	
chr7:49452473..49452473+	chr7	+	49452473	49452473	0,024151445	62949	NM_001105058	Vmn2r61	1	1	0	0	0	1	0	LINE	
chr7:49471310..49471310+	chr7	+	49471310	49471310	0,024151445	44112	NM_001105058	Vmn2r61	0	0	0	0	0	0	0	none	
chr7:49508262..49508262+	chr7	+	49508262	49508262	0,169060117	7160	NM_001105058	Vmn2r61	1	0	0	0	0	0	0	LTR	
chr7:49508920..49508920+	chr7	+	49508920	49508920	0,096605781	6502	NM_001105058	Vmn2r61	0	0	0	0	0	0	0	LTR	
chr7:50082167..50082167-	chr7	-	50082167	50082167	0,024151445	32800	NM_001105059	Vmn2r62	0	0	0	0	0	0	0	none	
chr7:50097699..50097699-	chr7	-	50097699	50097699	0,024151445	48332	NM_001105059	Vmn2r62	1	0	0	0	0	0	0	LINE	
chr7:50112511..50112511-	chr7	-	50112511	50112511	0,024151445	63144	NM_001105059	Vmn2r62	0	1	0	0	0	0	0	none	
chr7:50112928..50112928-	chr7	-	50112928	50112928	0,096605781	63561	NM_001105059	Vmn2r62	0	1	0	0	0	0	0	none	
chr7:50117567..50117567-	chr7	-	50117567	50117567	0,120757226	68200	NM_001105059	Vmn2r62	1	1	0	0	0	0	0	Simple_repeat	
chr7:50151658..50151660-	chr7	-	50151658	50151660	0,120757226	102293	NM_001105059	Vmn2r62	1	0	0	0	0	0	0	LTR	
chr7:50153299..50153299-	chr7	-	50153299	50153299	0,603786131	103932	NM_001105059	Vmn2r62	1	0	0	0	0	0	0	LTR	
chr7:50199956..50199959-	chr7	-	50199956	50199959	0,169060117	10299	NM_001105060	Vmn2r63	0	0	0	0	0	0	0	LINE	
chr7:50201312..50201312-	chr7	-	50201312	50201312	0,096605781	11652	NM_001105060	Vmn2r63	1	0	0	0	0	0	0	LINE	
chr7:5468721..5468721-	chr7	-	5468721	5468721	0,144908671	22767	NM_001081405	Vmn2r28	1	0	0	0	0	0	0	LINE	
chr7:5487761..5487761-	chr7	-	5487761	5487761	0,217363007	41807	NM_001081405	Vmn2r28	1	0	0	0	0	0	0	LINE	
chr7:5553028..5553028-	chr7	-	5553028	5553028	0,072454336	107074	NM_001081405	Vmn2r28	0	0	0	0	0	0	0	none	
chr7:5590183..5590183-	chr7	-	5590183	5590183	0,144908671	144229	NM_001081405	Vmn2r28	1	0	0	0	0	0	0	LINE	
chr7:7205630..7205630-	chr7	-	7205630	7205630	0,024151445	5090	NM_001113468	Vmn2r29	0	0	0	0	0	0	0	LINE	
chr7:7215150..7215150-	chr7	-	7215150	7215150	0,096605781	14610	NM_001113468	Vmn2r29	1	0	0	0	0	0	0	LINE	
chr7:7219979..7219993-	chr7	-	7219979	7219993	1,497389604	19439	NM_001113468	Vmn2r29	0	1	0	0	0	1	0	none	
chr7:7223256..7223256-	chr7	-	7223256	7223256	0,362271678	22716	NM_001113468	Vmn2r29	0	1	0	0	0	0	0	none	
chr7:7223844..7223848-	chr7	-	7223844	7223848	0,169060117	23308	NM_001113468	Vmn2r29	0	0	0	0	0	0	0	LTR	
chr7:7224025..7224025-	chr7	-	7224025	7224025	0,024151445	23485	NM_001113468	Vmn2r29	1	0	0	0	0	0	0	LTR	
chr7:7224120..7224120-	chr7																

chr7:7224517..7224517	-	7224517	7224517	0,072454336	23977	NM_001113468	Vmn2r29	0	0	0	0	0	0	0	0	0	none
chr7:7230856..7230856	-	7230856	7230856	0,120757226	30316	NM_001113468	Vmn2r29	0	0	0	0	0	0	0	0	0	none
chr7:7230915..7230915	-	7230915	7230915	0,338120233	30375	NM_001113468	Vmn2r29	0	0	0	0	0	0	0	0	0	none
chr7:7230948..7231020	-	7230948	7231020	0,763683091	30441	NM_001113468	Vmn2r29	0	0	0	0	0	0	0	0	0	none
chr7:7309860..7309860	-	7309860	7309860	0,024151445	19155	NM_009490	Vmn2r30	1	0	0	0	0	0	0	0	0	LTR
chr7:7318223..7318223	-	7318223	7318223	0,024151445	27518	NM_009490	Vmn2r30	1	0	0	0	0	0	1	0	0	LINE
chr7:7327343..7327343	-	7327343	7327343	0,169060117	36638	NM_009490	Vmn2r30	0	0	0	0	0	0	1	0	0	none
chr7:7714010..7714010	-	7714010	7714010	0,024151445	71400	NM_001105066	Vmn2r34	1	0	0	0	0	0	0	0	0	LINE
chr7:7732848..7732848	-	7732848	7732848	0,024151445	90238	NM_001105066	Vmn2r34	1	0	0	0	0	0	0	0	0	LTR
chr7:8890147..8890150	+	8890147	8890150	0,030189307	64224	NM_009489	Vmn2r37	1	0	0	0	0	0	0	0	0	LINE
chr7:8921708..8921708	+	8921708	8921708	0,024151445	32663	NM_009489	Vmn2r37	1	0	0	0	0	0	0	0	0	LTR
chr7:9062370..9062370	+	9062370	9062370	0,289817343	17651	NM_001105070	Vmn2r38	0	0	0	0	0	0	0	0	0	none
chr7:92134548..92134548	-	92134548	92134548	0,024151445	21528	NM_001105180	Vmn2r65	0	0	0	0	0	0	0	0	0	none
chr7:92134618..92134640	-	92134618	92134640	0,772846247	21598	NM_001105180	Vmn2r65	0	0	0	0	0	0	0	0	0	none
chr7:92134704..92134704	-	92134704	92134704	0,024151445	21684	NM_001105180	Vmn2r65	0	0	0	0	0	0	0	0	0	none
chr7:92134984..92134984	-	92134984	92134984	0,012075723	21964	NM_001105180	Vmn2r65	0	0	0	0	0	0	0	0	0	none
chr7:92135023..92135035	-	92135023	92135035	3,332899441	22004	NM_001105180	Vmn2r65	0	0	0	0	0	0	1	0	0	none
chr7:92141283..92141283	-	92141283	92141283	0,169060117	28263	NM_001105180	Vmn2r65	0	0	0	0	0	0	1	0	0	none
chr7:92143122..92143122	-	92143122	92143122	0,096605781	30102	NM_001105180	Vmn2r65	0	0	0	0	0	0	1	1	0	none
chr7:92179230..92179230	-	92179230	92179230	0,120757226	18199	NM_001033878	Vmn2r66	1	0	0	0	0	0	0	0	0	LINE
chr7:92193851..92193851	-	92193851	92193851	0,024151445	32820	NM_001033878	Vmn2r66	1	0	0	0	0	0	1	0	0	LINE
chr7:92200157..92200157	-	92200157	92200157	0,169060117	39126	NM_001033878	Vmn2r66	1	0	0	0	0	0	0	0	0	LTR
chr7:92208044..92208044	-	92208044	92208044	0,024151445	47013	NM_001033878	Vmn2r66	1	0	0	0	0	0	0	0	0	LINE
chr7:92211281..92211281	-	92211281	92211281	0,024151445	50250	NM_001033878	Vmn2r66	1	0	0	0	0	0	0	0	0	LINE
chr7:92217556..92217556	-	92217556	92217556	0,024151445	56525	NM_001033878	Vmn2r66	1	0	0	0	0	0	1	0	0	LINE
chr7:92218820..92218820	-	92218820	92218820	0,024151445	57789	NM_001033878	Vmn2r66	1	0	0	0	0	0	1	1	0	none
chr7:92224701..92224701	-	92224701	92224701	0,144908671	63670	NM_001033878	Vmn2r66	1	0	0	0	0	0	0	0	0	LINE
chr7:92239313..92239313	-	92239313	92239313	0,144908671	78282	NM_001033878	Vmn2r66	0	0	0	0	0	0	1	1	0	none
chr7:92273673..92273673	-	92273673	92273673	0,024151445	112642	NM_001033878	Vmn2r66	1	0	0	0	0	0	0	0	0	LINE
chr7:92274542..92274542	-	92274542	92274542	0,265665897	113511	NM_001033878	Vmn2r66	1	0	0	0	0	0	0	0	0	LINE
chr7:92318472..92318472	-	92318472	92318472	0,024151445	13559	NM_001102579	Vmn2r67	1	0	0	0	0	0	0	0	0	LINE
chr7:92319762..92319762	-	92319762	92319762	0,024151445	14849	NM_001102579	Vmn2r67	1	0	0	0	0	0	0	0	0	LINE
chr7:92365837..92365837	-	92365837	92365837	0,024151445	60924	NM_001102579	Vmn2r67	1	0	0	0	0	0	0	0	0	LINE
chr7:92396474..92396474	-	92396474	92396474	0,169060117	9759	NM_001105181	Vmn2r68	1	0	0	0	0	0	0	0	0	LINE
chr7:92411214..92411214	-	92411214	92411214	0,144908671	24499	NM_001105181	Vmn2r68	1	0	0	0	0	0	0	0	0	LINE
chr7:92423548..92423548	-	92423548	92423548	0,120757226	36833	NM_001105181	Vmn2r68	1	0	0	0	0	0	0	0	0	LINE
chr7:92427914..92427915	-	92427914	92427915	0,020126204	41200	NM_001105181	Vmn2r68	1	0	0	0	0	0	0	0	0	LTR
chr7:92427999..92427999	-	92427999	92427999	0,024151445	41284	NM_001105181	Vmn2r68	1	0	0	0	0	0	0	0	0	LTR
chr7:92448372..92448372	-	92448372	92448372	0,024151445	61657	NM_001105181	Vmn2r68	1	0	0	0	0	0	0	0	0	LTR
chr7:92453738..92453738	-	92453738	92453738	0,024151445	67023	NM_001105181	Vmn2r68	1	0	0	0	0	0	1	0	0	none
chr7:92454493..92454493	-	92454493	92454493	0,024151445	67778	NM_001105181	Vmn2r68	1	0	0	0	0	0	1	0	0	Low_complexity
chr7:92455621..92455621	-	92455621	92455621	0,024151445	68906	NM_001105181	Vmn2r68	0	0	0	0	0	0	1	0	0	none
chr7:92456920..92456920	-	92456920	92456920	0,120757226	70205	NM_001105181	Vmn2r68	0	0	0	0	0	0	1	1	0	none
chr7:92458550..92458550	-	92458550	92458550	0,096605781	71835	NM_001105181	Vmn2r68	0	0	0	0	0	0	1	1	0	none
chr7:92471731..92471731	-	92471731	92471731	0,024151445	85016	NM_001105181	Vmn2r68	1	0	0	0	0	0	0	0	0	LINE
chr7:92477263..92477263	-	92477263	92477263	0,096605781	90548	NM_001105181	Vmn2r68	1	0	0	0	0	0	0	0	0	LINE
chr7:92498784..92498784	-	92498784	92498784	0,072454336	112069	NM_001105181	Vmn2r68	1	0	0	0	0	0	0	0	0	LINE
chr7:92527001..92527001	-	92527001	92527001	0,024151445	140286	NM_001105181	Vmn2r68	1	0	0	0	0	0	0	0	0	LTR
chr7:92531030..92531030	-	92531030	92531030	0,024151445	144315	NM_001105181	Vmn2r68	1	0	0	0	0	0	0	0	0	LINE
chr7:92534726..92534726	-	92534726	92534726	0,072454336	148011	NM_001105181	Vmn2r68	1	0	0	0	0	0	0	0	0	LINE
chr7:92576261..92576261	-	92576261	92576261	0,144908671	11574	NM_001105182	Vmn2r69	1	0	0	0	0	0	0	0	0	LINE
chr7:92577351..92577351	-	92577351	92577351	0,144908671	12664	NM_001105182	Vmn2r69	1	0	0	0	0	0	0	0	0	LINE
chr7:92577760..92577760	-	92577760	92577760	0,024151445	13073	NM_001105182	Vmn2r69	1	0	0	0	0	0	0	0	0	LINE
chr7:92578181..92578181	-	92578181	92578181	0,024151445	13494	NM_001105182	Vmn2r69	1	0	0	0	0	0	0	0	0	Simple_repeat
chr7:92584121..92584121	-	92584121	92584121	0,169060117	19434	NM_001105182	Vmn2r69	1	0	0	0	0	0	0	0	0	LINE
chr7:92593210..92593210	-	92593210	92593210	0,108681504	28523	NM_001105182	Vmn2r69	1	0	0	0	0	0	0	0	0	LINE
chr7:92598820..92598820	-	92598820	92598820	0,217363007	34133	NM_001105182	Vmn2r69	0	1	0	0	0	0	0	0	0	none
chr7:92599837..92599840	-	92599837	92599840	1,159269371	35153	NM_001105182	Vmn2r69	0	0	1	0	0	0	0	0	0	none
chr7:92637693..92637693	-	92637693	92637693	0,120757226	73006	NM_001105182	Vmn2r69	1	0	0	0	0	0	0	0	0	LTR
chr7:92654324..92654324	-	92654324	92654324	0,072454336	89637	NM_001105182	Vmn2r69	1	0	0	0	0	0	0	0	0	LTR
chr7:92663322..92663323	-	92663322	92663323	0,289817343	88636	NM_001105182	Vmn2r69	1	0	0	0	0	0	0	0	0	LINE
chr7:92669536..92669536	-	92669536	92669536	0,169060117	104849	NM_001105182	Vmn2r69	0	0	0	0	0	0	0	0	0	none
chr7:92686901..92686901	-	92686901	92686901	0,072454336	122214	NM_001105182	Vmn2r69	0	0	0	0	0	0	0	0	0	none
chr7:92687692..92687692	-	92687692	92687692	0,024151445	123005	NM_001105182	Vmn2r69	1	0	0	0	0	0	0	0	0	LINE
chr7:92689888..92689888	-	92689888	92689888	0,024151445	125201	NM_001105182	Vmn2r69	0	0	0	0	0	0	0	0	0	none
chr7:92698006..92698006	-	92698006	92698006	0,024151445	133319	NM_001105182	Vmn2r69	1	0	0	0	0	0	0	0	0	LINE
chr7:92703387..92703387	-	92703387	92703387	0,072454336	138700	NM_001105182	Vmn2r69	1	0	0	0	0	0	0	0	0	LINE
chr7:92705565..92705565	-	92705565	92705565	0,024151445	140878	NM_001105182	Vmn2r69	0	0	0	0	0	0	1	1	0	none
chr7:92707103..92707103	-	92707103	92707103	2,729113311	142416	NM_001105182	Vmn2r69	0	0	0	0	0	0	1	1	0	none
chr7:92722540..92722540	-	92722540	92722540	0,024151445	4441	NM_001105183	Vmn2r70	1	0	0	0	0	0	0	0	0	LINE
chr7:92724503..92724503	-	92724503	92724503	0,024151445	6404	NM_001105183	Vmn2r70	1	0	0	0	0	0	0	0	0	LINE
chr7:92724874..92724874	+	92724874	92724874	0,024151445	39097	NM_001105184	Vmn2r71	1	0	0	0	0	0	0	0	0	LINE
chr7:92739431..92739431+	+	92739431															

chr7:92757102..92757114-	chr7	-	92757102	92757114	0,265665897	39003	NM_001105183	Vmn2r70	0	0	0	0	0	0	0	0	0	none
chr7:92757153..92757153+	chr7	+	92757153	92757153	0,024151445	6818	NM_001105184	Vmn2r71	1	0	0	0	0	0	0	0	0	LINE
chr7:92918579..92918579-	chr7	-	92918579	92918579	0,048302089	14587	NM_001105185	Vmn2r72	1	0	0	0	0	0	0	0	0	LTR
chr7:92927657..92927657-	chr7	-	92927657	92927657	0,072454336	23665	NM_001105185	Vmn2r72	1	0	0	0	0	0	0	0	0	LINE
chr7:92929025..92929025-	chr7	-	92929025	92929025	0,048302089	25033	NM_001105185	Vmn2r72	1	0	0	0	0	0	0	0	0	LINE
chr7:92941313..92941313-	chr7	-	92941313	92941313	0,024151445	37321	NM_001105185	Vmn2r72	0	0	0	0	0	0	0	0	0	none
chr7:92955895..92955895-	chr7	-	92955895	92955895	0,024151445	51903	NM_001105185	Vmn2r72	1	0	0	0	0	0	0	0	0	LINE
chr7:92968595..92968596-	chr7	-	92968595	92968596	0,483028905	64604	NM_001105185	Vmn2r72	1	0	0	0	0	0	0	0	0	LINE
chr7:92983018..92983018-	chr7	-	92983018	92983018	0,024151445	79026	NM_001105185	Vmn2r72	1	0	0	0	0	0	0	0	0	LINE
chr7:93005986..93005986-	chr7	-	93005986	93005986	0,048302089	101994	NM_001105185	Vmn2r72	0	0	0	0	0	0	1	1	0	none
chr7:93036513..93036513-	chr7	-	93036513	93036513	0,024151445	11564	NM_001105186	Vmn2r73	1	0	0	0	0	0	0	0	0	LTR
chr7:93062805..93062805-	chr7	-	93062805	93062805	0,048302089	37856	NM_001105186	Vmn2r73	0	0	0	0	0	0	0	0	0	none
chr7:93068735..93068735-	chr7	-	93068735	93068735	0,144908671	43786	NM_001105186	Vmn2r73	0	0	0	0	0	0	0	0	0	none
chr7:93088055..93088055-	chr7	-	93088055	93088055	0,048302089	63106	NM_001105186	Vmn2r73	0	1	0	0	0	0	0	0	0	none
chr7:93093669..93093669-	chr7	-	93093669	93093669	0,024151445	68720	NM_001105186	Vmn2r73	1	0	0	0	0	0	0	0	0	LINE
chr7:93095017..93095017-	chr7	-	93095017	93095017	0,024151445	70068	NM_001105186	Vmn2r73	0	1	0	0	0	0	0	0	0	none
chr7:93098448..93098449-	chr7	-	93098448	93098449	0,169060117	73500	NM_001105186	Vmn2r73	0	1	0	0	0	0	0	0	0	none
chr7:93100367..93100367-	chr7	-	93100367	93100367	0,024151445	75418	NM_001105186	Vmn2r73	0	0	0	0	0	0	1	1	0	none
chr7:93113710..93113710-	chr7	-	93113710	93113710	0,096605781	3217	NM_001105187	Vmn2r74	1	0	0	0	0	0	0	0	1	LINE
chr7:93114260..93114260-	chr7	-	93114260	93114260	0,024151445	3767	NM_001105187	Vmn2r74	1	0	0	0	0	0	0	0	0	LINE
chr7:93128021..93128021-	chr7	-	93128021	93128021	0,024151445	17528	NM_001105187	Vmn2r74	0	0	0	0	0	0	0	0	0	none
chr7:93140398..93140398-	chr7	-	93140398	93140398	0,024151445	29905	NM_001105187	Vmn2r74	1	1	0	0	0	0	0	0	0	LINE
chr7:93146975..93146975-	chr7	-	93146975	93146975	0,120757226	36482	NM_001105187	Vmn2r74	0	1	0	0	0	0	1	0	0	none
chr7:93165846..93165846-	chr7	-	93165846	93165846	0,024151445	55353	NM_001105187	Vmn2r74	0	0	0	0	0	0	1	0	0	none
chr7:93167931..93167931-	chr7	-	93167931	93167931	0,096605781	57438	NM_001105187	Vmn2r74	1	0	0	0	0	0	0	0	0	LINE
chr7:93211689..93211689-	chr7	-	93211689	93211689	0,024151445	101196	NM_001105187	Vmn2r74	0	0	0	0	0	0	1	0	0	none
chr7:93216259..93216259-	chr7	-	93216259	93216259	0,072454336	105766	NM_001105187	Vmn2r74	1	0	0	0	0	0	0	0	0	LINE
chr7:93227809..93227809-	chr7	-	93227809	93227809	0,024151445	117316	NM_001105187	Vmn2r74	1	0	0	0	0	0	0	0	0	LINE
chr7:93228686..93228688-	chr7	-	93228686	93228688	0,579636685	118195	NM_001105187	Vmn2r74	1	1	0	0	0	0	0	0	0	LINE
chr7:93266747..93266747-	chr7	-	93266747	93266747	0,072454336	156254	NM_001105187	Vmn2r74	1	0	0	0	0	0	0	0	0	LINE
chr7:93280835..93280835-	chr7	-	93280835	93280835	0,120757226	170342	NM_001105187	Vmn2r74	0	0	0	0	0	0	0	0	0	none
chr7:93290022..93290022-	chr7	-	93290022	93290022	0,217363007	179529	NM_001105187	Vmn2r74	0	0	0	0	0	0	0	0	0	none
chr7:93296194..93296194-	chr7	-	93296194	93296194	0,241514452	185701	NM_001105187	Vmn2r74	0	0	0	0	0	0	1	1	0	none
chr7:93325392..93325392-	chr7	-	93325392	93325392	0,024151445	4657	NM_001102578	Vmn2r75	1	0	0	0	0	0	0	0	0	LINE
chr7:93352605..93352605-	chr7	-	93352605	93352605	0,024151445	31870	NM_001102578	Vmn2r75	1	0	0	0	0	0	0	0	0	LINE
chr7:93353869..93353869-	chr7	-	93353869	93353869	0,024151445	33134	NM_001102578	Vmn2r75	1	0	0	0	0	0	0	0	0	LINE
chr7:93358076..93358076-	chr7	-	93358076	93358076	0,048302089	37341	NM_001102578	Vmn2r75	1	0	0	0	0	0	0	0	0	LTR
chr7:93360336..93360336-	chr7	-	93360336	93360336	0,096605781	39601	NM_001102578	Vmn2r75	1	0	0	0	0	0	0	0	0	LTR
chr7:93413138..93413142-	chr7	-	93413138	93413142	1,135117926	17930	NM_001102580	Vmn2r76	0	0	0	0	0	0	1	1	0	none
chr7:93415956..93415958-	chr7	-	93415956	93415958	0,289817343	20744	NM_001102580	Vmn2r76	1	0	0	0	0	0	0	0	0	LTR
chr7:93938348..93938348+	chr7	+	93938348	93938348	0,072454336	5302	NM_001105188	Vmn2r77	1	0	0	0	0	0	0	0	0	LINE
chr7:93967051..93967051+	chr7	+	93967051	93967051	0,217363007	96807	NM_001105189	Vmn2r78	1	0	0	0	0	0	0	0	0	none
chr7:93970786..93970786+	chr7	+	93970786	93970786	0,072454336	93072	NM_001105189	Vmn2r78	1	0	0	0	0	0	0	0	0	LINE
chr7:93975943..93975943+	chr7	+	93975943	93975943	0,024151445	87915	NM_001105189	Vmn2r78	1	0	0	0	0	0	0	0	0	LINE
chr7:94007823..94007823+	chr7	+	94007823	94007823	0,048302089	56035	NM_001105189	Vmn2r78	0	1	0	0	0	0	1	0	0	none
chr7:94025820..94025820+	chr7	+	94025820	94025820	0,024151445	38038	NM_001105189	Vmn2r78	1	0	0	0	0	0	0	0	0	LTR
chr7:94025863..94025863+	chr7	+	94025863	94025863	0,169060117	37995	NM_001105189	Vmn2r78	1	0	0	0	0	0	0	0	0	LTR
chr7:94034188..94034188+	chr7	+	94034188	94034188	0,024151445	29670	NM_001105189	Vmn2r78	0	1	0	0	0	0	1	1	0	none
chr7:94038597..94038597+	chr7	+	94038597	94038597	0,120757226	25261	NM_001105189	Vmn2r78	0	0	0	0	0	0	0	0	0	none
chr7:94057110..94057110+	chr7	+	94057110	94057110	0,265665897	6748	NM_001105189	Vmn2r78	1	0	0	0	0	0	0	0	0	LINE
chr7:94058536..94058537+	chr7	+	94058536	94058537	0,072454336	5322	NM_001105189	Vmn2r78	0	0	0	0	0	0	0	0	0	LINE
chr7:94114836..94114836+	chr7	+	94114836	94114836	0,048302089	30138	NM_001105190	Vmn2r79	0	1	0	0	0	0	0	0	0	none
chr7:94115885..94115885+	chr7	+	94115885	94115885	0,096605781	29089	NM_001105190	Vmn2r79	1	0	0	0	0	0	0	0	0	LINE
chr7:94128447..94128447+	chr7	+	94128447	94128447	0,120757226	16527	NM_001105190	Vmn2r79	1	0	0	0	0	0	0	0	0	LINE
chr7:9670309..9670309+	chr7	+	9670309	9670309	0,120757226	73770	NM_001105074	Vmn2r44	1	0	0	0	0	0	1	0	0	LTR
chr7:9772104..9772104+	chr7	+	9772104	9772104	0,024151445	94656	NM_198961	Vmn2r43	0	0	0	0	0	0	0	0	0	none
chrX:121256840..121256840-	chrX	-	121256840	121256840	0,072454336	7399	NM_001100616	Vmn2r121	0	0	0	0	0	0	0	0	0	none
chrX:121296764..121296764-	chrX	-	121296764	121296764	0,024151445	47323	NM_001100616	Vmn2r121	1	0	0	0	0	0	0	0	0	LINE
chrX:121318062..121318062-	chrX	-	121318062	121318062	0,024151445	68621	NM_001100616	Vmn2r121	1	1	0	0	0	0	0	0	0	Satellite
chrX:121324316..121324316-	chrX	-	121324316	121324316	0,024151445	74875	NM_001100616	Vmn2r121	1	0	0	0	0	0	0	0	0	none
chrX:121339027..121339027-	chrX	-	121339027	121339027	0,072454336	89586	NM_001100616	Vmn2r121	0	0	0	0	0	0	0	0	0	SINE
chrX:121340562..121340562-	chrX	-	121340562	121340562	0,024151445	91121	NM_001100616	Vmn2r121	1	0	0	0	0	0	0	0	0	LINE
chrX:121351455..121351455-	chrX	-	121351455	121351455	0,024151445	102014	NM_001100616	Vmn2r121	1	0	0	0	0	0	0	0	0	Simple_repeat

Supplementary Table S2 (next page).

List of all V1Rs and V2Rs genes having at least one nanoCAGE TC mapping 5’ upstream of the annotated Refseq. Lower panel, table displaying the total nanoCAGE tags count for housekeeping genes and for the most known genes involved in the signal transduction pathways of ORs and VRs.

List of all V1Rs and V2Rs genes downstream of nanoCAGE TCs

V1Rs genes

V1ra1
V1ra2
V1ra4
V1ra5
V1ra6
V1ra7
V1ra8
V1ra9
V1rb1
V1rb3
V1rb4
V1rb7
V1rb8
V1rb10
V1rc1
V1rc2
V1rc3
V1rc5
V1rc6
V1rc7
V1rc8
V1rc11
V1rc12
V1rc13
V1rc14
V1rc15
V1rc16
V1rc17
V1rc18
V1rc19
V1rc21
V1rc22
V1rc24
V1rc25
V1rc26
V1rc27
V1rc28
V1rc29
V1rc30
V1rc32
V1rd1
V1rd3
V1rd4
V1rd6
V1rd7
V1rd8
V1rd9
V1rd11
V1rd12
V1rd13
V1rd15

V2Rs genes

Vmn2r1
Vmn2r2
Vmn2r3
Vmn2r4
Vmn2r6
Vmn2r7
Vmn2r8
Vmn2r9
Vmn2r10
Vmn2r12
Vmn2r13
Vmn2r14
Vmn2r15
Vmn2r16
Vmn2r17
Vmn2r18
Vmn2r19
Vmn2r22
Vmn2r23
Vmn2r24
Vmn2r25
Vmn2r26
Vmn2r27
Vmn2r28
Vmn2r29
Vmn2r30
Vmn2r34
Vmn2r37
Vmn2r38
Vmn2r43
Vmn2r44
Vmn2r47
Vmn2r49
Vmn2r51
Vmn2r52
Vmn2r53
Vmn2r56
Vmn2r57
Vmn2r58
Vmn2r59
Vmn2r60
Vmn2r61
Vmn2r62
Vmn2r63
Vmn2r65
Vmn2r66
Vmn2r67
Vmn2r68
Vmn2r69
Vmn2r70
Vmn2r71

V1rd16	Vmn2r72
V1rd17	Vmn2r73
V1rd19	Vmn2r74
V1rd20	Vmn2r75
V1rd22	Vmn2r76
V1re1	Vmn2r77
V1re2	Vmn2r78
V1re3	Vmn2r79
V1re4	Vmn2r80
V1re5	Vmn2r81
V1re6	Vmn2r82
V1re7	Vmn2r83
V1re8	Vmn2r85
V1re9	Vmn2r86
V1re10	Vmn2r87
V1re11	Vmn2r90
V1re12	Vmn2r91
V1re13	Vmn2r92
V1rf1	Vmn2r93
V1rf3	Vmn2r94
V1rf4	Vmn2r95
V1rf5	Vmn2r96
V1rg1	Vmn2r97
V1rg2	Vmn2r98
V1rg3	Vmn2r99
V1rg4	Vmn2r100
V1rg5	Vmn2r101
V1rg6	Vmn2r102
V1rg7	Vmn2r103
V1rg8	Vmn2r104
V1rg9	Vmn2r105
V1rg11	Vmn2r106
V1rg12	Vmn2r107
V1rh2	Vmn2r108
V1rh3	Vmn2r109
V1rh4	Vmn2r110
V1rh5	Vmn2r111
V1rh6	Vmn2r112
V1rh7	Vmn2r113
V1rh8	Vmn2r114
V1rh9	Vmn2r115
V1rh10	Vmn2r116
V1rh11	Vmn2r117
V1rh12	Vmn2r118
V1rh13	Vmn2r120
V1rh14	Vmn2r121
V1rh17	
V1rh18	
V1rh20	
V1rh21	
V1ri2	
V1ri3	
V1ri4	
V1ri5	

V1ri6
V1ri7
V1ri8
V1ri10
V1rj2
V1rj3
V1rk1
V1rl1

NanoCAGE tags count for housekeeping genes and genes involved in Ors and VRs signal transduction pathways.

Gene name (Refseq)	Tag Count
V1Rs loci (see Table 1, 2, 3)	577
V2Rs loci (see Table 1, 2, 3)	812
Ors in CLICs (ref. Plessy et al, 2012)	26,452
Beta-actin (Actb)	8,579
Ubiquitin (Ubb)	8,453
Tubulin, alpha 1A (Tuba1a)	74,465
Omp	6,342
Adenylate cyclase III (Adcy3)	604
Cnga2	10,956
Gαolf (Gnal)	3,489
Trpc2, isoform 1 (NM_011644)	261
Trpc2, isoform 2 (NM_001109897)	243
Gαo (Gnao1)	544
Gαi2 (Gnai2)	64

Supplementary Table S3 (next page).

Classification of all LINE1 elements associated with V1Rs and V2Rs.

Expressed LINE1s classification - V1Rs loci

Genomic Coordinates	Chromosome	strand	TPM	Distance to annotated TSS	RefseqID of associated VR	Gene symbol	Repeat Class/family	annotation.rmssk.tss_distance
chr7:13733534..13733534+	chr7	-	0,02415145	15826	NM_134227	V1rk1	L1Md_A	208
chr7:13733600..13733600+	chr7	-	0,02415145	15892	NM_134227	V1rk1	L1Md_A	142
chr7:13733662..13733662+	chr7	-	0,07245434	15954	NM_134227	V1rk1	L1Md_A	80
chr7:5395109..5395109+	chr7	-	0,02415145	32277	NM_030739	V1rd4	L1Md_A	-2207
chr6:58057646..58057646+	chr6	-	0,02415145	98449	NM_134172	V1rc17	L1Md_F	162
chr7:12329121..12329121+	chr7	-	0,09660578	73252	NM_145843	V1rg1	L1Md_F	616
chr7:13769334..13769334+	chr7	-	0,07524104	51626	NM_134227	V1rk1	L1Md_F	54
chr7:24742349..24742349+	chr7	+	0,02415145	26781	NM_207546	V1rd20	L1Md_F	1573
chr6:90042484..90042484+	chr6	+	0,09660578	14784	NM_053225	V1rb1	L1Md_F	-2946
chr7:5522072..5522072+	chr7	+	0,07245434	104851	NM_207542	V1rd8	L1Md_F	-71
chr13:22549702..22549702+	chr13	+	0,04830289	16784	NM_134221	V1rn4	L1Md_F2	390
chr17:21436847..21436847+	chr17	+	0,14490867	14116	NM_134200	V1rf3	L1Md_F2	491
chr17:21442709..21442709+	chr17	+	0,04830289	8271	NM_134200	V1rf3	L1Md_F2	987
chr6:66547900..66547900+	chr6	-	0,02415145	44115	NM_134170	V1rc15	L1Md_F2	6487
chr7:10919494..10919494+	chr7	+	0,07245434	112665	NM_134229	V1re10	L1Md_F2	1903
chr7:10922472..10922472+	chr7	+	0,16906012	109687	NM_134229	V1re10	L1Md_F2	4881
chr7:10922682..10922682+	chr7	+	0,02415145	109477	NM_134229	V1re10	L1Md_F2	5091
chr7:10923812..10923812+	chr7	+	0,02415145	108347	NM_134229	V1re10	L1Md_F2	368
chr7:12267757..12267757+	chr7	-	0,02415145	11888	NM_145843	V1rg1	L1Md_F2	612
chr7:12354022..12354022+	chr7	+	0,02415145	78101	NM_134206	V1rg5	L1Md_F2	1574
chr7:12357315..12357315+	chr7	+	0,02415145	74808	NM_134206	V1rg5	L1Md_F2	921
chr7:12552605..12552609+	chr7	-	0,09660578	35974	NM_134205	V1rg4	L1Md_F2	16
chr7:24665111..24665111+	chr7	-	0,07245434	13642	NM_206872	V1rd12	L1Md_F2	1002
chr7:24795616..24795616+	chr7	+	0,02415145	44176	NM_203489	V1rd15	L1Md_F2	158
chr6:57299015..57299015+	chr6	-	0,04830289	25385	NM_134184	V1rc29	L1Md_F2	-2569
chr7:5523977..5523977+	chr7	+	0,02415145	102946	NM_207542	V1rd8	L1Md_F2	-352
chr7:5547413..5547413+	chr7	+	0,02415145	79510	NM_207542	V1rd8	L1Md_F2	-3964
chr7:11483464..11483464+	chr7	-	0,02415145	149355	NM_145848	V1re13	L1Md_F3	1500
chr7:5898263..5898265+	chr7	-	0,2656659	62120	NM_207544	V1rd11	L1Md_F3	-29
chr17:20765364..20765378+	chr17	+	0,07245434	59107	NM_134191	V1re2	L1Md_Gf	40
chr13:22517741..22517741+	chr13	+	0,19321156	48745	NM_134221	V1rn4	L1Md_T	2899
chr6:57007251..57007251+	chr6	+	0,02415145	13684	NM_134185	V1rc30	L1Md_T	167
chr6:66825278..66825766+	chr6	-	219.636.491	98630	NM_134168	V1rc13	L1Md_T	1462
chr6:57144265..57144277+	chr6	+	0,04830289	15586	NM_053235	V1rc5	L1_Mm	411
chr6:90092431..90092431+	chr6	+	0,02415145	36278	NM_053222	V1ra7	L1_Mm	568
chr6:90092865..90092865+	chr6	+	0,21736301	35844	NM_053222	V1ra7	L1_Mm	1002
chr7:27432112..27432112+	chr7	-	0,21736301	35014	NM_134231	V1re12	L1_Mm	544
chr6:56917478..56917478+	chr6	+	0,04830289	17857	NM_134174	V1rc19	L1_Mm	-368
chr13:22522218..22522218+	chr13	+	0,02415145	44268	NM_134221	V1rn4	L1_Mur1	181
chr13:22754672..22754672+	chr13	-	0,02415145	41767	NM_134216	V1rh7	L1_Mur1	52
chr17:20932157..20932157+	chr17	-	0,09660578	17938	NM_134192	V1re3	L1_Mur1	421
chr17:21417867..21417867+	chr17	+	0,02415145	5718	NM_134201	V1rf4	L1_Mur1	302
chr6:66529715..66529715+	chr6	-	0,28961734	25930	NM_134170	V1rc15	L1_Mur1	975
chr6:66529920..66529920+	chr6	-	0,02415145	26135	NM_134170	V1rc15	L1_Mur1	770
chr7:10951896..10951896+	chr7	+	0,16906012	80263	NM_134229	V1re10	L1_Mur1	299
chr7:11023452..11023452+	chr7	-	0,02415145	162998	NM_134230	V1re11	L1_Mur1	1005
chr6:57119317..57119317+	chr6	+	0,12075723	40534	NM_053235	V1rc5	L1_Mur1	-151
chr6:57120568..57120568+	chr6	+	0,02415145	39283	NM_053235	V1rc5	L1_Mur1	-1433
chr13:22848940..22848940+	chr13	-	0,02415145	136035	NM_134216	V1rh7	L1_Mur1	318
chr17:20747181..20747181+	chr17	+	0,16906012	77290	NM_134191	V1re2	L1_Mur2	196
chr17:20752050..20752050+	chr17	+	0,02415145	72421	NM_134191	V1re2	L1_Mur2	1527
chr17:21208976..21208976+	chr17	+	0,07245434	156813	NM_134198	V1rf1	L1_Mur2	17
chr6:57073898..57073898+	chr6	+	0,14490867	13448	NM_053233	V1rc3	L1_Mur2	59
chr6:57073965..57073965+	chr6	+	0,07245434	13381	NM_053233	V1rc3	L1_Mur2	126
chr7:5560847..5560847+	chr7	+	0,07245434	66076	NM_207542	V1rd8	L1_Mur2	919
chr17:20805716..20805716+	chr17	+	0,02415145	18755	NM_134191	V1re2	L1_Mur2	-809
chr6:90120843..90120843+	chr6	+	0,24151445	7866	NM_053222	V1ra7	L1_Mur2	-1
chr13:22464263..22464263+	chr13	+	0,24151445	10143	NM_134213	V1rh4	L1_Mur3	2
chr13:22544133..22544133+	chr13	+	0,02415145	22353	NM_134221	V1rn4	L1_Mur3	345
chr13:22725359..22725359+	chr13	-	0,02415145	12454	NM_134216	V1rh7	L1_Mur3	195
chr13:22971667..22971667+	chr13	-	0,02415145	27302	NM_134243	V1rh20	L1_Mur3	487
chr13:23097015..23097015+	chr13	+	0,02415145	6102	NM_134215	V1rh6	L1_Mur3	35
chr13:23117397..23117397+	chr13	+	0,02415145	8809	NM_134214	V1rh5	L1_Mur3	351
chr6:58184881..58184881+	chr6	+	0,02415145	30286	NM_134180	V1rc25	L1_Mur3	201
chr7:12701493..12701493+	chr7	+	0,09660578	36319	NM_134208	V1rg7	L1_Mur3	170
chr7:12701668..12701668+	chr7	+	0,02415145	36144	NM_134208	V1rg7	L1_Mur3	345
chr7:5878980..5878980+	chr7	-	0,07245434	42835	NM_207544	V1rd11	L1_Mur3	1187
chr7:5879005..5879005+	chr7	-	0,19321156	42860	NM_207544	V1rd11	L1_Mur3	1162
chr13:23220272..23220272+	chr13	-	0,02415145	13672	NM_134239	V1rh14	L1_Mur3	-53
chr6:57005961..57005963+	chr6	+	0,09660578	14974	NM_134185	V1rc30	L1_Mur3	-51
chr6:57005988..57005988+	chr6	+	0,16906012	14947	NM_134185	V1rc30	L1_Mur3	-26
chr6:57314747..57314749+	chr6	-	0,24151445	3376	NM_134171	V1rc16	L1_Mur3	-77
chr7:11187950..11187950+	chr7	+	0,02415145	30985	NM_134228	V1rf1	L1_Mur3	-257
chr7:12909386..12909386+	chr7	-	0,02415145	1908	NM_134209	V1rg8	L1_Mur3	-60
chr13:22540645..22540645+	chr13	+	0,02415145	25841	NM_134221	V1rn4	L1_Mur3	574
chr6:58080181..58080181+	chr6	-	0,12075723	120984	NM_134172	V1rc17	L1_Mus1	301
chr6:66645662..66645662+	chr6	-	0,12075723	15983	NM_134167	V1rc12	L1_Mus1	63
chr6:56931763..56931771+	chr6	+	0,04830289	3572	NM_134174	V1rc19	L1_Mus1	-513
chr6:58200767..58200767+	chr6	+	0,02415145	14400	NM_134180	V1rc25	L1_Mus1	-3714
chr7:11023354..11023354+	chr7	-	0,02415145	162900	NM_134230	V1re11	L1_Mus1	-450
chr17:20863429..20863429+	chr17	+	0,02415145	8634	NM_134195	V1re6	L1_Mus2	347
chr17:21210593..21210593+	chr17	+	0,02415145	155196	NM_134198	V1rf1	L1_Mus2	1116
chr17:21215803..21215803+	chr17	+	0,09660578	149986	NM_134198	V1rf1	L1_Mus2	6326
chr6:90134720..90134720+	chr6	+	0,12075723	18090	NM_053223	V1ra8	L1_Mus2	635
chr7:5531778..5531778+	chr7	+	0,02415145	95145	NM_207542	V1rd8	L1_Mus2	151
chr7:24408094..24408094+	chr7	+	0,02415145	9276	NM_030737	V1rd7	L1_Mus2	-1182
chr7:5585958..5585958+	chr7	+	0,02415145	40965	NM_207542	V1rd8	L1_Mus2	-10
chr6:89892088..89892088+	chr6	-	0,02415145	8107	NM_011684	V1ra2	L1_Mus3	175
chr7:5374748..5374748+	chr7	-	0,09660578	11916	NM_030739	V1rd4	L1_Mus3	1540
chr7:24740281..24740292+	chr7	+	0,21736301	28849	NM_207546	V1rd20	L1_Mus4	829
chrX:143893926..143893944+	chrX	+	0,16906012	5672	NM_053240	V1rb10	L1_Mus4	35
chr13:22605241..22605241+	chr13	-	0,02415145	11126	NM_134224	V1r17	L1_Mus4	-1333
chr6:58237654..58237654+	chr6	+	0,04830289	19636	NM_053232	V1rc2	L1_Mus4	-4464
chr6:66519188..66519188+	chr6	-	0,31396879	15403	NM_134170	V1rc15	L1_Mus4	-429
chr7:27426138..27426138+	chr7	-	0,02415145	29040	NM_134231	V1re12	L1_Mus4	-626
chr6:66658361..66658361+	chr6	-	0,04830289	28682	NM_134167	V1rc12	L1_Rod	214
chr6:66787991..66787991+	chr6	-	0,02415145	60866	NM_134168	V1rc13	L1_Rod	197
chr6:66788183..66788183+	chr6	-	0,02415145	61058	NM_134168	V1rc13	L1_Rod	5
chr7:12261191..12261191+	chr7	+	0,19321156	80414	NM_134203	V1rg2	L1_Rod	525
chr7:12588053..12588053+	chr7	-	0,02415145	71418	NM_134205	V1rg4	L1_Rod	151
chr6:57034022..57034022+	chr6	+	0,02415145	29396	NM_053231	V1rc1	L1_Rod	-54
chr6:57082856..57082856+	chr6	+	0,16906012	4490	NM_053233	V1rc3	L1_Rod	-179
chr6:57807716..57807716+	chr6	+	0,04830289	13264	NM_134183	V1rc28	L1_Rod	-299
chr7:12912687..12912687+	chr7	-	0,12075723	5209	NM_134209	V1rg8	L1M3	-49
chr13:23220164..23220164+	chr13	+	0,02415145	8189	NM_134222	V1r15	L1MD	54
chr7:12998020..12998032+	chr7	+	0,38642312	49912	NM_134233	V1rg11	L1MD2	-181
chr7:12998068..12998068+	chr7	-	0,24151445	49954	NM_134233	V1rg11	L1MD2	-229
chr7:12998120..12998129+	chr7	-	0,21736301	50015	NM_134233	V1rg11	L1MD2	-281
chr6:89799686..89799686+	chr6	-	0,02415145	4154	NM_053221	V1ra6	L1VL1	83
chr7:10980874..10980874+	chr7	+	0,33812023	51285	NM_134229	V1re10	L1VL2	2064
chr7:12334233..12334235+	chr7	+	0,14490867	78366	NM_145843	V1rg1	L1VL2	388
chr6:57983661..57983661+	chr6	-	0,02415145	24464	NM_134172	V1rc17	L1VL2	-857
chr17:20812118..20812118+	chr17	+	0,02415145	12353	NM_134191	V1re2	L1VL4	252
chr6:57953972..57953972+	chr6	-	0,02415145	24675	NM_053238	V1rc8	L1VL4	661
chr6:89804508..89804508+	chr6	-	0,02415145	8976	NM_053221	V1ra6	L1VL4	1383
chr6:89805482..89805482+	chr6	-	0,04830289	9950	NM_053221	V1ra6	L1VL4	409
chr6:89901675..89901675+	chr6	-	0,02415145	17694	NM_011684	V1ra2	L1VL4	1649
chr6:89902653..89902653								

chr7:12363755..12363762+	chr7	+	0,03622717	68361	NM_134206	V1rg5	Lx	-377
chr7:12424426..12424426+	chr7	+	0,16906012	7697	NM_134206	V1rg5	Lx	-4842
chr7:12550645..12550645+	chr7	+	0,02415145	187167	NM_134208	V1rg7	Lx	-169
chr7:24305362..24305362+	chr7	+	0,02415145	112008	NM_030737	V1rd7	Lx	-553
chr17:20629977..20629977+	chr17	+	0,02415145	9285	NM_134194	V1re5	Lx2	558
chr17:20632956..20632956+	chr17	+	0,12075723	6306	NM_134194	V1re5	Lx2	525
chr17:20815667..20815667+	chr17	+	0,16906012	8804	NM_134191	V1re2	Lx2	2071
chr17:20816770..20816770+	chr17	+	0,02415145	7701	NM_134191	V1re2	Lx2	3174
chr17:20925110..20925112-	chr17	-	0,16906012	10893	NM_134192	V1re3	Lx2	615
chr6:89827242..89827242-	chr6	-	0,07245434	6793	NM_053220	V1ra5	Lx2	566
chr7:12349008..12349008+	chr7	+	0,02415145	83115	NM_134206	V1rg5	Lx2	132
chr7:12349543..12349543+	chr7	+	0,21736301	82580	NM_134206	V1rg5	Lx2	667
chr7:12349918..12349918+	chr7	+	0,07245434	82205	NM_134206	V1rg5	Lx2	1042
chr7:24674310..24674310-	chr7	-	0,02415145	22841	NM_206872	V1rd12	Lx2	169
chr7:5236800..5236800-	chr7	-	0,04830289	88581	NM_030740	V1rd3	Lx2	459
chrX:143900521..143900521-	chrX	-	0,04830289	12267	NM_053240	V1rb10	Lx2	1907
chr13:22486359..22486359+	chr13	+	0,02415145	538	NM_134212	V1rh3	Lx2	-291
chr6:57879113..57879113-	chr6	-	0,09660578	2327	NM_134179	V1rc24	Lx2	-778
chr7:13791872..13791874+	chr7	+	0,04830289	12815	NM_134226	V1rj2	Lx2A1	2015
chr13:22414387..22414387+	chr13	+	0,02415145	5392	NM_134244	V1rh21	Lx2B	273
chr7:12315245..12315245-	chr7	-	0,16906012	59376	NM_145843	V1rg1	Lx2B	67
chr7:24793053..24793054+	chr7	+	0,21736301	46739	NM_203489	V1rd15	Lx2B	508
chr17:20801671..20801671+	chr17	+	0,02415145	22800	NM_134191	V1re2	Lx3_Mus	219
chr17:20801780..20801780+	chr17	+	0,07245434	22691	NM_134191	V1re2	Lx3_Mus	328
chr6:58453127..58453127-	chr6	-	0,09660578	67287	NM_134177	V1rc22	Lx3_Mus	688
chr7:5798359..5798359-	chr7	-	0,04830289	43125	NM_030742	V1rd1	Lx3_Mus	342
chr6:57915129..57915129-	chr6	-	0,02415145	8603	NM_134173	V1rc18	Lx3_Mus	-2990
chr7:11018331..11018331-	chr7	-	0,12075723	157877	NM_134230	V1re11	Lx3A	997
chr7:12350751..12350751+	chr7	+	0,09660578	81372	NM_134206	V1rg5	Lx3A	26
chr6:56879409..56879412+	chr6	+	0,36227168	27097	NM_134176	V1rc21	Lx3A	-6
chr6:57817733..57817733-	chr6	-	0,00966058	23281	NM_134183	V1rc28	Lx3A	-6407
chr6:57817793..57817793-	chr6	-	0,04830289	23341	NM_134183	V1rc28	Lx3A	-6467
chr6:66626716..66626716-	chr6	-	0,02415145	64153	NM_134169	V1rc14	Lx3A	-75
chr13:23086210..23086210+	chr13	+	0,02415145	16907	NM_134215	V1rh6	Lx3B	1179
chr17:21139951..21139951-	chr17	-	0,07245434	8300	NM_134202	V1rf5	Lx3B	-1642
chr17:21140158..21140158-	chr17	-	0,21736301	8507	NM_134202	V1rf5	Lx3B	-1849
chr6:58159923..58159923-	chr6	-	0,02415145	200726	NM_134172	V1rc17	Lx3B	-2268
chr7:13695224..13695224-	chr7	-	0,02415145	24659	NM_145847	V1rj3	Lx3B	-2066
chr7:24772164..24772164+	chr7	+	0,02415145	15965	NM_207619	V1rd19	Lx3C	1048
chr7:5859674..5859674-	chr7	-	0,02415145	23529	NM_207544	V1rd11	Lx3C	357
chr17:21411363..21411363+	chr17	+	0,02415145	12222	NM_134201	V1rf4	Lx4A	373
chr7:12297017..12297017+	chr7	+	0,02415145	44588	NM_134203	V1rg2	Lx4A	-67
chr6:57163575..57163575+	chr6	+	0,07245434	19857	NM_053237	V1rc7	Lx4A	786
chr6:57167343..57167343+	chr6	+	0,02415145	16089	NM_053237	V1rc7	Lx4B	3595
chr6:58183834..58183834+	chr6	+	0,14490867	31333	NM_134180	V1rc25	Lx4B	585
chr13:22709074..22709074-	chr13	-	0,12075723	24274	NM_134217	V1rh8	Lx4B	-265
chr7:24323410..24323410+	chr7	+	0,02415145	93960	NM_030737	V1rd7	Lx4B	-453
chr7:24653658..24653658-	chr7	-	0,02415145	2189	NM_206872	V1rd12	Lx4B	-291
chr13:22348046..22348046+	chr13	+	0,02415145	22184	NM_134223	V1rf6	Lx5	393
chr7:12267483..12267483-	chr7	-	0,02415145	11614	NM_145843	V1rg1	Lx5	260
chr7:12269013..12269023-	chr7	-	0,16906012	13154	NM_145843	V1rg1	Lx5	883
chr7:12309655..12309655-	chr7	-	0,02415145	53786	NM_145843	V1rg1	Lx5	116
chr7:12703848..12703848-	chr7	-	0,02415145	187213	NM_134205	V1rg4	Lx5	1213
chr13:22426815..22426815+	chr13	+	0,02415145	19399	NM_134220	V1rj3	Lx5	-982
chr17:20833838..20833838+	chr17	+	0,16906012	38225	NM_134195	V1re6	Lx5	-742
chr17:20835368..20835368+	chr17	+	0,02415145	36695	NM_134195	V1re6	Lx5	-1546
chr6:57821017..57821017-	chr6	-	0,02415145	26565	NM_134183	V1rc28	Lx5	-804
chr7:12311169..12311169+	chr7	+	0,04830289	30436	NM_134203	V1rg2	Lx5	-30
chr13:22688330..22688330-	chr13	-	0,02415145	3530	NM_134217	V1rh8	Lx6	955
chr7:10865341..10865341+	chr7	+	0,02415145	166818	NM_134229	V1re10	Lx6	274
chr7:24440598..24440598+	chr7	+	0,16906012	4112	NM_030735	V1rd9	Lx6	-96
chr13:22600447..22600447-	chr13	-	0,38642312	6332	NM_134224	V1rj7	Lx7	63
chr6:57335350..57335350-	chr6	-	0,04830289	23977	NM_134171	V1rc16	Lx7	-618
chr6:58226200..58226200+	chr6	+	0,02415145	31090	NM_053232	V1rc2	Lx7	-36
chr6:58403672..58403675-	chr6	-	0,14490867	17835	NM_134177	V1rc22	Lx7	-703
chr6:66547229..66547230-	chr6	-	0,21736301	43445	NM_134170	V1rc15	Lx7	-998
chr6:66556788..66556789-	chr6	-	0,07245434	53003	NM_134170	V1rc15	Lx7	-1400
chr6:66836598..66836598-	chr6	-	0,07245434	109473	NM_134168	V1rc13	Lx7	-123
chr7:12339627..12339627+	chr7	+	0,04830289	1978	NM_134203	V1rg2	Lx7	-150
chr7:24446183..24446183+	chr7	+	0,02415145	92746	NM_207548	V1rd22	Lx7	-1458
chr7:24764938..24764938+	chr7	+	0,02415145	4192	NM_207546	V1rd20	Lx7	-174
chr17:21048867..21048867-	chr17	-	0,14490867	21251	NM_134196	V1re7	Lx8	12
chr7:10978173..10978173+	chr7	+	0,02415145	53986	NM_134229	V1re10	Lx8	174
chr7:13786896..13786896-	chr7	-	0,02415145	69188	NM_134227	V1rk1	Lx8	114
chr17:21141927..21141927-	chr17	-	0,04830289	10276	NM_134202	V1rf5	Lx8	-531
chr7:11028036..11028036-	chr7	-	0,12075723	167582	NM_134230	V1re11	Lx8	-126
chr7:12631960..12631960-	chr7	-	0,04830289	115325	NM_134205	V1rg4	Lx8	-120
chr17:20775173..20775173+	chr17	+	0,07245434	49298	NM_134191	V1re2	Lx9	8
chr17:20860359..20860359+	chr17	+	0,04830289	11704	NM_134195	V1re6	Lx9	373
chr17:20949479..20949479-	chr17	-	0,02415145	35260	NM_134192	V1re3	Lx9	176
chr6:56984969..56984969+	chr6	+	0,02415145	990	NM_134187	V1rc32	Lx9	191
chr6:57930327..57930328-	chr6	-	0,24151445	1031	NM_053238	V1rc8	Lx9	120
chr6:58029149..58029149-	chr6	-	0,02415145	69952	NM_134172	V1rc17	Lx9	116
chr6:58256808..58256808+	chr6	+	0,07245434	482	NM_053232	V1rc2	Lx9	19
chr6:58256901..58256901+	chr6	+	0,02415145	389	NM_053232	V1rc2	Lx9	112
chr7:13770834..13770834-	chr7	-	0,12075723	53126	NM_134227	V1rk1	Lx9	273
chr6:58502823..58502823-	chr6	-	0,02415145	116983	NM_134177	V1rc22	Lx9	-22
chr6:58502876..58502876-	chr6	-	0,12075723	117036	NM_134177	V1rc22	Lx9	-75
chr17:20813519..20813519+	chr17	+	0,02415145	10952	NM_134191	V1re2	MusHAL1	133
chr6:57945771..57945771-	chr6	-	0,02415145	16474	NM_053238	V1rc8	MusHAL1	1070
chr17:20753870..20753870+	chr17	+	0,09660578	70601	NM_134191	V1re2	MusHAL1	-128
chr17:20956947..20956949+	chr17	+	0,07245434	26567	NM_134197	V1re8	MusHAL1	-191
chr7:5582111..5582111+	chr7	+	0,02415145	44812	NM_207542	V1rd8	MusHAL1	-201

Expressed LINE1s classification - V2Rs loci

Genomic Coordinates	Chromosome	strand	TPM	Distance to annotated TSS	RefseqID of associated VR	Gene symbol	Repeat Class/family	annotation.rmsk.tss_distance
chr6:123878826..123878826+	chr6	+	0,024151445	95949	NM_019917	Vmn2r26	L1Md_A	426
chr7:5468721..5468721-	chr7	-	0,144908671	22767	NM_001081405	Vmn2r28	L1Md_A	69
chr7:92208044..92208044-	chr7	-	0,024151445	47013	NM_001033878	Vmn2r66	L1Md_A	1233
chr3:64624477..64624477-	chr3	-	0,024151445	100961	NM_175674	Vmn2r7	L1Md_A	-766
chr7:7714010..7714010-	chr7	-	0,024151445	71400	NM_001105066	Vmn2r34	L1Md_A	-121
chr5:109636454..109636454-	chr5	-	0,096605781	14827	NM_001104624	Vmn2r13	L1Md_F	169
chr5:109636546..109636554-	chr5	-	0,04830289	14919	NM_001104624	Vmn2r13	L1Md_F	69
chr5:109735440..109735440-	chr5	-	0,024151445	8364	NM_001104626	Vmn2r15	L1Md_F	2000
chr7:5590183..5590183-	chr7	-	0,144908671	144229	NM_001081405	Vmn2r28	L1Md_F	801
chr7:7205630..7205630-	chr7	-	0,024151445	5090	NM_001113468	Vmn2r29	L1Md_F	537
chrX:121296764..121296764-	chrX	-	0,024151445	47323	NM_001100616	Vmn2r121	L1Md_F	278
chr5:109286287..109286287-	chr5	-	0,024151445	4257	NM_001104621	Vmn2r9	L1Md_F	-704
chr5:109340508..109340508-	chr5	-	0,024151445	58478	NM_001104621	Vmn2r9	L1Md_F	-72
chr10:78904191..78904191+	chr10	+	0,024151445	27511	NM_001104537	Vmn2r83	L1Md_F2	924
chr17:18174371..18174371+	chr17	+	0,024151445	47649	NM_001104540	Vmn2r91	L1Md_F2	721
chr17:18174450..18174450+	chr17	+	0,096605781	47570	NM_001104540	Vmn2r91	L1Md_F2	800
chr17:18668869..18668869+	chr17	+	0,096605781	50500	NM_001104547	Vmn2r96	L1Md_F2	210
chr17:57905147..57905147-	chr17	-	0,024151445	219909	NM_001104591	Vmn2r120	L1Md_F2	15
chr5:109360180..109360180-	chr5	-	0,120757226	78150	NM_001104621	Vmn2r9	L1Md_F2	3983
chr5:152441379..152441379-	chr5	-	0,144908671	51897	NM_001102582	Vmn2r18	L1Md_F2	1186
chr6:123820971..123820971+	chr6	+	0,024151445	153804	NM_019917	Vmn2r26	L1Md_F2	2378
chr7:10583545..10583545-	chr7	-	0,024151445	5556	NM_001105156	Vmn2r49	L1Md_F2	2561
chr7:49161085..49161085-	chr7	-	0,024151445	32544	NM_001105055	Vmn2r58	L1Md_F2	1870
chr7:5487761..5487761-	chr7	-	0,217363007	41807	NM_001081405	Vmn2r28	L1Md_F2	734
chr7:93938348..93938348+	chr7	+	0,072454336	5302	NM_001105188	Vmn2r77	L1Md_F2	272
chr7:93975943..93975943+	chr7	+	0,024151445	87915	NM_001105189	Vmn2r78	L1Md_F2	403
chr7:94057110..94057110+	chr7	+	0,265665897	6748	NM_001105189	Vmn2r78	L1Md_F2	138
chr7:94058536..94058537+	chr7	+	0,072454336	5322	NM_001105189	Vmn2r78	L1Md_F2	1564
chr17:19231807..19231807+	chr17	+	0,024151445	283785	NM_001104551	Vmn2r99	L1Md_F2	-195
chr17:20477154..20477154+	chr17	+	0,04830289	54259	NM_001104568	Vmn2r106	L1Md_F2	-603
chr17:57852942..57852942-	chr17	-	0,024151445	167704	NM_001104591	Vmn2r120	L1Md_F2	-61
chr7:92983018..92983018-	chr7	-	0,024151445	79026	NM_001105185	Vmn2r72	L1Md_F2	-732
chr17:19513856..19513856+	chr17	+	0,024151445	1736	NM_001104551	Vmn2r99	L1Md_F3	184
chr17:23501990..23501990+	chr17	+	0,024151445	19779	NM_001104580	Vmn2r116	L1Md_F3	1808
chr17:57704868..57704868-	chr17	-	0,024151445	19630	NM_001104591	Vmn2r120	L1Md_F3	33
chr5:109742028..109742028+	chr5	+	0,024151445	17371	NM_001104627	Vmn2r16	L1Md_F3	96
chr5:109742100..109742100+	chr5	+	0,024151445	17299	NM_001104627	Vmn2r16	L1Md_F3	168
chr7:10699621..10699621-	chr7	-	0,313968788	8112	NM_001105179	Vmn2r51	L1Md_F3	787
chr7:10699652..10699652-	chr7	-	0,024151445	8143	NM_001105179	Vmn2r51	L1Md_F3	756
chr7:92179230..92179230-	chr7	-	0,120757226	18199	NM_001033878	Vmn2r66	L1Md_F3	1541
chr7:93216259..93216259-	chr7	-	0,072454336	105766	NM_001105187	Vmn2r74	L1Md_F3	4182
chr7:50097699..50097699-	chr7	-	0,024151445	48332	NM_001105059	Vmn2r62	L1Md_T	1989
chr17:18623997..18623997+	chr17	+	0,120757226	95372	NM_001104547	Vmn2r96	L1Md_T	-9
chr17:18419246..18419246-	chr17	-	0,193211562	4773	NM_001104543	Vmn2r94	L1_Mm	657
chr17:20463360..20463360-	chr17	-	0,169060117	40465	NM_001104568	Vmn2r106	L1_Mm	1004
chr5:109348132..109348132-	chr5	-	0,144908671	66102	NM_001104621	Vmn2r9	L1_Mm	1672
chr7:7215150..7215150-	chr7	-	0,096605781	14610	NM_001113468	Vmn2r29	L1_Mm	507
chr7:7318223..7318223-	chr7	-	0,024151445	27518	NM_009490	Vmn2r30	L1_Mm	442
chr7:92224701..92224701-	chr7	-	0,144908671	63670	NM_001033878	Vmn2r66	L1_Mm	234
chr7:92698006..92698006-	chr7	-	0,024151445	133319	NM_001105182	Vmn2r69	L1_Mm	619
chr7:92365837..92365837-	chr7	-	0,024151445	60924	NM_001102579	Vmn2r67	L1_Mm	-20
chr7:93352605..93352605-	chr7	-	0,024151445	31870	NM_001102578	Vmn2r75	L1_Mm	-632
chr17:19602470..19602470+	chr17	+	0,217363007	39304	NM_001104562	Vmn2r100	L1_Mur1	63
chr17:19602715..19602715+	chr17	+	0,144908671	39059	NM_001104562	Vmn2r100	L1_Mur1	308
chr17:23619064..23619064+	chr17	+	0,217363007	1999	NM_001104581	Vmn2r117	L1_Mur1	921
chr7:49145380..49145380-	chr7	-	0,144908671	16839	NM_001105055	Vmn2r58	L1_Mur1	331
chr7:92319762..92319762-	chr7	-	0,024151445	14849	NM_001102579	Vmn2r67	L1_Mur1	2187
chr7:92396474..92396474-	chr7	-	0,169060117	9759	NM_001105181	Vmn2r68	L1_Mur1	795
chr7:92576261..92576261-	chr7	-	0,144908671	11574	NM_001105182	Vmn2r69	L1_Mur1	1250
chr7:92577351..92577351-	chr7	-	0,144908671	12664	NM_001105182	Vmn2r69	L1_Mur1	160
chr7:92577760..92577760-	chr7	-	0,024151445	13073	NM_001105182	Vmn2r69	L1_Mur1	19
chr7:92722540..92722540-	chr7	-	0,024151445	4441	NM_001105183	Vmn2r70	L1_Mur1	738
chr7:93113710..93113710-	chr7	-	0,096605781	3217	NM_001105187	Vmn2r74	L1_Mur1	724
chr7:93114260..93114260-	chr7	-	0,024151445	3767	NM_001105187	Vmn2r74	L1_Mur1	174
chr7:93325392..93325392-	chr7	-	0,024151445	4657	NM_001102578	Vmn2r75	L1_Mur1	746
chr5:152436153..152436153-	chr5	-	0,024151445	46671	NM_001102582	Vmn2r18	L1_Mur1	-900
chr7:92663322..92663323-	chr7	-	0,289817343	98636	NM_001105182	Vmn2r69	L1_Mur1	-30
chr17:18149038..18149041+	chr17	+	111.096.648	72982	NM_001104540	Vmn2r91	L1_Mur2	158
chr17:18206855..18206855+	chr17	+	0,024151445	15165	NM_001104540	Vmn2r91	L1_Mur2	253
chr17:18607156..18607156+	chr17	+	0,217363007	112213	NM_001104547	Vmn2r96	L1_Mur2	909
chr17:18607498..18607498+	chr17	+	0,024151445	111871	NM_001104547	Vmn2r96	L1_Mur2	1251
chr17:18607827..18607827+	chr17	+	0,024151445	111542	NM_001104547	Vmn2r96	L1_Mur2	1580
chr17:18612097..18612097+	chr17	+	0,169060117	107272	NM_001104547	Vmn2r96	L1_Mur2	267
chr17:18645828..18645828+	chr17	+	0,024151445	73541	NM_001104547	Vmn2r96	L1_Mur2	47
chr17:19237681..19237681+	chr17	+	0,096605781	277911	NM_001104551	Vmn2r99	L1_Mur2	530
chr17:19237904..19237904+	chr17	+	0,024151445	277688	NM_001104551	Vmn2r99	L1_Mur2	753
chr17:19237947..19237947+	chr17	+	0,024151445	277645	NM_001104551	Vmn2r99	L1_Mur2	4
chr17:19249732..19249744+	chr17	+	0,04830289	265860	NM_001104551	Vmn2r99	L1_Mur2	103
chr17:19322690..19322690+	chr17	+	0,024151445	129202	NM_001104551	Vmn2r99	L1_Mur2	377
chr17:19323450..19323450+	chr17	+	0,024151445	192142	NM_001104551	Vmn2r99	L1_Mur2	1137
chr17:19323473..19323473+	chr17	+	0,024151445	192119	NM_001104551	Vmn2r99	L1_Mur2	1160
chr17:19384923..19384923+	chr17	+	0,072454336	130669	NM_001104551	Vmn2r99	L1_Mur2	475
chr17:19554429..19554429+	chr17	+	0,144908671	87345	NM_001104562	Vmn2r100	L1_Mur2	471
chr17:19554522..19554522+	chr17	+	0,024151445	87252	NM_001104562	Vmn2r100	L1_Mur2	564
chr17:19559794..19559794+	chr17	+	0,120757226	81980	NM_001104562	Vmn2r100	L1_Mur2	41
chr17:19563231..19563231+	chr17	+	0,024151445	78543	NM_001104562	Vmn2r100	L1_Mur2	589
chr17:19850436..19850436+	chr17	+	0,024151445	59890	NM_001104565	Vmn2r103	L1_Mur2	304
chr17:19851389..19851389+	chr17	+	0,096605781	58937	NM_001104565	Vmn2r103	L1_Mur2	581
chr17:19889400..19889400+	chr17	+	0,024151445	20926	NM_001104565	Vmn2r103	L1_Mur2	345
chr17:19889706..19889706+	chr17	+	0,024151445	20620	NM_001104565	Vmn2r103	L1_Mur2	35
chr17:20301796..20301796-	chr17	-	0,04830289	116126	NM_001104566	Vmn2r104	L1_Mur2	20
chr17:20451159..20451159+	chr17	+	0,096605781	28264	NM_001104568	Vmn2r106	L1_Mur2	145
chr17:22731306..22731306+	chr17	+	0,144908671	6808	NM_001104575	Vmn2r112	L1_Mur2	414
chr3:64364420..64364420-	chr3	-	0,096605781	688	NM_001104619	Vmn2r6	L1_Mur2	212
chr3:64364627..64364627-	chr3	-	0,024151445	895	NM_001104619	Vmn2r6	L1_Mur2	5
chr5:152423217..152423217-	chr5	-	0,120757226	33735	NM_001102582	Vmn2r18	L1_Mur2	1085
chr6:123877490..123877490-	chr6	-	0,024151445	73781	NM_001104641	Vmn2r25	L1_Mur2	21
chr7:10845126..10845126-	chr7	-	0,072454336	82990	NM_001105191	Vmn2r52	L1_Mur2	149
chr7:49262638..49262638-	chr7	-	0,024151445	134097	NM_001105055	Vmn2r58	L1_Mur2	329
chr7:8890147..8890150+	chr7	+	0,030189307	64224	NM_009489	Vmn2r37	L1_Mur2	97
chr7:92411214..92411214+	chr7	+	0,144908671	24499	NM_001105181	Vmn2r68	L1_Mur2	500
chr7:92724503..92724503-	chr7	-	0,024151445	6404	NM_001105183	Vmn2r70	L1_Mur2	777
chr7:94115885..94115885+	chr7	+	0,096605781	29089	NM_001105190	Vmn2r79	L1_Mur2	115
chr17:55790766..55790766-	chr17	-	0,072454336	26295	NM_001104582	Vmn2r118	L1_Mur2	-17
chr17:55790799..55790807-	chr17	-	0,04830289	26328	NM_001104582	Vmn2r118	L1_Mur2	-50
chr3:64555384..64555384+	chr3	+	0,024151445	31868	NM_175674	Vmn2r7	L1_Mur2	-1036
chr3:64555867..64555867-	chr3	-	0,024151445	32351	NM_175674	Vmn2r7	L1_Mur2	-16
chr6:123929472..123929472-	chr6	-	0,096605781	125763	NM_001104641	Vmn2r25	L1_Mur2	-393
chr7:92273673..92273673-	chr7	-	0,024151445	112642	NM_001033878	Vmn2r66	L1_Mur2	-22
chr7:92274874..92274874+	chr7	+	0,024151445	39097	NM_001105184	Vmn2r71	L1_Mur2	-406
chr7:94128447..94128447+	chr7	+	0,120757226	16527				

chr6:123955013..123955013-	chr6	-	0,04830289	151304	NM_001104641	Vmn2r25	L1_Mur3	67
chr3:64695607..64695607-	chr3	-	0,024151445	172091	NM_175674	Vmn2r7	L1_Mur3	-147
chr7:48730432..48730434-	chr7	-	0,410574569	25922	NM_177764	Vmn2r57	L1_Mur3	-79
chr7:48731792..48731793-	chr7	-	0,748694802	27281	NM_177764	Vmn2r57	L1_Mur3	-91
chr7:92757153..92757153+	chr7	+	0,024151445	6818	NM_001105184	Vmn2r71	L1_Mur3	-188
chr17:18282905..18282905+	chr17	+	0,072454336	5988	NM_001104541	Vmn2r92	L1_Mus1	1138
chr17:18662310..18662310+	chr17	+	0,04830289	57059	NM_001104547	Vmn2r96	L1_Mus1	136
chr17:19495617..19495617+	chr17	+	0,265665897	19975	NM_001104551	Vmn2r99	L1_Mus1	378
chr7:10732261..10732261-	chr7	-	0,096605781	40752	NM_001105179	Vmn2r51	L1_Mus1	649
chr7:92477263..92477263-	chr7	-	0,096605781	90548	NM_001105181	Vmn2r68	L1_Mus1	1006
chr7:92927657..92927657-	chr7	-	0,072454336	23665	NM_001105185	Vmn2r72	L1_Mus1	216
chr7:92929025..92929025-	chr7	-	0,04830289	25033	NM_001105185	Vmn2r72	L1_Mus1	1036
chr10:78678378..78678378+	chr10	+	0,072454336	32160	NM_175936	Vmn2r81	L1_Mus1	-632
chr10:78701929..78701929+	chr10	+	0,144908671	8609	NM_175936	Vmn2r81	L1_Mus1	-3973
chr10:78703612..78703612+	chr10	+	0,193211562	6926	NM_175936	Vmn2r81	L1_Mus1	-2290
chr7:49250216..49250217-	chr7	-	0,07513783	121676	NM_001105055	Vmn2r58	L1_Mus1	-865
chr17:17825936..17825936+	chr17	+	0,024151445	14968	NM_001104539	Vmn2r90	L1_Mus2	513
chr17:20296801..20296801-	chr17	-	0,04830289	111131	NM_001104566	Vmn2r104	L1_Mus2	1166
chr17:20297645..20297645-	chr17	-	0,024151445	111975	NM_001104566	Vmn2r104	L1_Mus2	322
chr17:20311645..20311645-	chr17	-	0,024151445	125975	NM_001104566	Vmn2r104	L1_Mus2	1152
chr17:55781524..55781524-	chr17	-	0,024151445	17053	NM_001104582	Vmn2r118	L1_Mus2	1701
chr5:109257492..109257492-	chr5	-	0,024151445	19218	NM_001104620	Vmn2r8	L1_Mus2	438
chr6:123891109..123891109+	chr6	+	0,024151445	83666	NM_019917	Vmn2r26	L1_Mus2	438
chr7:92534726..92534726-	chr7	-	0,072454336	148011	NM_001105181	Vmn2r68	L1_Mus2	21
chr7:92687692..92687692-	chr7	-	0,024151445	123005	NM_001105182	Vmn2r69	L1_Mus2	346
chr17:18795465..18795465+	chr17	+	0,024151445	255820	NM_001104549	Vmn2r97	L1_Mus2	-1083
chr17:18797353..18797353+	chr17	+	0,024151445	253932	NM_001104549	Vmn2r97	L1_Mus2	-614
chr6:123895080..123895080-	chr6	-	0,024151445	91371	NM_001104641	Vmn2r25	L1_Mus2	-2198
chr17:57722855..57722855-	chr17	-	0,169060117	37617	NM_001104591	Vmn2r120	L1_Mus3	213
chr17:57723554..57723554-	chr17	-	0,072454336	38316	NM_001104591	Vmn2r120	L1_Mus3	627
chr6:123856303..123856303-	chr6	-	0,120757226	52594	NM_001104641	Vmn2r25	L1_Mus3	323
chr6:123942973..123942973+	chr6	+	0,169060117	31802	NM_019917	Vmn2r26	L1_Mus3	259
chr17:18173426..18173426+	chr17	+	0,024151445	48594	NM_001104540	Vmn2r91	L1_Mus3	-225
chr17:19703464..19703464+	chr17	+	0,024151445	10730	NM_001104563	Vmn2r101	L1_Mus3	-1294
chr17:55772895..55772897-	chr17	-	0,060378613	8424	NM_001104582	Vmn2r118	L1_Mus3	-2789
chr3:64644641..64644641-	chr3	-	0,024151445	121125	NM_175674	Vmn2r7	L1_Mus3	-473
chr5:152417552..152417553-	chr5	-	0,144908671	28071	NM_001102582	Vmn2r18	L1_Mus4	5067
chr7:50199956..50199959-	chr7	-	0,169060117	10299	NM_001105060	Vmn2r63	L1_Mus4	333
chr17:57737849..57737849-	chr17	-	0,024151445	52611	NM_001104591	Vmn2r120	L1_Mus4	-477
chr7:93093669..93093669-	chr7	-	0,024151445	68720	NM_001105186	Vmn2r73	L1_Mus4	-739
chr17:20704275..20704275-	chr17	-	0,024151445	2054	NM_001104571	Vmn2r109	L1M2	76
chr5:109569195..109569195-	chr5	-	0,024151445	41811	NM_001104623	Vmn2r12	L1M3	88
chrX:121340562..121340562-	chrX	-	0,024151445	91121	NM_001100616	Vmn2r121	L1M5	144
chr7:13385985..13385985-	chr7	-	0,024151445	67030	NM_001104648	Vmn2r56	L1M4Ea	70
chr17:18514946..18514946+	chr17	+	0,024151445	46121	NM_001102581	Vmn2r95	L1V12	1488
chr17:20478869..20478869+	chr17	+	0,072454336	3519	NM_001104569	Vmn2r107	L1V12	228
chr17:20479865..20479865+	chr17	+	0,024151445	2523	NM_001104569	Vmn2r107	L1V12	1224
chr7:92531030..92531030-	chr7	-	0,024151445	144315	NM_001105181	Vmn2r68	L1V12	593
chr7:93353869..93353869-	chr7	-	0,024151445	33134	NM_001102578	Vmn2r75	L1V12	-14
chr10:78921539..78921539+	chr10	+	0,120757226	10163	NM_001104537	Vmn2r83	L1V14	704
chr17:18122274..18122274+	chr17	+	0,096605781	99746	NM_001104540	Vmn2r91	L1V14	1076
chr17:18426302..18426302+	chr17	+	0,096605781	8942	NM_001104542	Vmn2r93	L1V14	413
chr17:19861587..19861587+	chr17	+	0,169060117	48739	NM_001104565	Vmn2r103	L1V14	299
chr6:123913584..123913584-	chr6	-	0,024151445	109875	NM_001104641	Vmn2r25	L1V14	832
chr6:123913840..123913840-	chr6	-	0,036227168	110131	NM_001104641	Vmn2r25	L1V14	576
chr7:50201312..50201312-	chr7	-	0,096605781	11652	NM_001105060	Vmn2r63	L1V14	695
chr7:92193851..92193851-	chr7	-	0,024151445	32820	NM_001033878	Vmn2r66	L1V14	115
chr10:78604776..78604776+	chr10	+	0,024151445	6784	NM_001103368	Vmn2r80	L1V14	-4275
chr10:78609029..78609029+	chr10	+	0,024151445	2531	NM_001103368	Vmn2r80	L1V14	-22
chr5:109655591..109655591-	chr5	-	0,072454336	1449	NM_001104625	Vmn2r14	L1V14	-832
chr6:124199132..124199132-	chr6	-	0,04830289	16829	NM_001104642	Vmn2r27	L1V14	-560
chr3:64812938..64812938-	chr3	-	0,024151445	289422	NM_175674	Vmn2r7	L2	56
chr3:64794464..64794464-	chr3	-	0,072454336	270948	NM_175674	Vmn2r7	L2a	208
chr17:57849985..57849985-	chr17	-	0,024151445	164747	NM_001104591	Vmn2r120	Lx	927
chr6:123617815..123617815+	chr6	+	0,024151445	35023	NM_001104638	Vmn2r23	Lx	2241
chr7:49219168..49219168-	chr7	-	0,144908671	90627	NM_001105055	Vmn2r58	Lx	264
chr7:49369864..49369864-	chr7	-	0,024151445	55012	NM_001105056	Vmn2r59	Lx	179
chr7:92318472..92318472-	chr7	-	0,024151445	13559	NM_001102579	Vmn2r67	Lx	306
chr17:20442823..20442823-	chr17	-	0,096605781	19928	NM_001104568	Vmn2r106	Lx	-461
chr3:64591824..64591824-	chr3	-	0,024151445	68308	NM_175674	Vmn2r7	Lx	-616
chr6:123956134..123956134+	chr6	+	0,096605781	18641	NM_019917	Vmn2r26	Lx	-2143
chr7:49342948..49342948+	chr7	+	0,024151445	28892	NM_001105057	Vmn2r60	Lx	-9
chr7:49369770..49369770+	chr7	+	0,024151445	2070	NM_001105057	Vmn2r60	Lx	-273
chr7:92955895..92955895-	chr7	-	0,024151445	51903	NM_001105185	Vmn2r72	Lx	-410
chr7:93227809..93227809-	chr7	-	0,024151445	117316	NM_001105187	Vmn2r74	Lx	-621
chr17:18390896..18390896+	chr17	+	0,072454336	1124696	NM_001104551	Vmn2r99	Lx2	433
chr17:20749544..20749544-	chr17	-	0,024151445	15820	NM_001104572	Vmn2r110	Lx2	169
chr7:92743479..92743480+	chr7	+	0,193211562	20492	NM_001105184	Vmn2r71	Lx2	1336
chr17:18151878..18151878+	chr17	+	0,024151445	70142	NM_001104540	Vmn2r91	Lx2	-253
chr17:18655411..18655411+	chr17	+	0,024151445	63958	NM_001104547	Vmn2r96	Lx2	-145
chr17:18963717..18963717+	chr17	+	0,120757226	87568	NM_001104549	Vmn2r97	Lx2	-254
chr17:20310467..20310467-	chr17	-	0,024151445	124797	NM_001104566	Vmn2r104	Lx2	-761
chr7:49452473..49452473+	chr7	+	0,024151445	62949	NM_001105058	Vmn2r61	Lx2	-912
chr7:92423548..92423548-	chr7	-	0,120757226	36833	NM_001105181	Vmn2r68	Lx2	-880
chr7:92968595..92968596-	chr7	-	0,483028905	64604	NM_001105185	Vmn2r72	Lx2	-425
chr17:19628512..19628512+	chr17	+	0,072454336	13262	NM_001104562	Vmn2r100	Lx2A	607
chr17:19374752..19374752+	chr17	+	0,024151445	140840	NM_001104551	Vmn2r99	Lx2A1	99
chr5:109804396..109804396+	chr5	+	0,072454336	44635	NM_001104628	Vmn2r17	Lx2B	775
chr6:123635988..123635988-	chr6	-	0,024151445	34834	NM_001104637	Vmn2r22	Lx2B	101
chr7:93167931..93167931-	chr7	-	0,096605781	57438	NM_001105187	Vmn2r74	Lx2B	95
chr17:20787242..20787242-	chr17	-	0,024151445	53518	NM_001104572	Vmn2r110	Lx2B	-246
chr17:20787381..20787381-	chr17	-	0,024151445	53657	NM_001104572	Vmn2r110	Lx2B	-385
chr17:23656555..23656555-	chr17	-	0,024151445	39490	NM_001104581	Vmn2r117	Lx2B	-144
chr5:109681227..109681227-	chr5	-	0,04830289	27085	NM_001104625	Vmn2r14	Lx2B	-289
chr5:109681651..109681651+	chr5	+	0,096605781	27509	NM_001104625	Vmn2r14	Lx2B	-713
chr5:109826346..109826346+	chr5	+	0,144908671	22685	NM_001104628	Vmn2r17	Lx2B	-24
chr10:129956007..129956007-	chr10	-	0,096605781	21071	NM_001103366	Vmn2r87	Lx3_Mus	921
chr17:19882785..19882785+	chr17	+	0,024151445	27541	NM_001104565	Vmn2r103	Lx3_Mus	959
chr17:19882980..19882980+	chr17	+	0,024151445	27346	NM_001104565	Vmn2r103	Lx3_Mus	1154
chr17:20394612..20394612-	chr17	-	0,024151445	22275	NM_001104567	Vmn2r105	Lx3_Mus	1311
chr17:20394810..20394810-	chr17	-	0,193211562	22473	NM_001104567	Vmn2r105	Lx3_Mus	1113
chr17:55791253..55791276-	chr17	-	0,241514452	26782	NM_001104582	Vmn2r118	Lx3_Mus	933
chr7:10828745..10828745-	chr7	-	0,04830289	66609	NM_001105191	Vmn2r52	Lx3_Mus	605
chr7:10832029..10832029-	chr7	-	0,04830289	69893	NM_001105191	Vmn2r52	Lx3_Mus	221
chr17:57855655..57855655-	chr17	-	0,241514452	170417	NM_001104591	Vmn2r120	Lx3_Mus	-393
chr17:19261906..19261906+	chr17	+	0,024151445	253686	NM_001104551	Vmn2r99	Lx3A	97
chr17:19261970..19261970+	chr17	+	0,169060117	253622	NM_001104551	Vmn2r99	Lx3A	161
chr7:48727513..48727513-	chr7	-	0,024151445	23001	NM_177764	Vmn2r57	Lx3A	439
chr7:49359379..49359379-	chr7	-	0,096605781	44527	NM_001105056	Vmn2r59	Lx3A	1416
chr7:92584121..92584121-	chr7	-	0,169060117	19434	NM_001105182	Vmn2r69	Lx3A	1577
chr7:92593210..92593210-	chr7	-	0,108681504	28523	NM_001105182	Vmn2r69		

chr17:19447338..19447338+	chr17	+	0,024151445	68254	NM_001104551	Vmn2r99	Lx4A	253
chr17:19541594..19541594+	chr17	+	0,144908671	100180	NM_001104562	Vmn2r100	Lx4A	741
chr17:20792168..20792168+	chr17	-	0,024151445	58444	NM_001104572	Vmn2r110	Lx4A	1010
chr6:123827063..123827063+	chr6	+	0,120757226	147712	NM_019917	Vmn2r26	Lx4A	1289
chr6:123834078..123834078+	chr6	+	0,024151445	140697	NM_019917	Vmn2r26	Lx4A	1040
chr6:123844266..123844267+	chr6	-	0,144908671	40558	NM_001104641	Vmn2r25	Lx4A	869
chr6:123859016..123859016+	chr6	+	0,024151445	115759	NM_019917	Vmn2r26	Lx4A	59
chr6:123859110..123859110+	chr6	+	0,024151445	115665	NM_019917	Vmn2r26	Lx4A	153
chr17:17828287..17828287+	chr17	+	0,024151445	12617	NM_001104539	Vmn2r90	Lx4A	-675
chr6:124251365..124251365+	chr6	-	0,120757226	69062	NM_001104642	Vmn2r27	Lx4A	-339
chr7:93970786..93970786+	chr7	+	0,072454336	93072	NM_001105189	Vmn2r78	Lx4A	-379
chr10:129898507..129898507+	chr10	-	0,024151445	5056	NM_001103365	Vmn2r86	Lx4B	152
chr17:18606216..18606216+	chr17	+	0,024151445	113153	NM_001104547	Vmn2r96	Lx4B	262
chr7:92274542..92274542+	chr7	-	0,265665897	113511	NM_001033878	Vmn2r66	Lx4B	349
chr17:23466321..23466321+	chr17	+	0,096605781	14622	NM_001104579	Vmn2r115	Lx5	598
chr3:63862779..63862779+	chr3	+	0,024151445	22784	NM_019918	Vmn2r1	Lx5	41
chr3:63863287..63863287+	chr3	+	0,024151445	22276	NM_019918	Vmn2r1	Lx5	549
chr3:64019597..64019597+	chr3	-	0,024151445	78203	NM_001104592	Vmn2r2	Lx5	-244
chr3:64589993..64589993+	chr3	-	0,144908671	66477	NM_175674	Vmn2r7	Lx5	-19
chr7:93140398..93140398+	chr7	-	0,024151445	29905	NM_001105187	Vmn2r74	Lx5	-1108
chr7:93228686..93228688+	chr7	-	0,579634685	118195	NM_001105187	Vmn2r74	Lx5	-681
chr6:123916191..123916191+	chr6	+	0,024151445	58584	NM_019917	Vmn2r26	Lx6	28
chr7:49354264..49354264+	chr7	-	0,096605781	39412	NM_001105056	Vmn2r59	Lx6	134
chr7:92211281..92211281+	chr7	-	0,024151445	50250	NM_001033878	Vmn2r66	Lx6	415
chr17:22717664..22717664+	chr17	+	0,024151445	20450	NM_001104575	Vmn2r112	Lx6	-8
chr17:22721395..22721411+	chr17	+	0,04830289	16719	NM_001104575	Vmn2r112	Lx6	-15
chr6:123613896..123613896+	chr6	-	0,096605781	12742	NM_001104637	Vmn2r22	Lx6	-211
chr7:49198835..49198835+	chr7	-	0,024151445	70294	NM_001105055	Vmn2r58	Lx6	-1135
chr17:23467931..23467931+	chr17	-	0,265665897	18150	NM_001102584	Vmn2r114	Lx7	21
chr10:78786105..78786106+	chr10	+	0,096605781	33230	NM_001101572	Vmn2r82	Lx7	-11
chr3:64096467..64096467+	chr3	-	0,024151445	5133	NM_001104614	Vmn2r3	Lx7	-355
chr3:64133184..64133184+	chr3	-	0,289817343	41850	NM_001104614	Vmn2r3	Lx7	-102
chr7:49217798..49217798+	chr7	-	0,096605781	89257	NM_001105055	Vmn2r58	Lx7	-187
chr17:17788619..17788619+	chr17	+	0,120757226	52285	NM_001104539	Vmn2r90	Lx8	-55
chr3:64651122..64651122+	chr3	-	0,362271678	127606	NM_175674	Vmn2r7	Lx8	-346
chr3:64766966..64766966+	chr3	-	0,024151445	243450	NM_175674	Vmn2r7	Lx8	-596
chr17:55789955..55789955+	chr17	-	0,024151445	25484	NM_001104582	Vmn2r118	Lx9	156
chr17:55789997..55789998+	chr17	-	0,265665897	25527	NM_001104582	Vmn2r118	Lx9	113
chr17:55790097..55790097+	chr17	-	0,144908671	25626	NM_001104582	Vmn2r118	Lx9	14
chr7:10824412..10824412+	chr7	-	0,096605781	62276	NM_001105191	Vmn2r52	Lx9	87
chr7:92217556..92217556+	chr7	-	0,024151445	56525	NM_001033878	Vmn2r66	Lx9	514
chr7:93266747..93266747+	chr7	-	0,072454336	156254	NM_001105187	Vmn2r74	Lx9	429
chr17:18643755..18643755+	chr17	+	0,024151445	75614	NM_001104547	Vmn2r96	MusHAL1	115
chr17:20753276..20753276+	chr17	-	0,072454336	19552	NM_001104572	Vmn2r110	MusHAL1	33
chr17:20753778..20753778+	chr17	-	0,024151445	20054	NM_001104572	Vmn2r110	MusHAL1	220
chr7:92471731..92471731+	chr7	-	0,024151445	85016	NM_001105181	Vmn2r68	MusHAL1	554
chr7:92498784..92498784+	chr7	-	0,072454336	112069	NM_001105181	Vmn2r68	MusHAL1	52

2.2 Supplementary Figures.

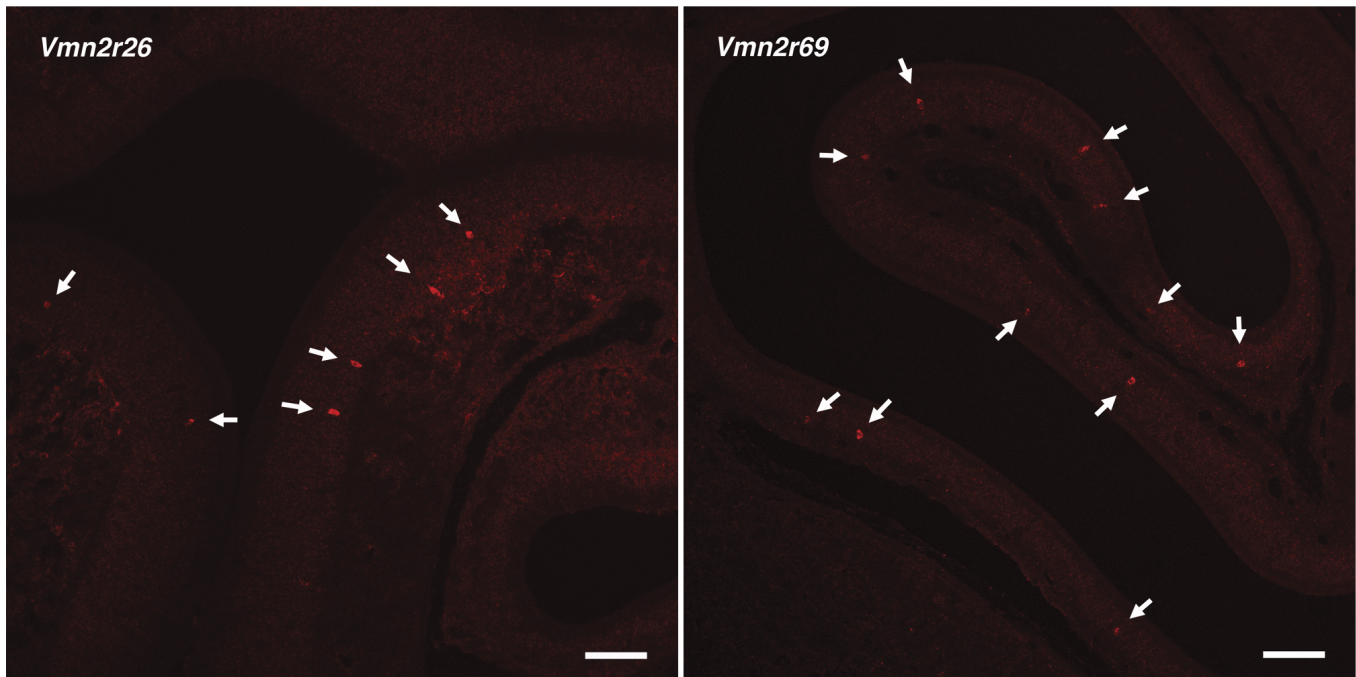

Supplementary Figure S1.

In situ hybridization results for *Vmn2r26* and *Vmn2r69* displayed on larger regions of turbinates to demonstrate that the observed expression of these two VRs transcripts is not limited to a few number of cells. Scale bars: 200µm.

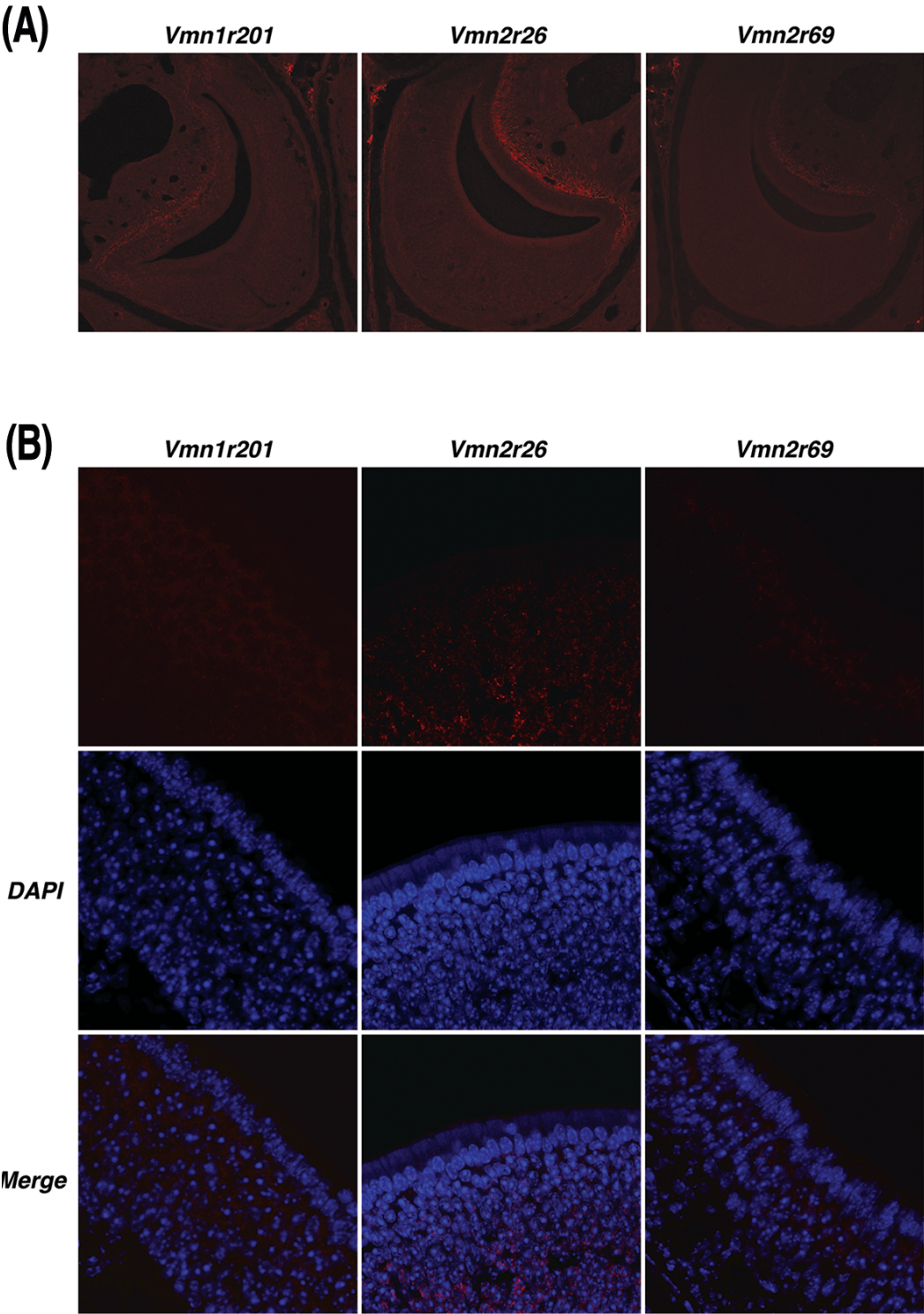

Supplementary Figure S2.

Representative negative control images of ISH performed on sections of VNO and MOE labeled with sense probes for Vmn1r201, Vmn2r26 and Vmn2r69 in the VNO (A) and in the MOE (B).

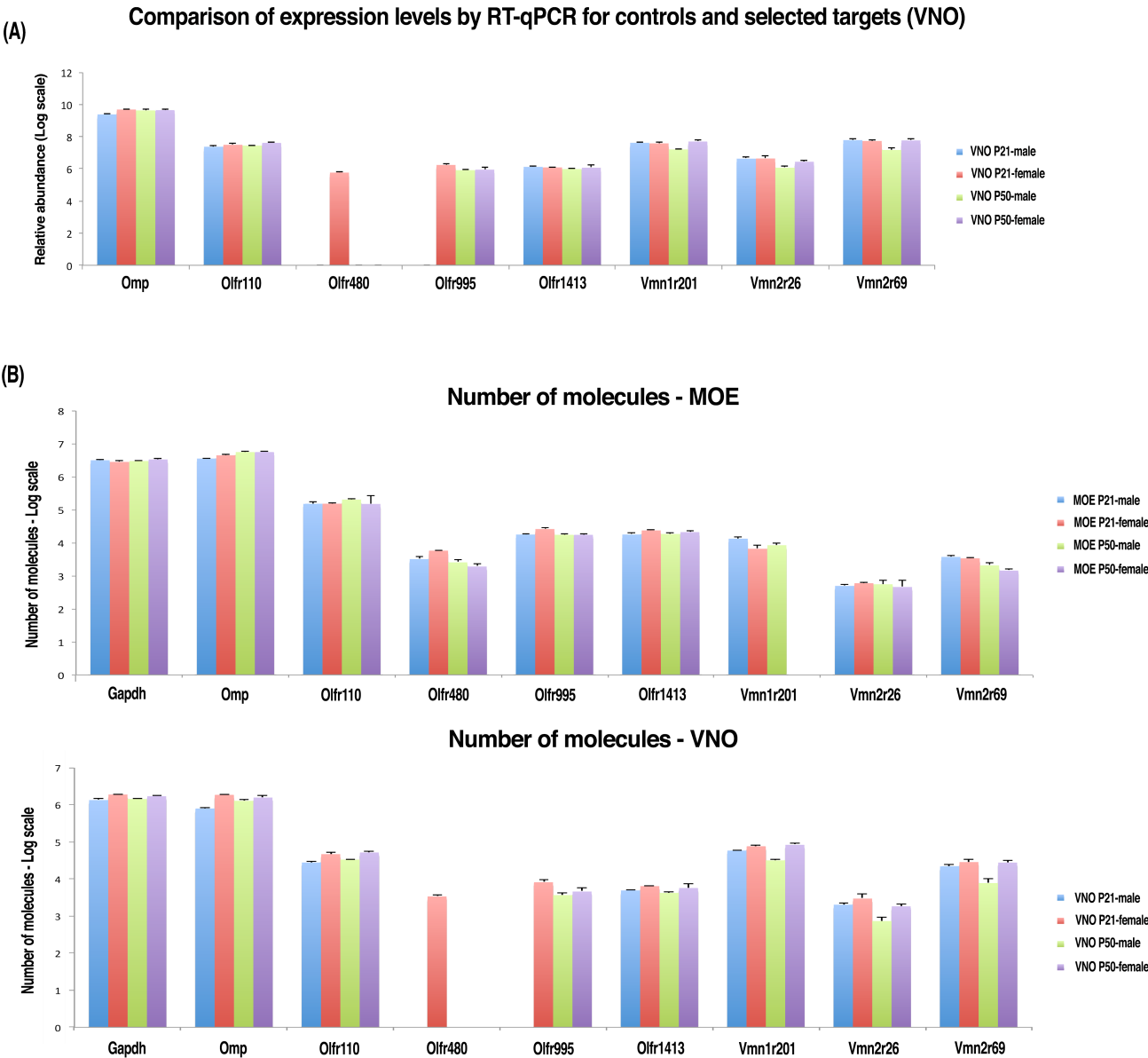

Supplementary Figure S3.

Quantitative RT-PCR data showing the expression levels of the selected targets in the VNO of all tested mice and the copy number calculation.
